# Immunopeptidomics reveals conserved and abundant *Francisella tularensis* proteins as immunogenic CD4 T cell antigens in mice

**DOI:** 10.1371/journal.ppat.1014597

**Published:** 2026-09-29

**Authors:** Pavlina Laskova, Stanislava Porkertova, Ondrej Ballek, Lucie Balonova, Jana Pavloskova, Ales Neuwirth, Ivona Pavkova, Ivo Fabrik, Pavol Lukac, Luca Vannucci, Dominik Filipp, Jiri Stulik, Marek Link

**Affiliations:** 1 Department of Molecular Pathology and Biology, Military Faculty of Medicine, University of Defence, Hradec Kralove, Czech Republic; 2 Laboratory of Immunobiology, Institute of Molecular Genetics of the Czech Academy of Sciences, Prague, Czech Republic; 3 Laboratory of Adaptive Immunity, Institute of Molecular Genetics of the Czech Academy of Sciences, Prague, Czech Republic; 4 Biomedical Research Center, University Hospital Hradec Kralove, Hradec Kralove, Czech Republic; 5 Laboratory of Immunotherapy, Institute of Microbiology of the Czech Academy of Sciences, Prague, Czech Republic; 6 Department of Cell Biology, Faculty of Science, Charles University, Prague, Czech Republic; Virginia Commonwealth University School of Medicine, UNITED STATES OF AMERICA

## Abstract

Human tularemia is caused by infection with the intracellular bacterium *Francisella tularensis*. The ability of the bacterium to cause a severe pneumonic form of the disease has led to concerns in the field of biodefense and efforts to develop a vaccine. *F. tularensis* LVS provides partial protection against fully virulent strains, with CD4^+^ T cells playing a significant role in this protection, however the recognized bacterial antigens remain largely unknown. To identify these antigens, we screened MHC class II peptides presented by infected dendritic cells using mass spectrometry-based immunopeptidomics. We identified 57 MHC-II peptides from 23 different *F. tularensis* LVS proteins. Most of these proteins were highly expressed in the bacterial proteome. Subsequent screening for IFN-γ responses identified ten peptides as immunogenic epitopes of CD4^+^ T cells following immunization of mice with *F. tularensis* LVS, thereby revealing previously unknown specificity and breadth of the induced immune responses. Five epitopes, derived from the antigens FTL_1678, DacD, DnaK, FTL_1498, and MinD, represented the dominant epitopes that elicited a response in at least 80% of immunizations. Finally, a peptide vaccine containing these epitopes proved to be immunogenic in mice and contributed to the early control of bacterial replication following challenge with the virulent *F. tularensis* FSC200 strain, although this alone was insufficient to ensure complete protection. Using immunopeptidomics, we have thus identified several immunogenic CD4^+^ T cell epitopes and antigens of *F. tularensis* LVS. Beyond their potential role in the protective immune response, the identified epitopes may also enable the development of tools for characterizing antigen-specific CD4^+^ T cells in vaccination studies or T cell biology research.

## Author summary

The establishment of effective T cell-mediated immunity is essential for the elimination of infections caused by intracellular bacterial pathogens. Identifying bacterial targets for T cells is important for determining the spectrum and specificity of immune responses, developing diagnostic tools, and designing vaccines. For the intracellular bacterium *Francisella tularensis*, the causative agent of tularemia, knowledge of the antigens and epitopes recognized by T cells is limited. In this study, we use immunopeptidomics to identify bacterial peptides presented by MHC class II from infected dendritic cells and a mouse model of tularemia to evaluate their immunogenicity. We uncovered several immunogenic epitopes and antigens of CD4^+^ T cells in the *F. tularensis* LVS strain and evaluated the protective potential of these epitopes against infection with the virulent *F. tularensis* FSC200 strain. Our study provides new insights into the specificity, dominance, and breadth of CD4^+^ T cell immune responses induced by *F. tularensis* LVS.

## Introduction

*Francisella tularensis* is a Gram-negative intracellular bacterium and the etiologic agent of the zoonotic disease tularemia. Human tularemia, caused by *F. tularensis* subsp. *tularensis* and *holarctica*, poses a health problem primarily in endemic areas and during occasional outbreaks. Although it is not a very common type of lethal infection, early diagnosis and antibiotic treatment are essential to overcome the infection without consequences. The natural reservoirs of the infection are primarily hares and small rodents, and transmission occurs through direct contact with infected animals, or via vectors such as ticks, mosquitoes, and biting flies [1]. *F. tularensis* subsp. *tularensis* is a CDC-designated Tier 1 select agent. Its low infectious dose and high mortality in the pneumonic form brought this pathogen to biodefence concerns and consequently to efforts of vaccine development [2]. The live vaccine strain (LVS) of *F. tularensis* subsp. *holarctica* is the most advanced vaccine candidate and the only one with human clinical data [3]. However, LVS remains in the investigational stage because the genetic basis of its attenuation is not fully defined, and it also provides only limited protection against aerosol infection by the most virulent strains of subsp. *tularensis*. Despite these limitations, studies using LVS have yielded key insights into protective immune responses against *F. tularensis* [3,4]. In parallel, mouse models have been widely used to study tularemia pathogenesis, as they resemble human infection in many respects. Like humans, mice can become infected by various routes and develop disseminated disease with granulomatous lesions. Mice can also be successfully vaccinated with LVS, but protection is limited against high-dose challenge with *F. tularensis* subsp. *tularensis* and wanes over time. However, significant differences exist between mouse models and human infection, particularly regarding virulence and lethality. Strains of *F. tularensis* subsp. *tularensis*, subsp. *holarctica*, and subsp. *novicida* are fatal to mice by various routes of infection; for the first two subspecies, a dose of as few as 4–10 bacteria is sufficient to cause death [5,6]. In contrast, infections with subsp. *holarctica* are rarely fatal in humans, and subsp. *novicida* is considered largely non-virulent in immunocompetent individuals [4].

*F. tularensis* primarily infects phagocytic cells and, after a brief stay in the phagosome, multiplies in the nutrient-rich cytosol and spreads throughout the host [7]. As with other intracellular bacteria, the T cell-mediated immune response plays a key role in protecting against *F. tularensis* infection [8]. Although this has been shown mainly in mice, persistent T cell proliferative responses and cytokine responses have also been reported in patients with tularemia or in persons vaccinated with LVS [9,10]. In mice, αβ T cells were essential for survival and bacterial clearance, but the relative importance of each subset varies depending on the vaccine strain, challenge strain, and route of infection used. For the LVS strain, CD4^+^ or CD8^+^ T cells alone were sufficient to resolve primary intradermal infection and subsequent lethal intraperitoneal challenge [11]. MAIT (mucosa-associated invariant T) cells also supported mice survival following intranasal inoculation with LVS but were unable to clear the bacteria in the absence of CD4^+^ and CD8^+^ T cells [12]. Both T cell subsets were essential for providing protection against infection with different strains of *F. tularensis* subsp. *tularensis* in mice that had been immunized with the LVS strain under various conditions [13–15]. The ability of immune lymphocytes or individual populations of CD4^+^, CD8^+^, and CD4^-^CD8^-^ T cells to suppress LVS growth in macrophages was also confirmed *in vitro* [16]. Further studies have shown that the efficacy of T cell-mediated control of intracellular *Francisella* growth reflects the protective potential of the vaccine and that the greater protection provided by the live attenuated SCHU S4-*ΔclpB* vaccine compared to LVS can be attributed to more effective T cell activation [17–20].

Although CD8^+^ and CD4^+^ T cells play a key role in protecting against *F. tularensis* infection, the *Francisella* antigens and epitopes targeted by these cells have not been precisely defined, and only a few studies have addressed this issue. In mice, genome-wide prediction of MHC (major histocompatibility complex) class I peptides and their subsequent screening led to the identification of 82 *Francisella* CD8^+^ T cell epitopes [21,22]. The CD4^+^ T cell epitopes of Tul4 (hereafter referred to as LpnA), GroEL, DnaK, IglB, IglC, and 50S ribosomal protein L3 were identified through screening of T cell hybridoma reactivity against a panel of recombinant proteins [23–25]. It was found that the LpnA epitope is recognized during both the acute and memory phases of primary LVS infection [23], whereas the DnaK, GroEL, and IglB epitopes were insignificant, subdominant epitopes [24]. In humans, T cell immune response to *F. tularensis* may involve a broad range of antigens. The 15 HLA-I and 24 HLA-II epitopes stimulated IFN-γ secretion in PBMCs of patients who previously had tularemia [26]. Although no single epitope appeared immunodominant, HLA-II epitopes from IglD, chitinase family 18 protein, and release factor glutamine methyltransferase were recognized in more than 43% of subjects. The three T cell stimulating peptides of LpnA were also identified in humans with previous exposure to *F. tularensis* [27]. Recently, ten bacterial HLA-I peptides have been identified from LVS infected human monocyte-derived dendritic cells, but these peptides were not evaluated for immunogenicity [28].

Mass spectrometry (MS)-based immunopeptidomics has enabled the identification of peptides bound to MHC class I and II proteins that are generated during the processing and presentation of both endogenous and foreign antigens. It is based on the isolation of MHC-peptide complexes using allele- or pan-specific monoclonal antibodies, the purification of antigenic peptides, and sequencing via liquid chromatography-tandem mass spectrometry (LC-MS/MS), followed by bioinformatics analysis [29,30]. Continuous development of the technology have enabled its translational applications [31] including those in the field of vaccine research [32]. With a focus on bacterial pathogens, immunopeptidomics has been used to discover novel T cell antigens with a protective potential for *Chlamydia* species [33,34], *Mycobacterium* species [35,36], *Salmonella enterica* [37] or *Listeria monocytogenes* [38,39].

Given the importance of *F. tularensis* LVS in studies of protective immunity and the role of T cell-mediated immunity in eliminating the infection, we decided to identify *Francisella* antigens that are recognized by T cells, specifically CD4^+^ T cells, and that could thus be involved in the protective immune response. To achieve this goal, we used immunopeptidomics to identify bacterial peptides presented by MHC-II molecules of infected bone marrow-derived dendritic cells (BMDCs). For infection, we used the *F. tularensis* LVS strain or a mutant strain with a deletion in the gene encoding disulfide oxidoreductase (LVSΔdsbA) [40]. Deletion of this gene in various *Francisella* strains results in bacteria with impaired escape from phagosomes and intracellular replication, prolonged residence in vacuoles bearing markers of the lysosomal degradation pathway, and the ability to activate BMDCs [40–43], suggesting that it may interact more strongly with the antigen processing pathway [44]. In our study, we identified 57 *Francisella* peptides from 23 different proteins, which originated mainly from a highly abundant fraction of bacterial proteome. Most of these proteins were previously unknown MHC-II antigens. We further validated the identified bacterial peptides as epitopes of CD4^+^ T cells in a mouse model of tularemia. Ten peptide epitopes exhibited immunogenicity following primary infection with *F. tularensis* LVS, with five of them representing dominant epitopes. Finally, the protective potential of the proposed peptide vaccine against infection with the *F. tularensis* FSC200 strain was tested.

## Results

### Bacterial MHC-II peptides represent rare and low abundant population of the immunopeptidome

To identify bacterial peptides presented by MHC class II molecules, we analyzed the immunopeptidome of BMDCs infected with the *F. tularensis* LVS strain or the LVSΔdsbA strain. During the initial setup of infection, we assessed bacterial growth within cells, as well as the effects on viability and the expression of surface markers. Although both strains replicated in BMDCs, the growth of the LVSΔdsbA strain was significantly lower than that of the LVS strain, with average logarithmic increases in colony-forming units of 0.64 and 2.94, respectively, after 22 hours (Fig A in S1 Appendix). The viability of BMDCs typically ranged from 50-65% 20–24 h postinfection when a multiplicity of infection of 5 was used. BMDCs upregulated the surface activation marker CD86 20–24 h after infection with either *Francisella* strain, but we were unable to detect differences in MHC-II expression between infected and uninfected cells (Fig A in S1 Appendix).

For immunopeptidomics, replicates of infection with both *Francisella* strains as well as control samples of lipopolysaccharide-stimulated BMDCs were prepared, and MHC-II associated peptides were identified by LC-MS/MS after immunoprecipitation of I-A^b^ complexes from cell lysates. The identified MHC-II peptidomes are based on the two main datasets (infected and control samples) and the two small-scale data (only LVS-infected samples) (S1 Table). In the main datasets, we identified 5,570 and 5,227 mouse MHC-II peptides with an intensity range spanning four orders of magnitude and, importantly, 47 and 39 putative *Francisella* peptides, respectively. Bacterial peptides accounted for approximately 0.8% of all identifications and 0.1% of the total peptide intensity in individual samples (Fig 1A), making them rare and low-abundance species in the MHC-II peptidome of infected BMDCs. Given the complexity of identifying non-specifically cleaved peptides and the low intensity of many bacterial peptides, which can lead to spectra with incomplete fragment ion series or fragments from co-isolated peaks, we performed manual curation of the identifications. To assess the specificity of bacterial peptide identifications, we utilized peptide extracted ion chromatograms (XICs), as these traces should be detected in infected but not uninfected control samples (Fig 1B, 1C). As a result, we excluded two bacterial peptides with low ion scores and five identifications for which peptide-spectrum matches were assigned in control samples, leading to 79 confirmed peptides. For 73 of these peptides, XICs were detected only in infected samples. Although interferences from control samples were observed in the XICs of the remaining 6 peptides, their nonspecific nature and low intensity justified acceptance of peptide identification. We thus identified 79 confident *Francisella* peptides, representing 57 non-redundant sequences and 24 distinct sequence clusters. The sequence determination of selected representatives from each cluster was further supported by spectral similarity analysis. High spectral similarity was obtained for 20 peptides (PCC ranging from 0.83 to 1) for which synthetic peptide spectra were available (Fig B in S1 Appendix), while moderate similarity was obtained for 3 peptides (PCC 0.46-0.76) for which spectra were predicted (Fig C in S1 Appendix). In the LVSΔdsbA strain, we identified more bacterial peptides presented on MHC-II than in the LVS strain (Fig 1D), suggesting improved antigen presentation. Interestingly, based on a matching of peptide features, we were able to detect many of these peptides in the immunopeptidome of LVS as well. This indicates that the peptide repertoire overlaps to a significant extent and that the differences are primarily quantitative, but further experiments would be needed to confirm this.

**Fig 1:**
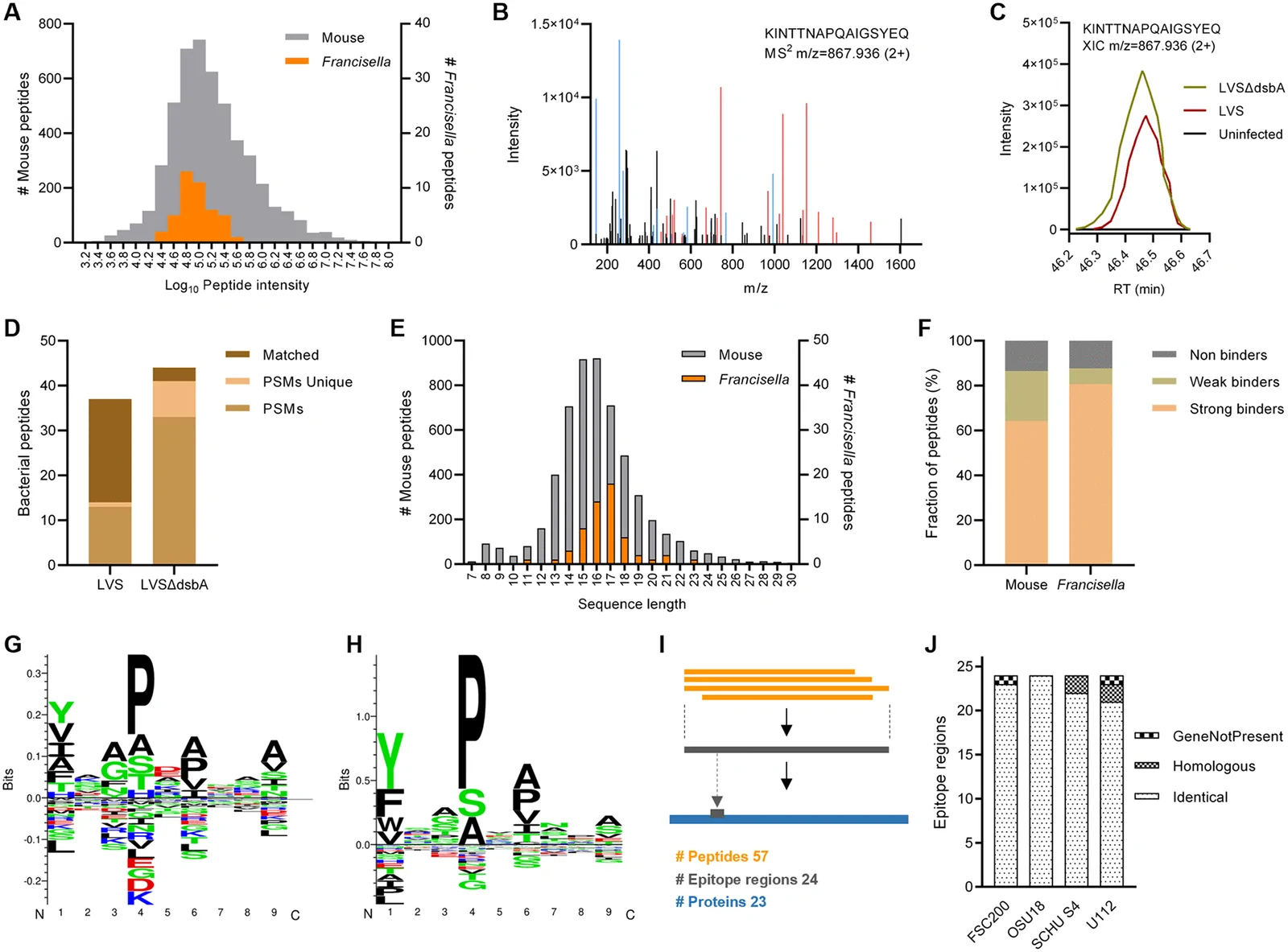
Identification and characterization of MHC-II immunopeptidome of *Francisella*-infected BMDCs. (A) Histogram showing the dynamic range of the identified immunopeptidome and the relationship between endogenous mouse (left axis) and *Francisella* (right axis) peptides. MS signal intensities of peptides identified at q-value <0.01 from a sample of BMDCs infected with LVSΔdsbA are shown. (B) MS/MS spectrum of the *Francisella* peptide KINTTNAPQAIGSYEQ with annotation of b-ions (red, including water, ammonia loss) and y-ions (blue, including water loss), obtained for the doubly protonated precursor ion. (C) Extracted ion chromatograms for the peptide identified in (B) from analyses of uninfected, LVS-infected, and LVSΔdsbA-infected BMDCs. (D) Bacterial peptides identified in BMDCs infected with LVS or LVSΔdsbA. Proportions of identifications assigned from peptide-spectrum matches (PSMs) and from peptide feature matches (matched) between LVS and LVSΔdsbA are shown. Identifications assigned from peptide-to-spectrum matches and from match-between-runs comparison are shown. (E) Sequence length distribution of identified endogenous murine (left axis, n = 5,545) and bacterial (right axis, n = 57) peptides. (F) Mouse peptides (n = 5,441, 9-30 aa in length) and *Francisella* peptides (n = 57, 11-23 aa in length) were classified as strong, weak, and non-binding based on predicted binding to the I-A^b^ molecule. %rank thresholds of <1%, < 5%, and ≥5% were used. (G) Peptide binding motif determined for bacterial peptides (n = 57) and for (H) endogenous mouse peptides (n = 5,441) using the GibbsCluster-2.0 tool. (I) The 57 *Francisella* MHC-II peptides clustered into 24 epitope regions originating from 23 different protein antigens. (J) The sequences of the identified epitope regions of *F. tularensis* LVS were evaluated for conservation in other strains of this bacterium using BLASTP. The proportions of identical, homologous, or those not found in the given strain are shown.

A total of 57 different *Francisella* peptides were identified (S2 Table). As shown in Fig 1E, most of these peptides were 16–17 amino acids in length, consistent with the length distribution of endogenous mouse peptides and characteristic of MHC-II associated peptides [45]. 80% and 7% of the bacterial peptides were predicted to be strong and weak I-A^b^ binding peptides using NetMHCIIpan binding thresholds of 1% and 5%, respectively (Fig 1F). Similarly, 64% and 22% of the endogenous peptides were classified into these categories. The binding motif derived from the bacterial peptide sequences (Fig 1G) revealed a preference for proline at position P4 and was similar to the binding motif found in endogenous peptides (Fig 1H) or previously published for this allele [46]. The bacterial peptides exhibited similar biochemical properties to endogenous peptides, suggesting that they were generated in the BMDC antigen processing and presentation pathway and thus represent genuine MHC class II presented peptides.

### Epitope regions are conserved across *Francisella* strains and display low sequence homology to eukaryotic proteins

Fifty-seven unique *Francisella* peptides were assigned to 24 epitope regions derived from 23 different antigens (Fig 1I). The term “epitope region” refers to the part of the protein from which these peptides, often detected as overlapping N- or C-terminal sequence variants, originated. Thirteen of these epitope regions were thus characterized by a single peptide, while the others were characterized by two or more peptides. The conservation of peptide sequences from *F. tularensis* LVS was analyzed in other strains of this bacterium to determine the possibility of cross-reactivity. The genomes of various *F. tularensis* strains are highly similar at the nucleotide level [47], and the sequences of most peptides were indeed identical in the subsp. *holarctica* strains FSC200 and OSU18, the subsp. *tularensis* strain SCHU S4, and the subsp. *novicida* strain U112 (Fig 1J, S2 Table). Only a few peptides contained a single amino acid substitution, and the epitope region _63_TAATSAAAPKVNLAEEISG_81_ of the AccB protein contained an additional insertion of three amino acids in its homologue from strain U112. None of these sequence differences, whether located outside or within the binding core, had a significant effect on the predicted binding of these mutated peptides to I-A^b^ compared to the LVS peptides (Table A in S1 Appendix). Importantly, the bacterial peptides showed either no or minimal sequence homology with the mouse peptides. Only three epitope regions showed sequence homology of 75%, 64%, and 62%, with up to five amino acid mismatches allowed in their sequences (S2 Table). This is a prerequisite for eliciting a T-cell response, as highly homologous bacterial peptides may be suboptimally recognized by naïve T cells [48]. The identified bacterial peptides thus have the potential to induce specific T cell reactivity in mice and to exhibit cross-reactivity in vaccination and challenge studies using heterologous *Francisella* strains.

### *Francisella* MHC-II antigens

Twenty-three antigens were identified as sources of *Francisella* MHC-II peptides, and all of them except the DnaK chaperone protein, were defined by a single epitope region (Table 1). Of these antigens, only DnaK, IglC (intracellular growth locus subunit C), and FopA1 have been described to date as antigens for CD4^+^ T cells. It is interesting to note that while the identified epitope _53_IEGGEDVTKADSATAAASVIR_73_ of IglC was identical to a previously described epitope [25], this was not the case for the DnaK epitopes _591_KALEEAFAPIAQKAYAEQ_608_ and _629_VVDADFEDVEDDKK_642_, as these epitopes originated from different regions of the protein than those previously described [24]. In contrast, our analysis did not lead to the identification of peptides from the conserved hypothetical lipoprotein (LpnA), which contains a characterized CD4^+^ T cell epitope [23]. To find functional associations of identified *Francisella* MHC-II antigens, we annotated them in the Clusters of Orthologous Genes (COG) [49] and Kyoto Encyclopedia of Genes and Genomes (KEGG) [50] databases. The largest group of proteins, containing the ribosomal proteins RpsJ, RplQ, RplA and MetG, was annotated in the COG category translation, ribosomal structure and biogenesis (Fig 2A). Several proteins were also involved in metabolic processes and were divided into the categories of lipid transport and metabolism (FabF, FabD, AccB; fatty acid biosynthesis pathway), energy production and conversion (SucC, Mdh; citrate cycle), and amino acid transport and metabolism (Ggt, GlyA; cyanoamino acid metabolism pathway). Three proteins (FTL_1678, DacD, FopA1) were classified into the cell wall/membrane/envelope biogenesis category. Orthologs of these proteins have been identified as MHC class II antigens in other bacterial pathogens, such as *Chlamydia* [33,34] and *Mycobacteria* species [35,51], *Salmonella enterica* [37], *Listeria monocytogenes* [38], and *Bordetella pertussis* [52], and some of them have been found to be immunogenic. In summary, we identified 20 novel MHC class II antigens of *Francisella* whose functional profiles suggested involvement in translation, lipid metabolism, and cell wall biogenesis.

**Table 1:** Identified MHC-II antigens of *F. tularensis* LVS. The epitope region of an antigen is represented by the longest identified peptide sequence and is annotated by the positions in the protein sequence and the number of unique peptides identified (in parenthesis). Part of the sequence is underlined if the peptide selected for synthesis was different from the longest peptide. The names of synthetic peptides and the classification of their reactivity in ELISpot assays are given (n.s.- non-significant, minor or major epitope). Protein name- name of the homolog in *F. tularensis* SCHU S4 reference proteome (UniProt). ^†^Various peptides were synthesized for the epitope region. ^‡^Positive control (not identified in our study).

Protein name	Gene names	Epitope region	Peptide name	Reactivity
Intracellular growth locus, subunit C	FTL_0113 iglC1	_53_IEGGEDVTKADSATAAASVIR_73_ (1)	iglC1-IR-21	n.s.
Small ribosomal subunit protein uS10	FTL_0235 rpsJ	_34_TGAQVKGPIPLPVRKE_49_ (2)	rpsJ-TE-16	Minor
Large ribosomal subunit protein bL17	FTL_0262 rplQ	_130_IELVDRPQVEEAADEE_145_ (1)	rplQ-IE-16	Minor
TcdA/TcdB catalytic glycosyltransferase domain protein	FTL_0265	_315_EISIFHTITINSDQ_328_ (1)		not tested
Methionine--tRNA ligase	FTL_0444 metG	_474_VCSQGINMFKVLIAYLK_490_ (1)		not tested
Septum site-determining protein MinD	FTL_0519 minD	_141_DAAIIVTNPEVSSVRDSDR_159_ (6)	minD-IR-16	Major
Outer membrane protein 26	FTL_0569 omp26	_20_DSYTMYAKPDTKSPKLATIDDQD_42_ (1)		not tested
DUF6844 domain-containing protein	FTL_0572	_88_RDRYDYVGSAIGTASVN_104_ (1)		not tested
Serine hydroxymethyltransferase	FTL_0703 glyA	_92_ANVQPHSGSQANAAVYN_108_ (3)	glyA-NN-16	n.s.
		_92_ANVQPHSGSQANAAVYN_108_^†^	glyA-VN-15	n.s.
Glutathione hydrolase proenzyme	FTL_0766 ggt	_581_DGQYVYGAADPRRASAL_597_ (1)	ggt-DL-17	n.s.
Beta-lactamase	FTL_0879	_156_ADEPEINYTQPHSNINK_172_ (2)	ftl0879-AK-17	n.s.
Malate dehydrogenase	FTL_0987 mdh	_226_LKTGSAYYAPAAAGIQM_242_ (1)	mdh-LM-17	n.s.
Peptidyl-prolyl cis-trans isomerase	FTL_1042	_173_VTIAYKGTTPVIAYEDDK_190_ (5)	ftl1042-VD-17	Minor
Serine-type D-Ala-D-Ala carboxypeptidase	FTL_1060 dacD	_201_RSYIYNFPEAYKVYDDK_217_ (1)	dacD-RK-17	Major
3-oxoacyl-[acyl-carrier-protein] synthase 2	FTL_1137 fabF	_204_AIGMGGFAAARALSTRNDDPQ_224_ (2)	fabF-IQ-20	Minor
Malonyl CoA-acyl carrier protein transacylase	FTL_1140 fabD	_216_LNKVEFKEPTTAVVQNFD_233_ (8)	fabD-LD-18	n.s.
Chaperone protein DnaK	FTL_1191 dnaK	_591_KALEEAFAPIAQKAYAEQ_608_ (4)	dnaK-AE-16	Major
Chaperone protein DnaK	FTL_1191 dnaK	_629_VVDADFEDVEDDKK_642_ (2)	dnaK-VK-14	n.s.
Outer membrane associated protein	FTL_1328 fopA1	_375_PRNQRVEASVSAPLKEAN_392_ (1)	fopA1-PN-18	n.s.
Translation initiation inhibitor	FTL_1498	_4_IKINTTNAPQAIGSYEQA_21_ (6)	ftl1498-KE-15	Major
Succinate--CoA ligase [ADP-forming] subunit beta	FTL_1553 sucC	_22_GIVAHNPNEAAQAFDQ_37_ (1)	sucC-GQ-16	n.s.
Biotin carboxyl carrier protein of acetyl-CoA carboxylase	FTL_1592 accB	_63_TAATSAAAPKVNLAEEISG_81_ (4)	accB-AS-17	n.s.
LD-carboxypeptidase	FTL_1678	_70_YPSDFGYSNPDSIRAK_85_ (1)	ftl1678-YK-16	Major
Large ribosomal subunit protein uL1	FTL_1747 rplA	_76_VAVFAKGPAADAAKEAG_92_ (1)	rplA-VG-17	Minor
Conserved hypothetical lipoprotein^‡^	FTL_0421 lpnA	_86_RLQWQAPEGSKCHD_99_	Positive	Major

**Fig 2:**
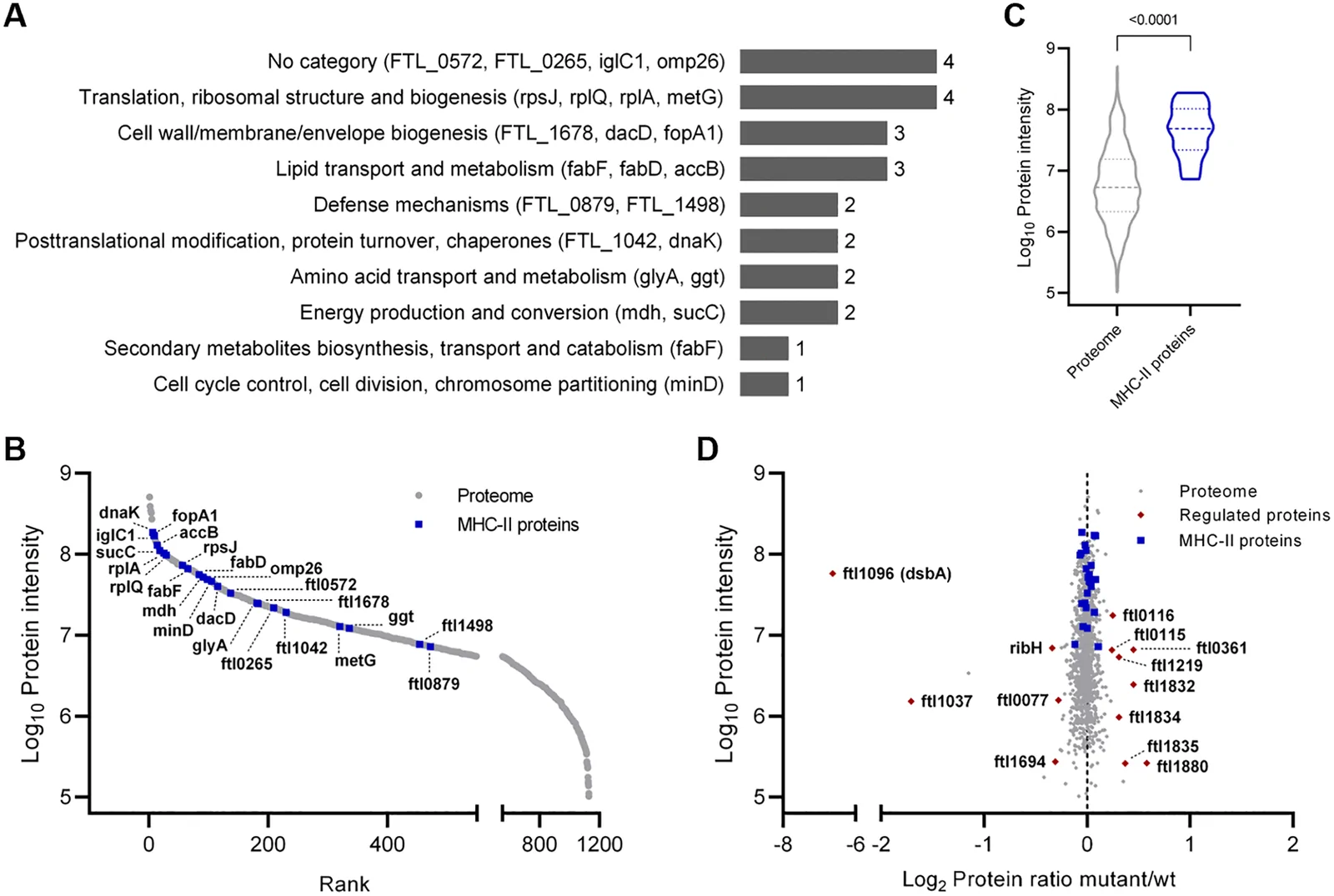
*Francisella* MHC-II antigens and their relationship to the proteome. (A) COG annotation of *Francisella* LVS proteins entering the MHC class II pathway. Proteins associated with translation, cell wall, and lipid metabolism have emerged as important categories of proteins for antigen presentation. (B) Proteome analysis of *in vitro* cultured *Francisella* LVS. The 1,131 proteins identified at 1% FDR were ranked by their intensity (1,129 quantifiable) to express their relative abundance in the proteome. Most MHC-II antigens (squares) were highly expressed proteins. (C) A violin plot showing the intensity distribution for a group of 1,129 LVS proteins or 23 MHC-II proteins. The difference between the two groups was statistically significant (two-tailed Mann-Whitney test). The median, 25th, and 75th percentiles are shown. (D) Relative quantification of proteome changes induced by deletion of the *dsbA* gene in *Francisella* LVS. The ratios of mutant and wild-type proteins (n = 1,129) were plotted against the intensities of wild-type proteins. The localization of MHC-II proteins (squares) near the line indicating equal expression in both strains confirmed their unchanged availability for antigen presentation. In the otherwise unchanged LVSΔdsbA proteome, only a few proteins with altered expression were detected (diamonds). Proteins of interest are indicated by FTL numbers or gene names in all figures.

### *Francisella* MHC-II antigens originate from a highly abundant fraction of the bacterial proteome

We performed a proteomic analysis of the LVS and LVSΔdsbA strains to determine whether there was a relationship between the discovered MHC-II antigens and the proteome. We ranked the 1,131 identified proteins (S3 Table) by intensity to express their relative abundance in the proteome. MHC-II antigens were enriched in the high-abundance fraction of the proteome (Fig 2B), and this observed pattern was statistically significant (Fig 2C). To identify changes induced by the *dsbA* gene deletion, we compared the proteomes of LVSΔdsbA and LVS using label-free quantitation. In this analysis, we confirmed the deletion of the *dsbA* gene and detected several proteins with regulated expression, but none of them belonged to the MHC-II antigens, which in fact lay close to the line indicating equal expression in both strains (Fig 2D). Regarding the bacterial proteome, we have thus demonstrated that the identified *Francisella* antigens are highly expressed and that the availability of these antigens is the same in both strains used for infection.

### *Francisella* MHC-II peptides elicit time-dependent reactivity in immune splenocytes and lymph nodes

Sublethal infection with *F. tularensis* LVS induces protective immunity against lethal challenge with homologous or heterologous *Francisella* strains, involving various populations of αβ T cells, including CD4^+^ T cells, and the cytokines IFN-γ and TNF-α [4,8]. In the following experiments, we therefore sought to determine whether any of the MHC-II peptides identified *in vitro* represent an immunogenic target for CD4^+^ T cells. For this purpose, C57BL/6 mice were subcutaneously immunized with LVS, immune lymphocytes were isolated and stimulated *ex vivo* with corresponding synthetic peptides. IFN-γ production was measured as a marker of CD4^+^ T cell activation. Twenty-one bacterial peptides (Table 1), representing 20 different epitope regions, were selected for synthesis. This selection was based exclusively on data from mass spectrometry, and in cases where multiple peptides were identified for a given epitope region, the most frequently sequenced or most abundant peptide was selected.

For initial testing, whole-cell suspensions from spleens of immunized mice were stimulated with peptides arranged in three different pools (subset of 15 peptides, Table B in S1 Appendix) and the number of IFN-γ-producing cells was measured by ELISpot. Importantly, peptide pools (PP) elicited a statistically significant response in immune splenocytes collected 4 weeks postimmunization (p.i.) (Fig D in S1 Appendix). In the following experiments, splenocytes were isolated at 3, 4, and 5 weeks after immunization and stimulated with PPs to gain insight into dynamics of the response (Fig 3A). The highest number of IFN-γ-producing cells was detected 3 weeks p.i., followed by a gradual decrease with time after immunization. Regardless of the time interval examined, a general order of reactivity of PP-1 ≈ PP-2 < PP-3 was observed, suggesting that PP-3 is most effective in stimulating IFN-γ-producing cells. No bacteria were detected in the spleens and lymph nodes (LN) at 3 weeks p.i. as determined by CFU counting of plated organ suspensions. To verify that CD4^+^ T cells were the responding subpopulation, peptide stimulations were performed in cocultures of purified CD4^+^ T cells and BMDCs, which were used as antigen presenting cells. These experiments confirmed the production of IFN-γ by CD4^+^ T cells and revealed similar PPs reactivity profiles as in whole splenocytes (Fig 3B). IFN-γ-producing cells provoked by peptide stimulation were detected in immune cells only (splenocytes or CD4^+^ T cells), and not in cells from control PBS-receiving mice (Fig 3C, 3D), verifying that the measured responses were specific and due to immunization with LVS. These experiments were performed with PP-3 that showed the most significant and reproducible responses in previous assays. Significant accumulation of IFN-γ as well as TNF-α was also confirmed in the supernatants of immune splenocytes stimulated with PP-3 by ELISA (Fig 3E, 3F). The temporal profile of immune responses was further evaluated in whole-cell suspensions and in CD4^+^ T cells from LNs of immunized mice. Except for lower response levels, the hierarchy and reactivity profiles of PPs were similar to those observed in the spleen (Fig D in S1 Appendix). In conclusion, all PPs elicited IFN-γ secretion in both LVS-immune splenocytes and LNs. This secretion was highest at 3 weeks p.i. and was mediated by CD4^+^ T cells. The PP-3 stimulated the most IFN-γ-producing cells under all conditions tested, indicating that it contains the most potent stimulatory peptide(s).

**Fig 3:**
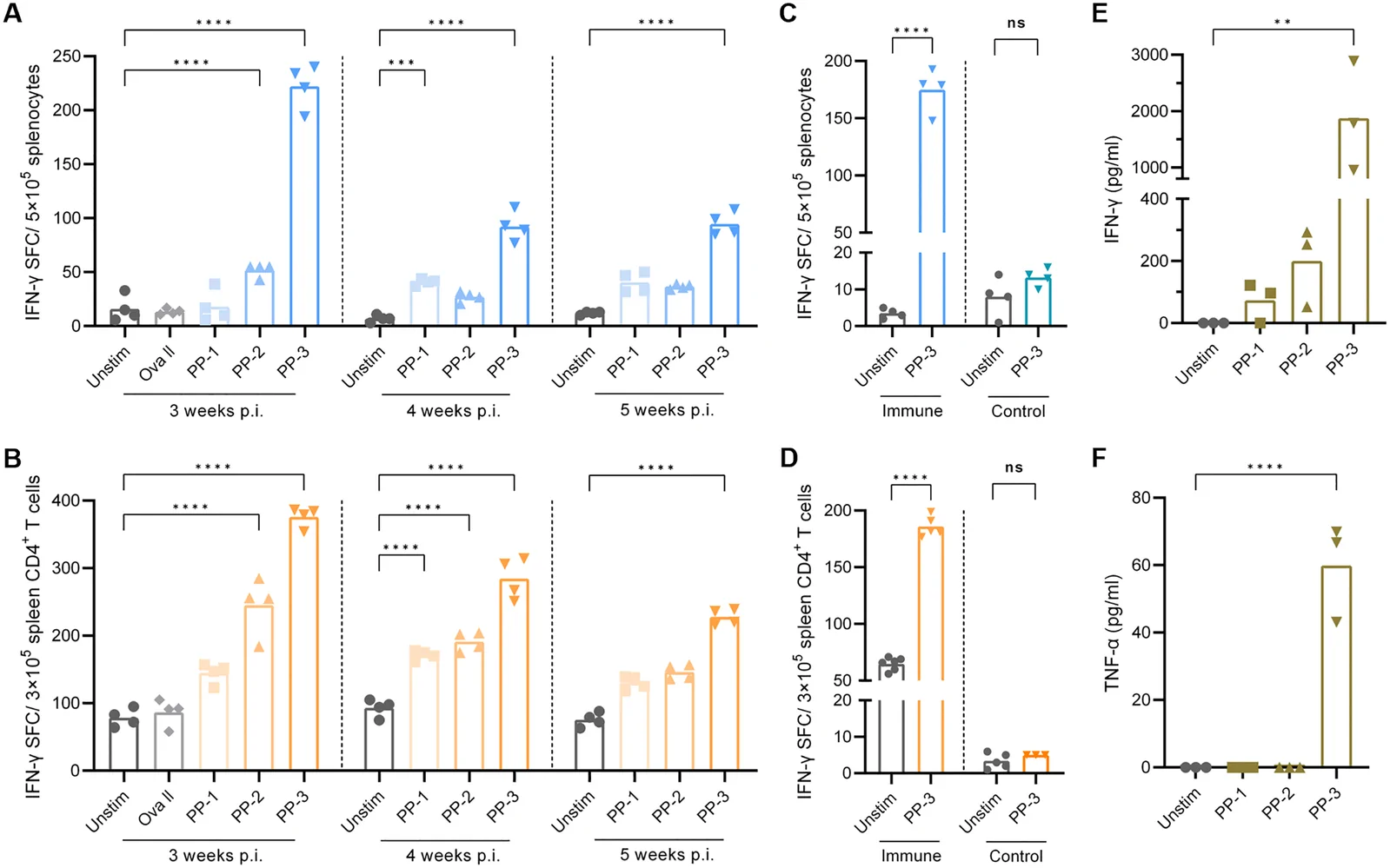
Pools of *Francisella* MHC-II peptides elicit time-dependent, specific and CD4^+^ T cell-mediated IFN-γ response in immune splenocytes. C57Bl/6 mice were subcutaneously injected with 3 × 10^4^ CFU of *F. tularensis* LVS and immune lymphocytes, pooled from groups of 3-4 mice, were stimulated with bacterial MHC-II peptides assembled in pools for immunogenicity assessment. The numbers of IFN-γ producing cells (SFCs) were determined at 3, 4 and 5 weeks p.i. following stimulation of (A) 5 × 10^5^ whole splenocytes or (B) cocultures of 3 × 10^5^ spleen CD4^+^ T cells and 3 × 10^4^ BMDCs with peptide pools (PP). Data of individual assayed wells per condition and mean values are shown. PPs with significant response against control without peptides (unstim) were detected with one-way ANOVA followed by Sidak’s post hoc test. The specificity with which the peptide-induced IFN-γ production depended on immune status of lymphocytes was verified by stimulation of (C) 5 × 10^5^ whole splenocytes (3 weeks p.i.) or (D) cocultures of 3 × 10^5^ spleen CD4^+^ T cells (4 weeks p.i.) and 3 × 10^4^ BMDCs with PP-3. Lymphocytes were isolated from LVS immunized (immune) or age-matched control (control) mice. Data of individual assayed wells per condition and mean values are shown. Responses were assessed for significant difference using a two-tailed t-test. (E) IFN-γ and (F) TNF-α concentrations were determined by ELISA in cell culture supernatants of 8 × 10^5^ LVS-immune splenocytes (3 weeks p.i.) after 48 h of PPs stimulation. Data of 3 biological replicates and mean values are shown. PPs with significant response against control without peptides were detected with one-way ANOVA followed by Dunnett’s post hoc test. PPs representing a subset of 15 peptides (Table B in S1 Appendix) were used for all assays with 5 µM final concentration of each peptide. Ova II peptide (5 µM) was used as a negative control in some experiments. Asterisks indicate p-values (**p < 0.01, ***p < 0.001, ****p < 0.0001, ns for p ≥ 0.05). Data are results of one experiment (A, B, C) or representative results of two biological replicates (D).

### Identification of immunogenic CD4^+^ T cell epitopes of *F. tularensis* LVS

Based on the results obtained with PPs, the potential of individual *Francisella* peptides to induce IFN-γ-producing cells in LVS-immune lymphocytes was examined. Spleen lymphocytes were collected 3 weeks after immunization unless otherwise stated. The screening was performed using whole splenocytes or isolated CD4^+^ T cells in coculture with naïve B cells. B cells were used instead of BMDCs in all subsequent assays because they showed lower background levels of IFN-γ while maintaining a specific response and thus increased the sensitivity of the ELISpot measurements (Fig E in S1 Appendix). Net reactivity of individual peptides (peptide - control induced IFN-γ-producing cells) measured in splenocytes or CD4^+^ T cell cocultures for 4 independent immunizations are shown in Fig 4A and Fig 4B, respectively. Representative data for each cell type are provided in Fig F in S1 Appendix. In splenocytes, 7 peptides induced a statistically significant stimulation of IFN-γ-producing cells in at least one of the immunizations. These peptides, along with 3 others, also induced reactivity in CD4^+^ T cell cocultures. Selected reactive peptides were subsequently assayed with lymphocytes from control mice that received PBS. No significant induction of IFN-γ-producing cells was detected in either splenocytes (Fig 4C) or CD4^+^ T cell cocultures (Fig 4D), confirming that the reactivity of the peptides with immune lymphocytes is specific and a consequence of immunization with the LVS. In conclusion, the ability of the peptides to stimulate both splenocytes and CD4^+^ T cells confirms their role as immunogenic epitopes of CD4^+^ T lymphocytes *in vivo*.

**Fig 4:**
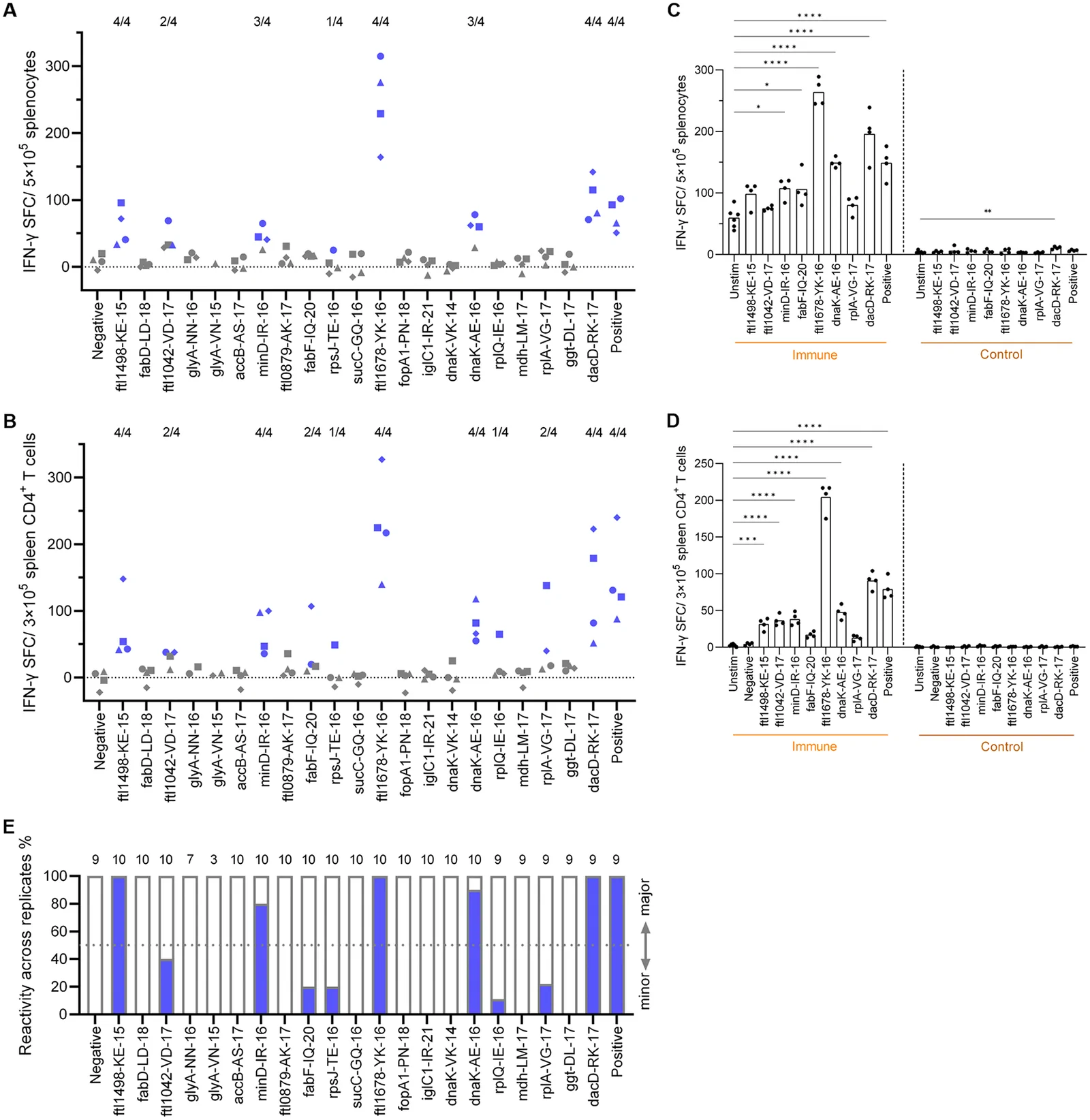
Identification of reactive *Francisella* MHC-II peptides and their validation as CD4^+^ T cell epitopes. C57Bl/6 mice were subcutaneously injected with 3 × 10^4^ CFU of *F. tularensis* LVS and immune lymphocytes, pooled from groups of 3-4 mice, were stimulated with individual bacterial MHC-II peptides to assess immunogenicity. The numbers of IFN-γ producing cells (SFCs) were determined following stimulation of (A) 5 × 10^5^ whole splenocytes or (B) cocultures of 3 × 10^5^ spleen CD4^+^ T cells and 2 × 10^5^ naïve B cells with individual peptides. Data of 4 biological replicates (unique symbols) are shown for each suspension. The numbers of IFN-γ SFCs represent net reactivity of peptides (mean reactivity of stimulated cells – mean reactivity of control cells) in individual biological experiments. Within the biological experiment, peptides with significant response were detected with one-way ANOVA followed by Dunnett’s post hoc test (p < 0.05, blue symbols). The number of times the response was significant across biological replicates is annotated in the graphs. The specificity with which the peptide-induced IFN-γ production depended on the immune status of lymphocytes was verified by stimulation of (C) 5 × 10^5^ whole splenocytes or (D) cocultures of 3 × 10^5^ spleen CD4^+^ T cells and 2 × 10^5^ naïve B cells with a selected set of peptides. Lymphocytes were isolated from LVS immunized (immune) or age-matched control (control) mice. Data of individual assayed wells per condition and mean values are shown. Peptides with significant response against control without peptide (unstim) were detected with one-way ANOVA followed by Dunnett’s post hoc test. Asterisks indicate p-values (*p < 0.05, **p < 0.01, ***p < 0.001, ****p < 0.0001). Data are results of one experiment. (E) Reactivity of peptides across biological replicates. Peptides were tested for a significant response within each biological replicate and the frequency with which the response was significant or not is shown. The number of all replicates is indicated above the columns and in addition to the data shown in Fig 4A, 4B (8 replicates), two additional replicates were included, one of them with an incomplete set of peptides. For all assays, lymphocytes were isolated 3 weeks p.i. and peptides were used at a final concentration of 10 µM. Peptide RLQWQAPEGSKCHD (*Francisella* LpnA) and YAIAVNDLGTEYVHR (mouse Gls) was used as a positive and negative peptide, respectively.

A total of 10 peptides were identified as immunogenic epitopes of CD4^+^ T cells in at least one of the 9–10 immunizations performed (Fig 4E, Table 1). Based on the frequency of statistically significant response as a criterion, we divided the peptides into three groups- nonimmunogenic (never positive), minor epitopes (positive in at least one, but max. in 50% of immunizations), and major epitopes (positive in more than 50% of immunizations) [53]. Using this definition, we found 5 minor and 5 major CD4^+^ T cell epitopes of *Francisella* LVS. Minor epitopes are FTL_1042-VD-17 (VTIAYKGTTPVIAYEDD), FabF-IQ-20 (IGMGGFAAARALSTRNDDPQ), RpsJ-TE-16 (TGAQVKGPIPLPVRKE), RplA-VG-17 (VAVFAKGPAADAAKEAG), and RplQ-IE-16 (IELVDRPQVEEAADEE). For major epitopes, the general order of reactivity was FTL_1678-YK-16 (YPSDFGYSNPDSIRAK)> DacD-RK-17 (RSYIYNFPEAYKVYDDK)> DnaK-AE-16 (ALEEAFAPIAQKAYAE) ≈ FTL_1498-KE-15 (KINTTNAPQAIGSYE) ≈ MinD-IR-16 (IIVTNPEVSSVRDSDR). The reactivity of individual peptides also explains the hierarchy of reactivity among peptide pools, as the peptides FTL_1498-KE-15 and FTL_1042-VD-17, the peptide MinD-IR-16, FabF-IQ-20 and RpsJ-TE-16, and the peptides FTL_1678-YK-16 and DnaK-AE-16 were components of PP-1, PP-2, and PP-3. In our tests, we further confirmed the previously characterized peptide epitope LpnA-RD-14 (RLQWQAPEGSKCHD) [23] as a significant epitope of *F. tularensis* LVS. Compared to LpnA-RD-14, the reactivity of FTL_1678-YK-16 was superior, and the epitopes DacD-RK-17, DnaK-AE-16, and FTL_1498-KE-15 induced IFN-γ at least as effectively, confirming the significance of the newly discovered epitopes.

Finally, the phenotype of antigen-specific CD4^+^ T cells was determined by FACS using a mixture of the most reactive peptides (PP-4) (Table B in S1 Appendix). As expected, and as confirmed by the ELISpot assay, PP-4 induced strong IFN-γ secretion only in LVS-immune CD4^+^ T cells (Fig 5A). FACS analysis supported this finding by showing that specifically CD4^+^ T cells isolated from the spleen of LVS-immunized mice respond to PP-4 by producing IFN-γ (Fig 5B). Further characterization revealed that the main source of IFN-γ is predominantly the effector CD44^+^ CD62L^-^ subpopulation of CD4^+^ T cells (Fig 5C, Fig G in S1 Appendix). By analyzing whole spleen, we also showed that, compared to PBS-control mice, LVS infection did not affect the frequency of different CD4^+^ T cell subpopulations (naïve CD44^-^ CD62L^+^ vs. central memory CD44^+^ CD62L^+^ vs. effector T cells CD44^+^ CD62L^-^) in splenocytes, which were the source of CD4^+^ T cells for all subsequent coculture assays (Fig G in S1 Appendix).

**Fig 5:**
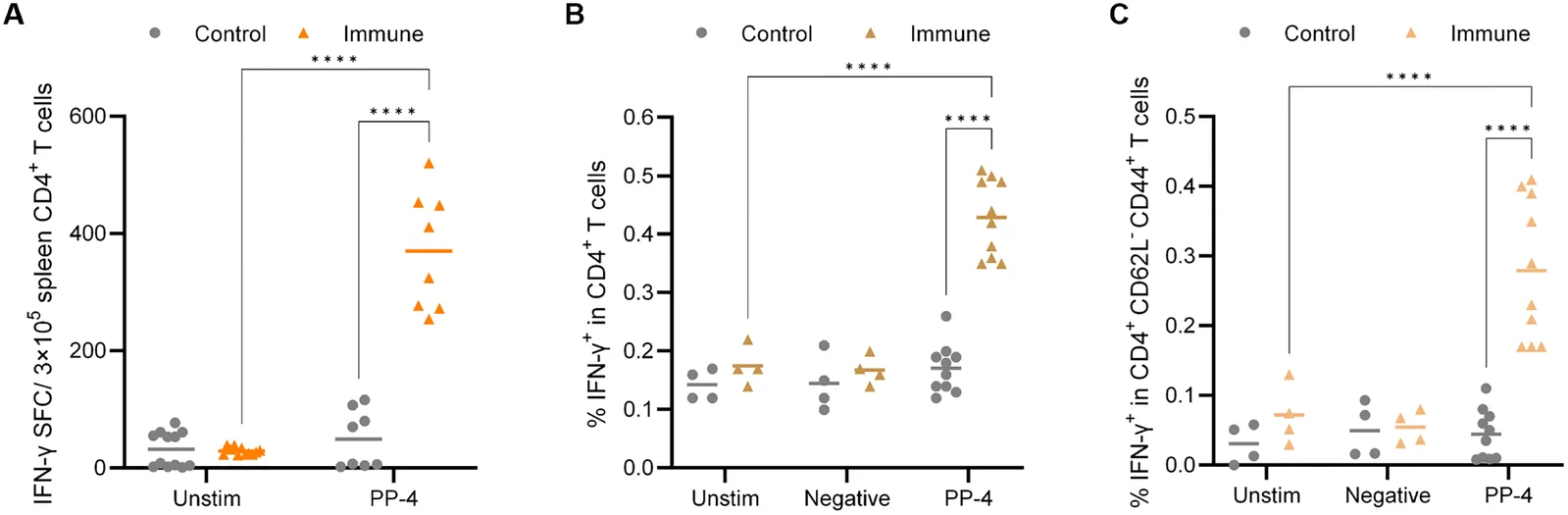
A subpopulation of effector CD4^+^ T cells is primarily responsible for reactivity to immunogenic *Francisella* epitopes. C57Bl/6 mice were injected subcutaneously with either 3 × 10^4^ CFU of *F. tularensis* LVS (immune) or sterile PBS (control), and lymphocytes were isolated 3 weeks later and pooled from groups of 4 mice. Cocultures of 3 × 10^5^ splenic CD4^+^ T cells and 2 × 10^5^ naïve B cells were stimulated for 24 h with a mixture of 5 selected immunogenic peptides (PP-4) (Table B in S1 Appendix) or left without peptides (unstim) and the IFN-γ response was determined. A concentration of 5 µM was used for each peptide. (A) The numbers of IFN-γ producing cells (SFCs) were determined using ELISpot. Furthermore, the % of intracellular IFN-γ-positive cells among live (B) total CD4^+^ T cells or (C) subpopulation of CD4^+^CD62L^-^CD44^+^ effector T cells was determined by FACS analysis. Each dot represents one technical replicate and mean values are shown. Differences between groups were determined with two-way ANOVA followed by Tukey’s post hoc test. Asterisks indicate p-values (****p < 0.0001). Peptide YAIAVNDLGTEYVHR (mouse Gls) was used as a negative peptide (negative). Data are combined results of two biological replicates.

### A vaccine containing *Francisella* peptide epitopes is immunogenic in mice

In the next experiments, we evaluated the immunogenicity of the peptide antigens themselves, which is a prerequisite for assessing their functional potential. Mice were therefore immunized with a peptide vaccine containing five major peptide epitopes of *Francisella* (FTL_1498-KE-15, MinD-IR-16, FTL_1678-YK-16, DnaK-AE-16, DacD-RK-17) (Fig 6A). Since these peptides are CD4^+^ T cell epitopes 15–17 amino acids in length, we analyzed the induced cellular response as an indicator of successful immunization. In lymphocytes from the spleen (Fig 6B) or lymph nodes (Fig H in S1 Appendix) of immunized mice, restimulation with each peptide led to a statistically significant number of IFN-γ-producing cells, and none of the peptides dominated this response. This reactivity was specific and resulted from immunization with the peptide vaccine, as no significant responses were detected in lymphocytes from control mice that received the same adjuvant but without peptides (Fig 6B). It should be noted that some immunized mice exhibited low but statistically significant reactivity or increased reactivity to a single peptide (Fig I in S1 Appendix), which led to variability in responses. Interestingly, the IFN-γ response to selected peptides in splenocytes from vaccinated mice was, on average, twenty times higher than in mice immunized with *F. tularensis* LVS (Fig 4A), suggesting quantitative differences in the induced T cell responses. Furthermore, the peptide vaccine induced a Th1-type response, as evidenced by the significant production of not only IFN-γ but also IL-2 and TNF-α following peptide stimulation of splenic lymphocytes (Fig 6C-6E). Overall, we provide evidence that the selected *Francisella* peptide epitopes are immunogenic and that the peptide vaccine induced a strong polyepitope cellular response.

**Fig 6:**
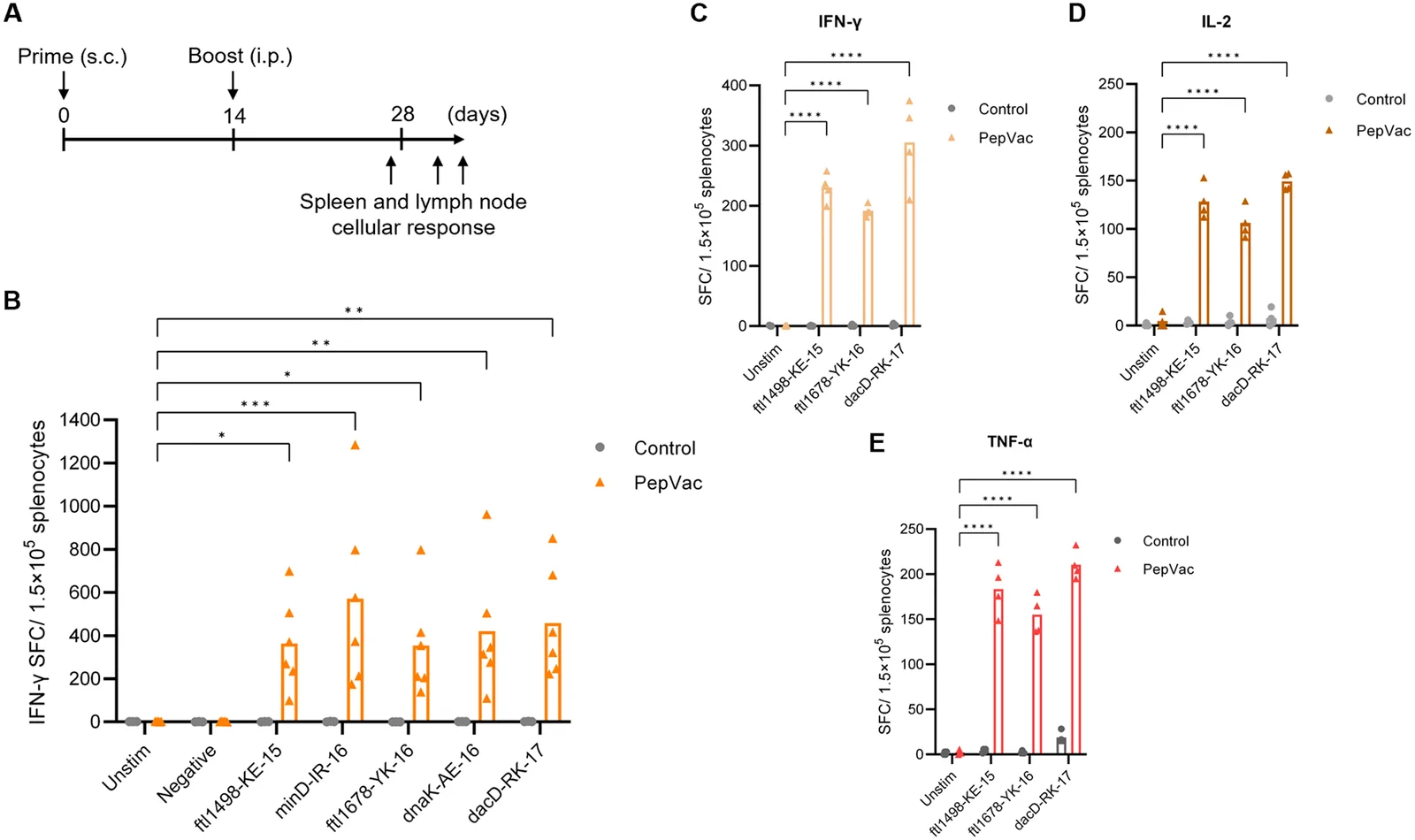
A peptide vaccine containing bacterial epitopes of CD4^+^ T cells is immunogenic in mice. (A) C57Bl/6 mice were immunized with a peptide vaccine containing five peptide epitopes of *Francisella* (FTL_1498-KE-15, MinD-IR-16, FTL_1678-YK-16, DnaK-AE-16, DacD-RK-17), and the cellular response in the spleen and lymph nodes was assessed using the ELISpot assay. A group of mice (control) received the same adjuvant formulations but without peptides. (B) Number of IFN-γ-producing cells in splenocytes following stimulation with vaccine-contained peptides. Responses were determined in individual mice from the peptide vaccine group (PepVac, n = 6) or the control group (control, n = 3). Measurements were performed on days 13, 17 and 19 after the booster dose (due to capacity reasons) and the data are combined in this graph. Values for individual mice (points) and their averages are shown. Note: The unequal group sizes were due to the limited availability of animals. (C-E) Number of cells producing IFN-γ, IL-2 and TNF-α in splenocytes following stimulation with a subset of peptides contained in the vaccine. Splenocytes were isolated 19 days after administration of the booster dose and pooled from groups of 3 mice. Data from individual wells for each condition and the corresponding mean values are shown. For all assays, peptides were used at a final concentration of 5 µM, and control wells without peptide (unstim) or with a negative peptide (YAIAVNDLGTEYVHR) were included. Significant differences compared to the control (unstim) were identified using two-way ANOVA followed by Dunnett’s post hoc test. Asterisks indicate p-values (*p < 0.05, **p < 0.01, ***p < 0.001, ****p < 0.0001). This experiment was performed once.

### Protective efficacy of peptide vaccine against *F. tularensis* FSC200 infection

After the peptide vaccine induced a Th1-type cellular immune response, we tested its protective potential against infection with the *F. tularensis* subsp. *holarctica* FSC200 strain. This strain is highly virulent to mice infected by any route, and the lethal dose after subcutaneous inoculation is < 5 bacteria [6]. The subcutaneous route of infection, which is more significant in terms of public health risks than in terms of biological defense threats associated with pulmonary infection, was used in the study for reasons of biological consistency, since the screening of immunogenic T cell epitopes was based on a subcutaneous infection model. The five peptide epitopes contained in the vaccine also have conserved sequences in the FSC200 strain, which is essential for their recognition during infection and for triggering an immune response. Four weeks after the start of vaccination, mice from the control group and mice vaccinated with the peptide vaccine were subcutaneously infected with 53 CFU of *F. tularensis* FSC200, and the course of the infection was monitored (Fig 7A). The pattern of weight loss differed significantly between the vaccinated and control mice, even though the differences at individual time points were not statistically significant. The vaccinated mice thus exhibited a more gradual decline in weight than the control mice, suggesting a slower progression of the infection (Fig 7B). Vaccinated mice also showed a slight delay in mortality (days 6–9 postinfection, median survival 7 days) compared to control mice (days 6–7 postinfection, median survival 6 days), although this difference was not statistically significant (Fig 7C). Consistent with these trends, peptide vaccination reduced the bacterial burden in organs, as evidenced by a decreased bacterial count in both the spleen and liver on day 4 postinfection (Fig 7D, 7E). In conclusion, immunization of mice with the peptide vaccine induced cellular immunity against T cell peptide epitopes, which contributed to early control of the bacterial load and reduced disease severity, although this alone was insufficient to provide full protection against lethal infection with the virulent FSC200 strain.

**Fig 7:**
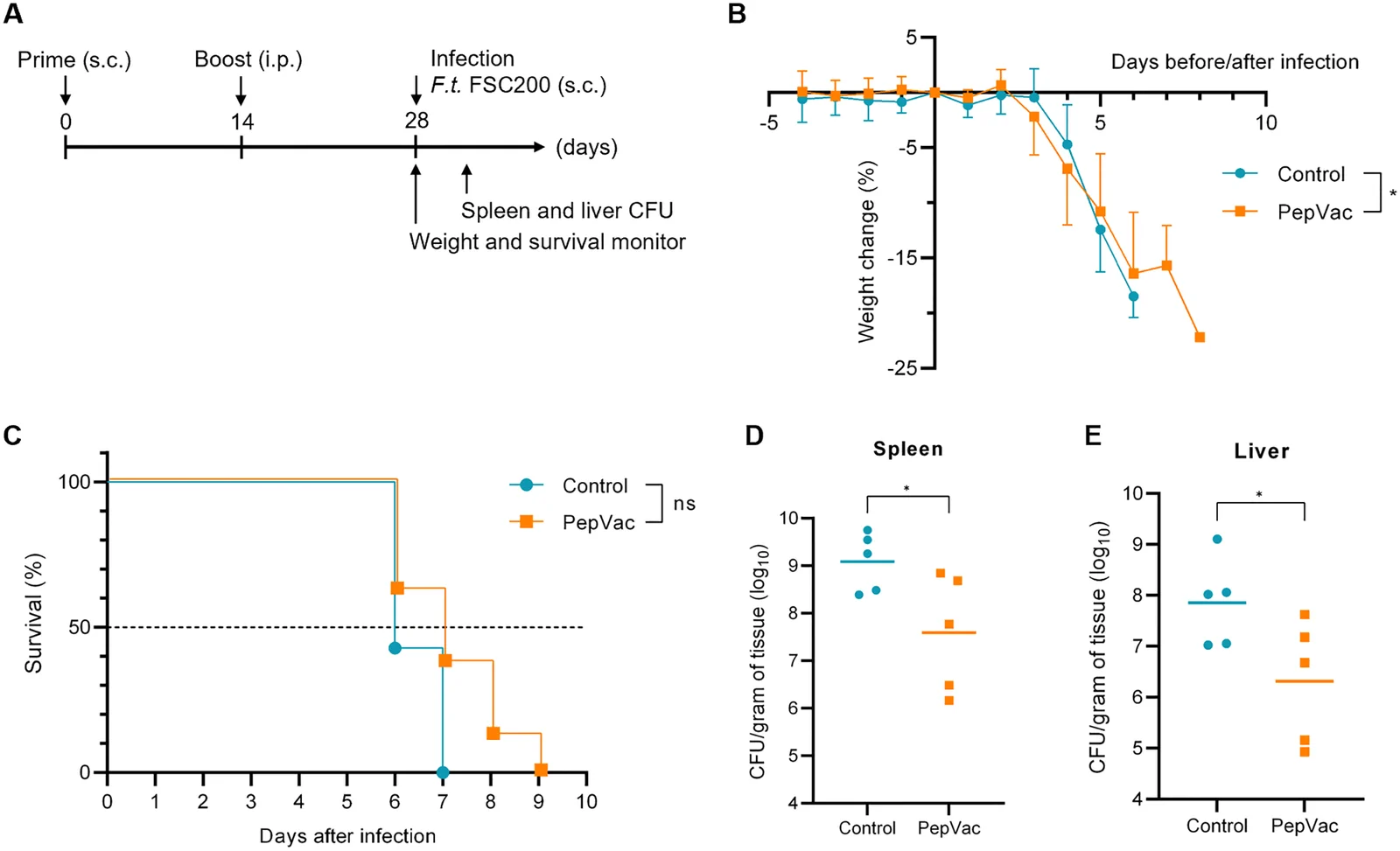
Vaccine-induced immunity is activated after infection but is overcome by *F. tularensis* FSC200. (A) Mice immunized with a peptide vaccine (PepVac) or treated with the same adjuvant but without peptides (control) were subcutaneously infected with 53 CFU of *F. tularensis* FSC200, and the vaccine’s protective efficacy was evaluated. (B) Changes in body weight of mice during infection. The body weight of the mice on the day of infection (day 0) was used as the baseline, and the graph shows the percentage changes relative to that day. Data from the four days prior to infection are also included. Values are presented as mean ± SD (one-sided). The data (days 1-5) were analyzed using a mixed-effects model with repeated measures, and multiple comparisons (PepVac versus control at individual time points) were corrected using Sidak’s post hoc test. An asterisk indicates a p-value < 0.05 for the interaction effect between time and treatment. (C) Survival curve for the vaccinated group (n = 8) and the control group of mice (n = 7). The dashed line indicates the median survival time for each group. The data were analyzed using the Kaplan-Meier method with the log-rank test (ns for p ≥ 0.05). Note: The unequal group sizes were due to the death of one mouse after administration of a booster dose as part of the immunization. The bacterial load in the spleen (D) and liver (E) of control and vaccinated mice (n = 5) was determined on day 4 postinfection. Data for individual mice and the corresponding mean values are shown. A two-tailed t-test was used to assess statistical significance. Asterisks indicate p-values < 0.05. This experiment was performed once.

## Discussion

The induction of T cell-mediated immunity is essential for overcoming infections caused by intracellular bacterial pathogens. This applies to primary infection with *F. tularensis* LVS and is of critical importance in secondary infection with the subsp. *tularensis* strain, where any alterations in T cell subpopulations lead to failure of vaccine-induced immunity [13–15]. On the other hand, the vaccine’s ability to elicit a more potent overall T cell response [17–20], or to induce antigen-specific CD8^+^ or CD4^+^ T cells [14,54], enhances its protective effect. Although the key role of CD4^+^ and CD8^+^ T cells in protection has been clearly defined, the *Francisella* antigens and epitopes recognized by these cells have not yet been well characterized.

The contribution of CD4^+^ T cells to protection against *F. tularensis* infection appears to be just as important as that of CD8^+^ T cells [13,14], despite the cytosolic lifestyle of the bacterium. Our goal was therefore to identify *Francisella* antigens that are recognized by CD4^+^ T cells in a mouse model of tularemia and that could play a protective role. To achieve this, we applied MS-based immunopeptidomics to identify bacterial peptides presented by MHC-II molecules of BMDCs previously infected with *F. tularensis* LVS or the LVSΔdsbA strain. Our analysis yielded 57 unique *Francisella* peptides originating from 24 epitope regions of the 23 different proteins. The characteristics of bacterial peptides, such as sequence length distribution, predicted binding to I-A^b^, and binding motif, were similar to those of endogenous peptides, confirming that they were generated as a result of antigen processing and presentation in BMDCs. Interestingly, the length distribution of bacterial peptides was slightly shifted toward longer peptides compared to endogenous peptides. A similar pattern has been described for HLA-II peptides in activated human dendritic cells and B cells [55]. We hypothesize that longer bacterial peptides may reflect infection-associated activation of BMDCs, whereas shorter endogenous peptides likely originate predominantly from uninfected cells. Although we initially began our experiments with *F. tularensis* LVS, the altered intracellular phenotype of the LVSΔdsbA strain led us to include it in the study, as we hypothesized that it might interact more strongly with the MHC class II antigen processing pathway. Furthermore, it has been shown that the LVS strain suppresses MHC-II surface expression in macrophages [56], though we did not observe this in BMDCs. The use of the LVSΔdsbA strain indeed helped us identify more bacterial MHC-II peptides, and we demonstrated that this cannot be attributed to changes in antigen availability at the proteome level. Bacterial peptides were detected in the MHC-II peptidome of infected BMDCs as rare and low-abundance compounds. The number of BMDCs used for immunoprecipitation was a critical parameter in our experiments for identifying a larger number of immunopeptides. However, improving the sensitivity of the workflow in terms of both sample preparation and LC-MS/MS analysis would allow for a more in-depth characterization of the immunopeptidome and the identification of a broader spectrum of bacterial peptides, which, in our opinion, is not yet complete.

We identified 23 antigens, of which only IglC, DnaK, and FopA1 had previously been described as MHC-II antigens in *F. tularensis*. Based on their functional annotation, the proteins FTL_1678, DacD, and FopA1 were classified under the COG category “cell wall/membrane/envelope biogenesis.” These proteins are therefore accessible not only for presentation on MHC-II molecules and recognition by T cell receptors, but also for interaction with B cell receptors and the induction of an antibody response. Most of the other proteins were associated with functional categories related to translation, nutrient acquisition, and energy production, which are essential for the sustained replication of *Francisella* in the cytosol [7]. We also demonstrated that the identified *Francisella* MHC-II antigens originate from a highly abundant fraction of its proteome. A similar finding has been reported for MHC-II antigens of *M. tuberculosis* [35]. This likely reflects the limited sensitivity of the immunopeptidomic approach, which favors the detection of peptides from more abundant proteins. However, many highly abundant proteins were not detected as MHC-II antigens, confirming that other parameters, such as peptide binding to the MHC-II molecule or the accessibility of the epitope region within the protein structure, also play a role in determining the outcome of antigen processing and presentation [46].

*Francisella* MHC-II peptides were identified using an *in vitro* infection model. However, the spectrum of presented antigens may vary during *in vivo* infection due to changes in the bacterial proteome profile or the type of antigen-presenting cells. We therefore used a mouse model of LVS-induced tularemia to investigate the immunogenicity of the identified peptides. In LVS-immune lymphocytes from the spleen or LNs, IFN-γ-producing cells were induced after stimulation with mixtures of bacterial peptides, and we demonstrated that this response was mediated by CD4^+^ T cells and was specific for immune lymphocytes. Regardless of the type of lymphocytes or time interval examined, a general order of reactivity of PP-1 ≈ PP-2 < PP-3 was observed, indicating that the PP-3 contains a potent stimulatory peptide(s). This was later confirmed, and two highly reactive peptides, FTL_1678-YK-16 (YPSDFGYSNPDSIRAK) and DnaK-AE-16 (ALEEAFAPIAQKAYAE), were found to be responsible for this reactivity. PP-3 also induced significant production of IFN-γ and TNF-α in the culture supernatant of immune splenocytes, suggesting that these antigen-specific T lymphocytes produce key cytokines involved in protection against infection [57]. Using PPs, we demonstrated that the highest number of IFN-γ-producing cells was detected 3 weeks p.i., followed by a gradual decline in the response in the fourth and fifth weeks after immunization. At these time intervals, the onset of the T lymphocyte contraction phase is likely due to limited antigen availability [58]. Indeed, no bacteria were detected in the spleen and LNs as early as 3 weeks after immunization.

Based on further screening, we identified 10 peptides, derived from an equal number of antigens, as immunogenic epitopes of CD4^+^ T cells in *F. tularensis* LVS. The reactivity of these peptides exhibited a similar profile in both splenocytes and CD4^+^ T cells, was specific to LVS-immune lymphocytes, and was primarily mediated by the effector subpopulation of CD4^+^ T cells. We classified the discovered *Francisella* immunogenic epitopes into minor and major epitopes based on the frequency with which they were recognized across immunizations. Similar to the case of VACV CD8^+^ T cell epitopes [53], we observed a positive correlation between the strength of the immune response and the frequency of recognition across immunizations. Thus, only a few immunizations were sufficient to detect the major epitopes that exhibited the highest reactivity or a very stable response in our experiments. Conversely, a greater number of immunizations is required to detect even less significant epitopes, some of which exhibited reactivity only rarely.

Immunization with *F. tularensis* LVS induces a protective immune response against infection with virulent strains of *F. tularensis* [13,59]. The high reactivity and frequency of recognition make the major epitopes and their source antigens interesting targets for evaluating their role in protection. The epitopes of the FTL_1678, DacD, and FTL_1498 antigens thus elicited a response in all immunizations, the DnaK epitope in 90%, and the MinD epitope in 80% of immunizations. At the same time, the FTL_1678 epitope was superior, and the DacD, DnaK, and FTL_1498 epitopes were at least as effective as the LpnA epitope in inducing IFN-γ-producing cells. A comparison with LpnA is appropriate, as it is a well-known antigen for B cells and T cells in both mice [23] and humans [27], and has already been evaluated as a component of subunit vaccines [60–62]. The FTL_1678 (LD-carboxypeptidase) and DacD antigens are involved in peptidoglycan biosynthesis, envelope integrity, and virulence of *F. tularensis* [63–66], making them attractive antigens for immunological intervention from a functional perspective as well. Interestingly, the DacD homolog in *Bordetella pertussis* has been found to be an immunodominant antigen of human CD4^+^ T cells [52]. The functions of the other major antigens are not yet known. FTL_1498 has so far been described as a secreted protein [67], and MinD as a virulence factor [68]. Of the identified minor antigens, only FTL_1042 (peptidyl-prolyl cis-trans isomerase) has been tested as a vaccine component to date [69]. Furthermore, epitopes of the ribosomal proteins RplA, RpsJ, and RplQ elicited a response in 10–20% of immunizations and thus represent subdominant CD4^+^ T cell epitopes in *F. tularensis* LVS. Ribosomal proteins from other bacterial pathogens have previously been identified as CD4^+^ T cell antigens, but their reactivity depended on the specific pathogen [52,70]. Regarding the DnaK, IglC, and FopA1 antigens, which have been evaluated as components of subunit vaccines in several studies [61,62,69,71,72], only the DnaK epitope showed immunogenicity in our study. It is worth noting that the FopA1 peptide elicited an immune response in 20% of immunizations but did not reach statistical significance after correction for multiple comparisons.

The functional potential of the identified peptide epitopes was ultimately evaluated under conditions of *F. tularensis* infection. In these experiments, we successfully immunized mice with a peptide vaccine containing five major peptide epitopes of *F. tularensis* LVS, which elicited a cellular immune response involving IFN-γ, IL-2, and TNF-α. Furthermore, we demonstrated that the induced immune response contributed to early control of the bacterial load and reduced disease severity following infection with the virulent *F. tularensis* FSC200 strain, although this alone was insufficient to provide full protection. From the perspective of immunity mediated by antigen-specific CD4^+^ T cells, we are considering two possible causes of the limited efficacy of vaccine-induced immunity. First, the protective potential of all five epitopes may not be fully exploited during infection. Although these epitopes are conserved in the virulent FSC200 strain, they may not all be effectively presented during infection and therefore remain unrecognized. Second, and this is perhaps the decisive reason, *F. tularensis* FSC200 is highly virulent in mice [6], and protection mediated solely by CD4^+^ T cells may not be sufficient on its own, as has been demonstrated in studies with virulent strains of subsp. *tularensis* [13–15]. It must be acknowledged that mice are highly susceptible to infection with *F. tularensis*. For this reason, Fisher 344 rats are currently considered a more predictive and suitable animal model for evaluating vaccine efficacy [20,73,74]. Although T cell epitopes may differ among MHC alleles, the antigens entering the MHC pathway may be similar across different host species; therefore, their immunogenic and protective potential could be evaluated in a more suitable animal model in future studies.

In summary, using MS-based immunopeptidomics, we identified MHC class II-presented peptides from *F. tularensis* LVS and confirmed that these are immunogenic epitopes for CD4^+^ T cells in a mouse model of tularemia. We demonstrated that CD4^+^ T cell responses were elicited against various bacterial epitopes, thereby revealing the previously unknown breadth and specificity of T cell responses induced by *F. tularensis* LVS. Several epitopes capable of eliciting a strong CD4^+^ T cell response were discovered, and their protective potential in a peptide vaccine was evaluated. The identified epitopes may also enable the development of tools for characterizing antigen-specific CD4^+^ T cells in vaccination studies or T cell biology research.

## Materials and methods

### Ethics statement

All experiments using mice were conducted in accordance with protocols approved by the Ethics Committee for Animal Care and Use at the Military Faculty of Medicine, University of Defence (Czech Republic), under project No. 5/21 (4284/2021-MZE-18134).

### Bacteria

*F. tularensis* subsp. *holarctica* LVS (strain FSC155), subsp. *holarctica* strain FSC200 (both provided by Prof. Forsberg, Sweden), and deletion mutant on *dsbA* gene in the LVS strain [40] (LVSΔdsbA) were grown and harvested within a BioSafety Level 2 containment facility. The bacteria were thawed from a frozen stock and cultured on McLeod agar supplemented with bovine hemoglobin (#212392) and IsoVitaleX (#211876, both from Becton Dickinson) at 37°C for 24 hours. The LVS and LVSΔdsbA strains were subsequently passaged onto a new agar plate and cultured overnight under the same conditions. On the day of infection of mice or BMDCs, the bacteria were resuspended in sterile phosphate-buffered saline (PBS) (#D8537, Sigma-Aldrich) and serially diluted to the desired inoculum dose. The actual number of bacteria was determined by plating serial dilutions and enumerating colony-forming units (CFU). For proteomics, following the initial 24 h culture on McLeod agar, the bacteria were inoculated in Chamberlain medium and grown at 37°C to OD_600_ of 0.7.

### Mice

Specific-pathogen-free 6–8 week-old female C57BL/6J mice (Velaz, CZ) were housed under conventional conditions with *ad libitum* access to food and water and were allowed to acclimatize for at least 14 days before starting experiments. At the indicated time points, animals were euthanized by isoflurane inhalation followed by cervical dislocation.

### Generation and infection of bone marrow-derived dendritic cells (BMDCs)

Femoral and tibial bone marrow cells were isolated from female C57BL/6 mice and BMDCs were generated as previously described [43] with minor modifications. Approximately 2 × 10^7^ bone marrow cells were plated in a 15-cm tissue culture dish containing 20 ml RPMI-1640 medium (#R8758) supplemented with 10% v/v fetal bovine serum (FBS) (#F4135), 50 U/ml penicillin and 50 µg/ml streptomycin (#P0781, all from Sigma-Aldrich). The cells were incubated overnight at 37°C in a humidified atmosphere with 5% CO_2_. Next, non-adherent cells were transferred to a new dish and cultured in RPMI-1640 supplemented with 10% FBS and 5% v/v supernatant from Ag8653 cells transfected with cDNA encoding murine granulocyte-macrophage colony-stimulating factor. Cells were then passaged every 2–3 days and on day 9 of culture the suspension cells were harvested for experiments. The 1 × 10^8^ BMDCs in 25 ml of RPMI-1640 containing 10% FBS were infected with *F. tularensis* LVS or the LVSΔdsbA strain at a multiplicity of infection of 5 (bacteria: cells). Infection was carried out at 37°C in a 50 ml Falcon tube for 30 minutes with gentle shaking. After this, 2 × 10^7^ cells were transferred to a 15-cm Petri dish with an additional 15 ml of RPMI-1640 containing 10% FBS and incubated for 22–24 h under standard conditions. The cells were collected by gentle scraping with a cell scraper, washed with PBS and stored as a dry pellet at -80°C for immunopeptidomics. In some experiments, control cells were treated with 0.5 µg/ml lipopolysaccharide from *E. coli* O55:B5 (#L6529, Sigma-Aldrich).

### Generation of immunoaffinity beads

Monoclonal antibody Y-3P (ATCC HB-183) against mouse I-A^b^ was purified from the supernatant of hybridoma cells (Moravian-Biotechnology, CZ) and used for construction of immunoaffinity beads. The 3 mg of antibody was bound to 1 ml of beads of Protein G Agarose Fast Flow (#MILL16–266, Millipore) by rotating the suspension in Econo-Column (#7371007, Bio-Rad) for 2 h at 4°C. To cross-link the antibody to beads, 20 mM dimethyl pimelimidate (#D8388-1G, Sigma-Aldrich) in 0.2 M triethanolamine pH 8.5 (#90279, Sigma-Aldrich) was used for 1 h at room temperature. The reaction was quenched by incubation with 0.1 M ethanolamine pH 8.2 (#398136, Sigma-Aldrich) for 90 min. Finally, following washing with 0.2 M acetic acid (#5.43808.0100, Merck), the immunoaffinity beads were stored in 50 mM Tris pH 7.6 (#T6066, Sigma-Aldrich), 150 mM NaCl (#73575, Fluka) at 4°C until use. The success of individual steps in the procedure was checked by SDS-PAGE.

### Isolation of MHC-II complexes and associated peptides

The protocol was adopted from previously described procedures [75,76] with modifications. Frozen pellets of 3–9 × 10^7^ BMDCs were briefly thawed, resuspended in 1.5 ml of lysis buffer and rotated at 4°C for 1 h to solubilize. Lysis buffer contained 1% w/v CHAPS (#28300, Pierce), 50 mM Tris pH 7.6, 150 mM NaCl, 0.1 mM iodoacetamide (#I6125, Sigma-Aldrich), 5 mM EDTA (#E5134, Sigma-Aldrich) and protease inhibitors- 5 µg/ml aprotinin, 10 µg/ml leupeptin, 5 µg/ml pepstatin A (#78432, #78435, #78436, Pierce) and 1 mM phenylmethylsulfonyl fluoride (#10837091001, Roche). Cell lysates were then cleared by two rounds of centrifugation at 16,000 g for 15 min at 4°C and further precleared by incubation with 0.1 ml Protein G beads for 1 h. To capture the MHC-II-peptide complexes, the precleared lysates were rotated with 0.1 ml immunoaffinity beads at 4°C overnight. Next day, the immunoaffinity beads were transferred to empty Macro SpinColumn (5 µm frit, #74–3820, Harvard Apparatus) and washed with 0.8 ml 0.1% CHAPS, 150 mM NaCl in 50 mM Tris pH 7.6, with 1.5 ml 450 mM NaCl in 50 mM Tris pH 7.6 and with 1.5 ml 50 mM Tris pH 7.6 buffer. Finally, the beads were eluted with 0.5 ml 10% v/v acetic acid. The eluate was incubated for 10 min at 65°C and the MHC-II peptides were isolated using a two-step solid phase extraction. The eluate was loaded first on 15 mg of equilibrated PolySulfoethyl A resin (#BMSE1203, PolyLC) packed in Macro SpinColumn. The column was then washed sequentially with 15 mM ammonium phosphate pH 2.7 (#09709, Fluka) containing 30% or 15% acetonitrile (ACN) (#1.00030.2500, Merck) and the peptides were released with 0.27 M KCl (#60128, Fluka), 15 mM ammonium phosphate pH 2.7, 15% ACN, while retaining the MHC class II chains on column. Following dilution to 5% ACN with 0.15% trifluoroacetic acid (TFA) (#80457, Sigma-Aldrich), the peptides were loaded on Empore C18-SD 4 mm/1 ml column (#66871-U, Sigma-Aldrich), washed with 5% methanol (#1.06007.2500, Merck), 0.1% TFA and eluted using 0.2 ml of 65% methanol, 0.1% TFA. The dried peptides (Concentrator Plus, Eppendorf) were stored at -20°C until analysis. In some experiments, immunoaffinity purification was monitored by SDS-PAGE with immunodetection using anti-I-A^k^ β-chain rabbit serum (generous gift from Paul A. Roche, NIH, Bethesda, MD) as a primary detection antibody.

### LC-MS/MS analysis

Samples were analyzed using an Ultimate 3000 RSLCnano system interfaced to a Q Exactive mass spectrometer via a Nanospray Flex ion source (all instruments from Thermo Fisher Scientific). For MHC-II peptidomics, peptides were dissolved in 2% ACN, 0.1% TFA and 30–50% of the sample volume was loaded onto PepMap100 C18, 3 µm, 0.075 × 20-mm trap column (#164535, Thermo Scientific). The peptides were separated at 0.25 µl/min and 40°C on a PepMap RSLC C18, 2 µm, 0.075 × 250-mm column (#164536, Thermo Scientific) using conditions as follow: 4% B for initial 7 min, 4–32% B in 93 min, 32–55% B in 23 min, wash with 90% B for 10 min and re-equilibration at 4% B for 21 min, with 0.1% FA (#56302, Fluka) in water as phase A and 80% ACN, 0.1% FA as phase B. Positive ion full scan MS spectra from 350-1,200 *m/z* range were acquired on a 1 × 10^6^ target ion population in the Orbitrap at a resolution of 70,000. Precursor ions with charge state range 2–6, intensity threshold of 3 × 10^4^ and not fragmented during previous 30 s were admitted for higher-energy collisional dissociation. Tandem mass spectra were acquired at resolution of 35,000 and with other parameters set to: 1 × 10^5^ for AGC target, 170 ms for maximum ion time, 1.6 *m/z* for quadrupole isolation window, 27 for normalized collision energy, and *m/z* 140 for fixed first mass. For proteomics, approx. 0.3 µg of peptides were loaded onto Symmetry C18, 5 µm, 0.18 × 20-mm trap column (#186008821, Waters) and separated at 0.25 µl/min and 45°C on a Peptide BEH C18, 1.7 µm, 0.075 × 250-mm column (#186008795, Waters) using conditions as follow: 6% B for initial 7 min, 6–42% B in 90 min, 42–60% B in 18 min, wash with 90% B for 15 min and re-equilibration at 6% B for 26 min. Full scan MS spectra from 350-1,650 *m/z* range with a resolution of 70,000 were acquired in the Orbitrap and precursor ions with charge state range 2–5 and not fragmented during previous 25 s were admitted for HCD. Tandem mass spectra were acquired at resolution of 17,500 and with other parameters set to: 1 × 10^5^ for AGC target, 100 ms for maximum ion time, 1.6 *m/z* for quadrupole isolation window and 27 for normalized collision energy.

### Sample preparation for proteomic analysis

LVS and LVSΔdsbA bacteria, grown in quadruplicate, were harvested from Chamberlain culture medium at an OD_600_ of 0.7 by centrifugation and processed as previously described [77] with minor modifications. The pellets were washed, dissolved in 2% w/v sodium deoxycholate (SDC) (#D5670, Sigma-Aldrich), and lysed at 70°C for 5 min. After incubation with 0.1 unit/ml of benzonase endonuclease (#E1014, Millipore) at room temperature for 30 min to hydrolyze nucleic acids, the lysates were clarified by centrifugation for 10 min at 14,000 g. Total protein was determined by bicinchoninic acid assay (#BCA1, Sigma-Aldrich). An aliquot of 50 µg was dissolved in 2% SDC, 50 mM ammonium bicarbonate (#PN09830, Sigma-Aldrich) and the proteins were reduced with 4 mM dithiothreitol (#D5545, Sigma-Aldrich) at 60°C for 30 min and alkylated with 12 mM iodoacetamide (#I6125, Sigma-Aldrich) for 30 min at room temperature in the dark. The excess of iodoacetamide was quenched by addition of dithiothreitol, the concentration of SDC adjusted to 0.5% with 50 mM ammonium bicarbonate and 1 µg of rLys-C and 1 µg of trypsin (#V167A and #V511A, Promega) were added for overnight digestion at 37°C. After digestion, the mixtures were acidified with hydrochloric acid and the precipitated deoxycholic acid was removed by extraction into ethyl-acetate (#34858, Sigma-Aldrich). The samples were then cleaned on Empore C18-SD column and eluted peptides were dried and stored at -20°C until analysis.

### Peptides identification

Peptides and proteins were identified using the Proteome Discoverer v2.4 (Thermo Fisher Scientific) with the integrated search tool Mascot v2.4.1 (Matrix Science). Mass spectra from immunopeptidomic analyses were searched using a precursor mass tolerance of 20 ppm, a fragment mass tolerance of 0.02 Da, methionine oxidation as a dynamic modification, and protein digestion without enzyme restriction. Tandem mass spectra were deisotopized (IMP-MS2 spectrum processor) before searching to reduce their complexity. The database contained sequences from *Mus musculus* (55,021 entries, Uniprot), *F. tularensis* subsp. *holarctica* strain LVS (1,678 entries, Uniprot), and general contaminants (256 entries). Detection, alignment, and matching of peptide features across samples were enabled with default settings. Searches were performed against target and decoy databases and re-evaluated with Percolator v3.02.1 [78]. Identifications at the peptide-spectrum match level, peptide group level, and protein level meeting a percolator q-value threshold of 0.01 were considered for further analysis. In addition to the q-value, peptide ion scores and XICs in infected and uninfected control samples were also considered in the manual validation of bacterial peptide identification. The identified contaminants and class II peptides from serum proteins of the culture medium were filtered out from the data. For proteomics, a precursor mass tolerance of 20 ppm, a fragment mass tolerance of 0.025 Da, trypsin cleavage specificity (unlimited by proline), and up to two missed cleavage sites were used to search mass spectra in the sequence database (*F. tularensis* LVS appended with general contaminants). Oxidation (M), deamidation (NQ), carbamidomethyl (N-terminus), Gln to pyro-Glu (N-term Q), and carbamidomethyl (C) were used as dynamic and static modifications. Identifications were controlled at an FDR of 0.01. For comparative analysis of LVS and LVSΔdsbA strains, samples were normalized to total peptide amount and the obtained protein ratios were evaluated for differential expression using a background-based t-test with FDR control at 0.05. Quantification of at least two peptides and a 20% change were used as additional criteria. To estimate the relative abundance of proteins in the proteome, the protein intensity used for scoring was calculated as the average intensity of the three most abundant peptides for a given protein [79] and its median from four replicates was used.

### Bioinformatics

To verify the identified bacterial peptide sequences, spectral similarity analysis was performed using the Universal Spectrum Explorer tool [80]. A mass tolerance of 15 ppm was used for the annotation of b- and y-fragments and for peak matching. To characterize the MHC-II peptidome, the identified peptides (unique sequences) were analyzed for several properties. Predictions of peptide binding to the I-A^b^ allele were performed using the NetMHCIIpan-4.3 [81] program with default settings and eluted ligand prediction mode. GibbsCluster-2.0 [82] was used to generate the binding motif for peptides with one cluster, or 1–2 clusters allowed for bacterial and mouse peptides, respectively. The sequence motif length was 9, trash cluster was enabled, and other parameters were left at default values. Peptides of *F. tularensis* LVS were also evaluated for sequence conservation in different *Francisella* strains using BLASTP [83] in the Kyoto Encyclopedia of Genes and Genomes (KEGG) [84] database and for their homology to mouse peptides using the PEPMatch tool [85] hosted on the Immune Epitope Database (IEDB). For functional annotation of *F. tularensis* LVS proteins, the FTL gene names were converted to the corresponding FTT gene names in *F. tularensis* subsp. *tularensis* SCHU S4 before submitting them to the Clusters of Orthologous Genes (COG) [49] and KEGG databases. The proteomics data were deposited to the ProteomeXchange Consortium via the PRIDE partner repository [86].

### Synthetic peptides

Peptides were commercially synthesized with a purity greater than 90% and supplied as lyophilized TFA salts (ProImmune, UK). Peptides were dissolved in sterile PBS to obtain theoretical 1 mM stock solutions and stored at -40°C. The actual concentration of peptides was determined from their peak areas after LC-UV analysis in combination with sequence-specific extinction coefficients [87]. For this purpose, the peptides were analyzed on a Poroshell 120 SB-C18 column, 2.1 × 100 mm (#685775–902, Agilent) using a mobile phase of water-ACN-0.1% TFA and monitored at 205 nm (Alliance 2695, Waters). The peptide Angiotensin I (#A9650, Sigma-Aldrich) served as a standard, the concentration of which was determined from the absorbance at 280 nm and ε 1290 M^-1 cm-1^ of tyrosine using a NanoPhotometer (Implen). Then, working solutions of the peptides were prepared with a concentration of 0.2 mM and stored in aliquots at -40°C. Synthetic peptides were analyzed by LC-MS/MS using Q Exactive to confirm their sequences and obtain reference spectra for spectral similarity analysis.

### Immunization of mice

Mice aged 8–11 weeks, age-matched in the experiment, were infected subcutaneously in the flank with 3 × 10^4^ CFU of *F. tularensis* LVS diluted in 0.2 ml PBS. Spleens and lymph nodes were harvested 3–5 weeks later, and lymphocytes were prepared for screening of T cell reactivity. Control mice received sterile PBS alone.

To evaluate the immunogenicity of T cell epitopes, a peptide vaccine containing five *Francisella* epitopes (FTL_1498-KE-15, MinD-IR-16, FTL_1678-YK-16, DnaK-AE-16, DacD-RK-17) was formulated and immunization was performed according to a previously described procedure [88]. Mice aged 13–16 weeks were primed with 25 µg of each peptide emulsified in Complete Freund’s Adjuvant (CFA), which consisted of Incomplete Freund’s Adjuvant (#F5506, Sigma-Aldrich) supplemented with 5 mg/ml of heat-inactivated *Mycobacterium tuberculosis* H37Ra (#231141, BD Difco). The emulsion was prepared by mixing equal volumes of CFA and peptides dissolved in PBS using two glass syringes connected by a capillary. The emulsion (total 0.2 ml) was administered subcutaneously into two dorsal injection sites per mouse. A booster injection (0.2 ml) was delivered intraperitoneally 14 days later, containing 25 µg of each peptide, 50 µg of high molecular weight poly(I:C) (#tlrl-pic, InvivoGen), and 100 µg of anti-CD40 antibody (clone FGK4.5/FGK45, #BE0016–2, BioXcell). Control mice received the same formulations but without peptides.

### Protective study

The vaccinated and control mice were treated as described above. Fourteen days after the booster injection, mice were given a lethal dose of 53 CFU of *F. tularensis* subsp. *holarctica* strain FSC200 (in 0.2 ml PBS) subcutaneously in the flank and monitored for weight loss and survival. To determine bacterial burden, spleens and livers were aseptically isolated 4 days after infection and homogenized through a 70 µm mesh filter (#93070, SPL Life Sciences) in 2 ml PBS for spleen and 4 ml PBS for liver to form single cell suspensions. The suspensions were then serially diluted and 50 µl of each dilution was plated in triplicate on McLeod agar plates supplemented with penicillin (100 units/ml). Colonies were counted after one week of incubation at 37°C and bacterial load was expressed as CFU per gram of organ.

### Preparation of lymphocytes

Spleens were aseptically removed from mice and homogenized through a 40 µm mesh filter (#93040, SPL Life Sciences) in 2 ml of PBS per spleen. Cells were then pooled from 3-4 mice (LVS immunization experiments) or left separate (peptide immunization experiments), and erythrocytes were lysed with ACK Lysing Buffer (#A1049201, Gibco). Following filtration through a 40 µm filter, the single-cell suspensions of splenocytes were either used in ELISpot, ELISA, and FACS assays or subjected to CD4^+^ T cell purification. This was performed by negative selection via magnetic column using a CD4^+^ T Cell Isolation Kit (#130-104-454, Miltenyi Biotec) according to manufacturer´s instructions. In some experiments, lymph nodes (mandibular, axillary and subiliac) were also isolated and processed as above, except for ACK treatment, and lymphocytes were used in ELISpot and FACS assays directly or after enrichment of CD4^+^ T cells. Purified B cells, which were used in cocultures, were obtained from splenocytes suspension (no ACK-treatment) of syngeneic age-matched naïve mice by negative selection separation using the EasySep Mouse B Cell Isolation Kit (#19854, Stemcell Technologies) following manufacturer´s instructions. The purity of isolated cell populations was verified by flow cytometry and the viability of lymphocytes entering the subsequent assays was > 90%.

### ELISpot

IFN-γ production of peptide-stimulated lymphocytes was detected using an ELISpot Plus-HRP (#3321–4HPW-10) or ELISpot Flex-HRP (#3321-2H, both from Mabtech) in conjunction with MultiScreenHTS IP Filter Plate (#MSIPS4W10, Millipore) according to manufacturer’s instructions. For the assay, lymphocytes were resuspended in RPMI-1640 supplemented with 10% FBS and penicillin/streptomycin (50 U/ml and 50 µg/ml, respectively). For stimulation of whole-cell suspensions from spleens and lymph nodes, 5 × 10^5^ cells in 200 µl were plated per well and 5 µl of peptide pools (Table B in S1 Appendix) or 10 µl of individual peptides were added to reach a final concentration of 5 or 10 µM, respectively. The cells were then cultured under stable conditions at 37°C in 5% CO_2_ for 20–22 h. After washing according to the manufacturer’s instructions, the spots were developed by adding the HRP substrate tetramethylbenzidine (#3651-10, Mabtech), after which the plates were washed with deionized water and allowed to air-dry. Cytokine secretion in mice immunized with the peptide vaccine was determined similarly except that due to the unknown magnitude of the response, two different cell numbers were plated, ranging from 1-5 × 10^5^ cells. IL-2 and TNF-α secretion was also determined from these lymphocytes (ELISpot Flex #3441-2H and #3511-2H, Mabtech). For coculture-based assays, 2 × 10^5^ purified B cells in a volume of 100 µl were plated per well and allowed to settle down at the bottom of the well for 30 min in an incubator. Then, peptides were added as described above and immediately thereafter a 3 × 10^5^ purified CD4^+^ T cells in 100 µl was carefully added. In initial experiments, BMDCs at seeding density of 3 × 10^4^ cells/well were used in cocultures as antigen presenting cells instead of B cells. Subsequent cultivation conditions, plate washing and spot-development steps were the same as described above for whole-cell suspensions. In an assay, four wells were usually used per peptide and 4–6 wells for unstimulated control cells. In addition, peptide RLQWQAPEGSKCHD of *Francisella* LpnA (FTL_0421) was used as a positive peptide [23]. Tryptic peptide YAIAVNDLGTEYVHR of mouse Glutaminase (Gls, acc. D3Z7P3) which was predicted as I-A^b^ non-binder or Ovalbumin (323–339) peptide (#O1641, Sigma-Aldrich) was used as a negative peptide. Finally, the wells containing 4 µg/ml of Leucoagglutinin (PHA-L) (#L4144, Sigma-Aldrich) served as technical controls in the assays. Plates were scanned and the number of spot-forming cells was determined using an ImmunoSpot S6 Analyzer (Cellular Technology Limited) (assays in Fig 3A-3D, and Fig D in S1 Appendix) or ASTOR 2 (Mabtech) (assays in Figs 4A-4D, 5A, 6B-6E, and Figs E, F, H, I in S1 Appendix).

### FACS analysis and intracellular IFN-γ staining

To measure intracellular IFN-γ production, 3 × 10^4^ BMDCs or 2 × 10^5^ isolated B cells (preparation described above) were supplemented with 5 µM final concentration of peptide pools and cocultured with 3 × 10^5^ CD4^+^ T cells in a 5% CO_2_ incubator at 37°C for 24 h. CD4^+^ T cells were isolated from LNs or spleens of control or immunized mice. Brefeldin A (#B7450, Thermo Fisher Scientific) was added to a final concentration of 5 µg/ml for the last 4 h of incubation. Cells were then stained for the desired surface markers and fixed according to the manufacturer's protocol using an Intracellular Fixation and Permeabilization Buffer Set (#88-8824-00, eBioscience) followed by staining with anti-IFN-γ antibody. Measurement was performed using a BD FACSymphony A5 Cell Analyzer and analysis was performed with FlowJo software (Becton, Dickinson and Company). Antibodies against CD4 (#100453), CD44 (#103032), TCRβ (#109220), B220 (#103227), MHC-II (#116507), IFN-γ (#505810, all from Biolegend), CD62L (#741230, BD biosciences) and fixative cell viability Dye 506 (#65-0866-18, Thermo Fisher Scientific) were used.

### ELISA

Single-cell suspensions of splenocytes were plated in clear flat-bottom 96-well MaxiSorp plates (#442404, Thermo Fisher Scientific) at amount of 8 × 10^5^ cells per well, stimulated with 5 µM peptide pools and cultured at 37°C in 5% CO_2_ for 48 h. The concentrations of IFN-γ and TNF-α in supernatants were quantified using Mouse DuoSet ELISA (#DY485–05 and #DY410–05, Bio-Techne, R&D Systems) with absorbance reading at 450 nm using a Synergy H1 Reader (BioTek Instruments).

### Statistical analysis

Data visualization and analyses were performed in Prism v9.5.1. (GraphPad Software). The types of tests used to assess statistical significance of the data are described in the figure legends. For ELISpot assays of individual biological experiments, SFCs of peptide- or PPs-stimulated cells were compared with unstimulated cells (background) using one-way ANOVA followed by multiple testing correction. The net average peptide reactivity was obtained by subtracting the average background reactivity. The number of SFCs in peptide-immune lymphocytes was normalized to 1.5 × 10^5^ cells.

## Supporting information

S1 File Minimal data set. (XLSX)

S1 Appendix Supporting results. (PDF)

S1 Table Identified MHC-II peptides from *Francisella*-infected BMDCs. (XLSX)

S2 Table Analyses and annotations of *Francisella* MHC-II peptides. (XLSX)

S3 Table Results of LVS and LVSΔdsbA proteome analysis. (XLSX)
