# Supplementary material for: Immunopeptidomics reveals conserved and abundant Francisella tularensis proteins as immunogenic CD4 T cell antigens in mice
Source: PLoS Pathog. 2026 Sep 29;22(9):e1014597. doi: 10.1371/journal.ppat.1014597 (PMC13649117; doi:10.1371/journal.ppat.1014597)
Supplement: Supplementary file 1 [file ppat.1014597.s002.pdf]

Supporting Information

Immunopeptidomics reveals conserved and abundant *Francisella tularensis* proteins as immunogenic CD4 T cell antigens in mice

Pavlina Laskova[1], Stanislava Porkertova[1], Ondrej Ballek[2], Lucie Balonova[1], Jana Pavloskova[1], Ales Neuwirth[3], Ivona Pavkova[1], Ivo Fabrik[4], Pavol Lukac[5,6], Luca Vannucci[5], Dominik Filipp[2], Jiri Stulik[1]*, Marek Link[1]*

[1] Department of Molecular Pathology and Biology, Military Faculty of Medicine, University of Defence, Hradec Kralove, Czech Republic

[2] Laboratory of Immunobiology, Institute of Molecular Genetics of the Czech Academy of Sciences, Prague, Czech Republic

[3] Laboratory of Adaptive Immunity, Institute of Molecular Genetics of the Czech Academy of Sciences, Prague, Czech Republic

[4] Biomedical Research Center, University Hospital Hradec Kralove, Hradec Kralove, Czech Republic

[5] Laboratory of Immunotherapy, Institute of Microbiology of the Czech Academy of Sciences, Prague, Czech Republic

[6] Department of Cell Biology, Faculty of Science, Charles University, Prague, Czech Republic

* Corresponding authors

E-mail: jiri.stulik@unob.cz (JS)

E-mail: marek.link@unob.cz (ML)

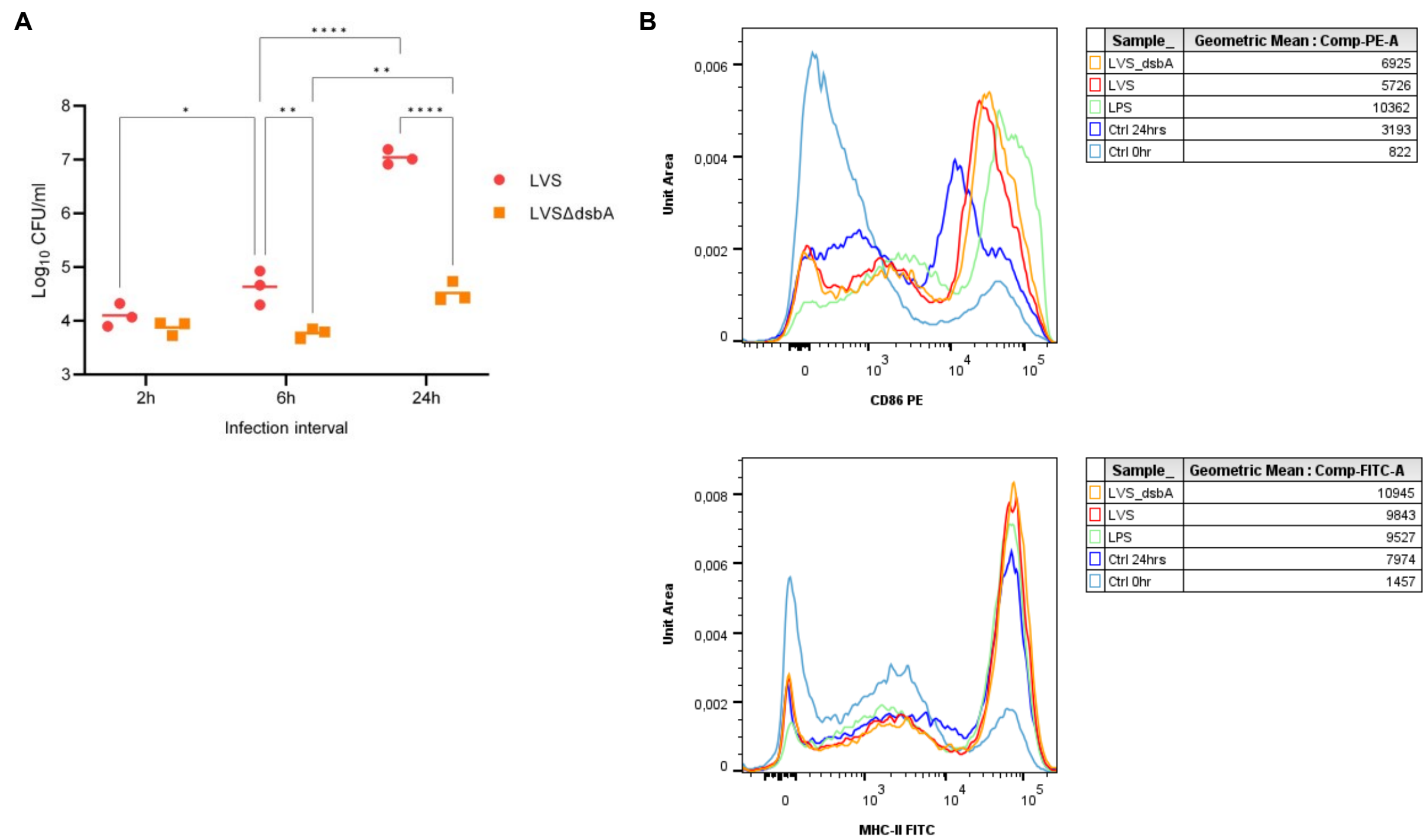

Fig A. Infection of BMDCs with *F. tularensis*. (A) Proliferation of LVS and its *dsbA* gene mutant strain (LVSΔdsbA) in murine BMDCs. BMDCs were infected at a multiplicity of infection of 20 (bacteria:cells) for 1 h and then treated with 5 µg/ml gentamicin for 30 min to kill extracellular bacteria. After washing out the gentamicin, the infection was allowed to continue and BMDCs were harvested and lysed after 2, 6, and 24 h for plating and enumeration of bacterial colony-forming units (CFU). Data from three biological replicates are shown with a line indicating the mean value. The growth of LVSΔdsbA was significantly lower than that of LVS at 6 and 24 h after infection (two-way ANOVA followed by Tukey's post hoc test). Asterisks indicate p-values (*p< 0.05, **p< 0.01, ****p< 0.0001). (B) Analysis of the expression of BMDCs surface markers CD86 (upper panel) and MHC-II (lower panel) by FACS. BMDCs were either infected with LVS or LVSΔdsbA at a multiplicity of infection of 20, or stimulated with 0.5 µg/ml lipopolysaccharide from *E. coli* O55:B5 (LPS), or left untreated (Ctrl) for 24 h and subsequently analyzed by FACS after labelling with anti-CD86 GL1 (#553692, BD Pharmingen) or anti-MHC class II M5/114.15.2 (#11-5321-82, Invitrogen) antibodies. Results for BMDCs entering the experiment (Ctrl 0hr) are also shown. Data are representative results of at least 2 independent experiments.

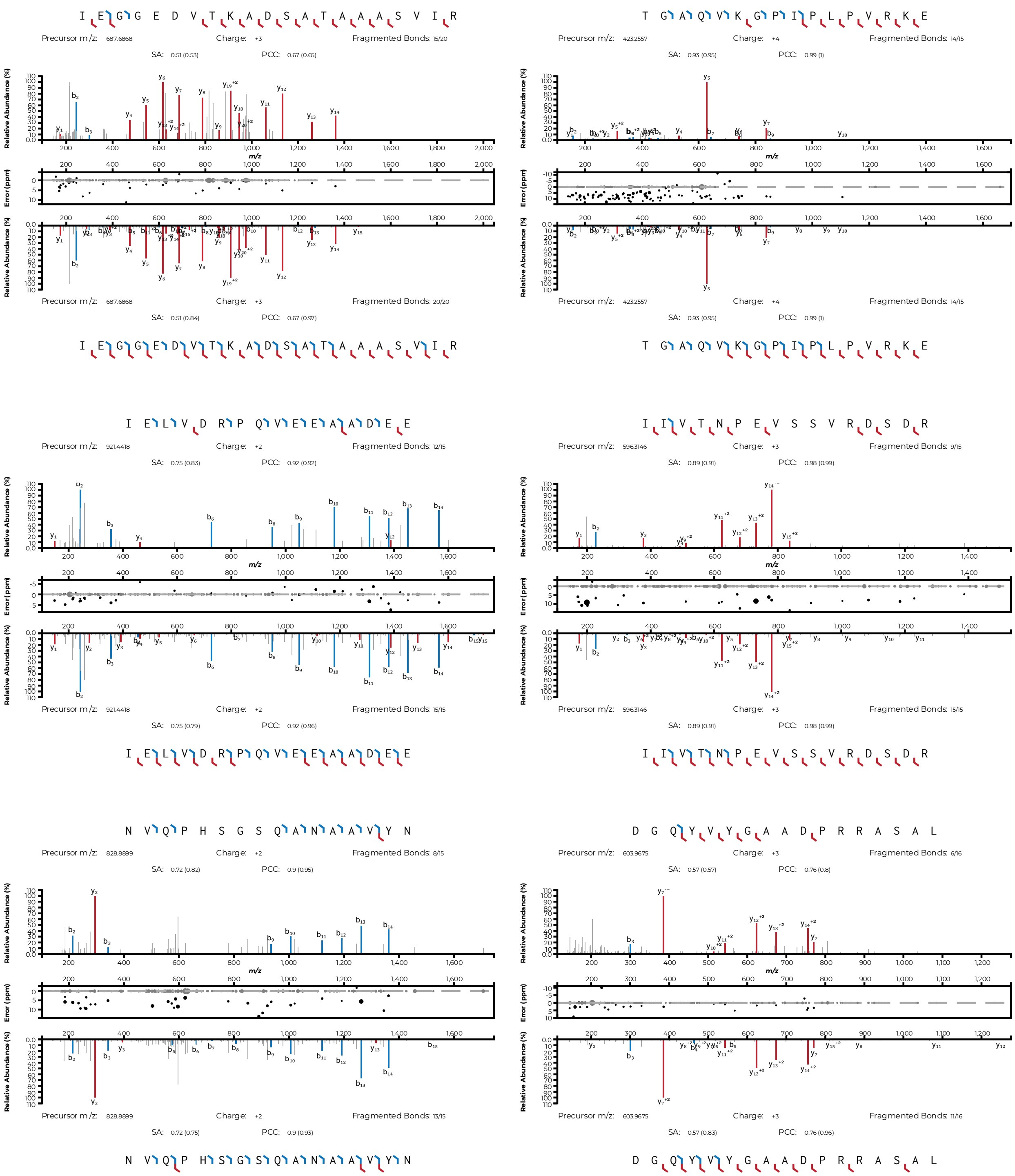
I E G G E D V T K A D S A T A A A S V I R
Precursor m/z: 687.6868
Charge: +3
Fragmented Bonds: 15/20
SA: 0.51 (0.53)
PCC: 0.67 (0.65)
Relative Abundance (%)
m/z
Error (ppm)
Precursor m/z: 687.6868
Charge: +3
Fragmented Bonds: 20/20
SA: 0.51 (0.84)
PCC: 0.67 (0.97)
I E G G E D V T K A D S A T A A A S V I R
T G A Q V K G P I P L P V R K E
Precursor m/z: 423.2557
Charge: +4
Fragmented Bonds: 14/15
SA: 0.93 (0.95)
PCC: 0.99 (1)
Precursor m/z: 423.2557
Charge: +4
Fragmented Bonds: 14/15
SA: 0.93 (0.95)
PCC: 0.99 (1)
T G A Q V K G P I P L P V R K E
I E L V D R P Q V E E A A D E E
Precursor m/z: 921.4418
Charge: +2
Fragmented Bonds: 12/15
SA: 0.75 (0.83)
PCC: 0.92 (0.92)
Precursor m/z: 921.4418
Charge: +2
Fragmented Bonds: 15/15
SA: 0.75 (0.79)
PCC: 0.92 (0.96)
I E L V D R P Q V E E A A D E E
I I V T N P E V S S V R D S D R
Precursor m/z: 596.3146
Charge: +3
Fragmented Bonds: 9/15
SA: 0.89 (0.91)
PCC: 0.98 (0.99)
Precursor m/z: 596.3146
Charge: +3
Fragmented Bonds: 15/15
SA: 0.89 (0.91)
PCC: 0.98 (0.99)
I I V T N P E V S S V R D S D R
N V Q P H S G S Q A N A A V Y N
Precursor m/z: 828.8899
Charge: +2
Fragmented Bonds: 8/15
SA: 0.72 (0.82)
PCC: 0.9 (0.95)
Precursor m/z: 828.8899
Charge: +2
Fragmented Bonds: 13/15
SA: 0.72 (0.75)
PCC: 0.9 (0.93)
N V Q P H S G S Q A N A A V Y N
D G Q Y V Y G A A D P R R A S A L
Precursor m/z: 603.9675
Charge: +3
Fragmented Bonds: 6/16
SA: 0.57 (0.57)
PCC: 0.76 (0.8)
Precursor m/z: 603.9675
Charge: +3
Fragmented Bonds: 11/16
SA: 0.57 (0.83)
PCC: 0.76 (0.96)
D G Q Y V Y G A A D P R R A S A L

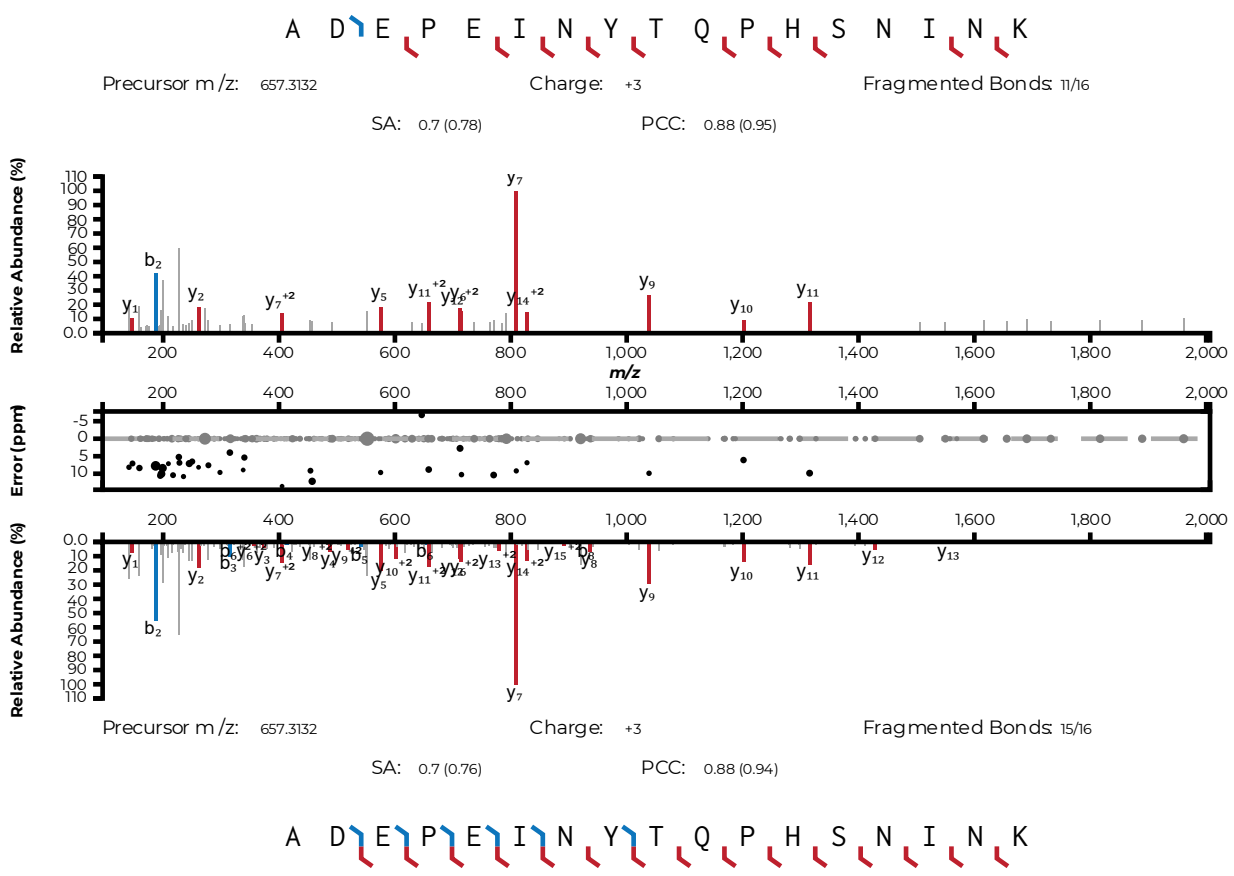

A D E P E I N Y T Q P H S N I N K
Precursor m/z: 657.3132
Charge: +3
Fragmented Bonds: 11/16
SA: 0.7 (0.78)
PCC: 0.88 (0.95)
Relative Abundance (%)
m/z
Error (ppm)
Relative Abundance (%)
Precursor m/z: 657.3132
Charge: +3
Fragmented Bonds: 15/16
SA: 0.7 (0.76)
PCC: 0.88 (0.94)
A D E P E I N Y T Q P H S N I N K

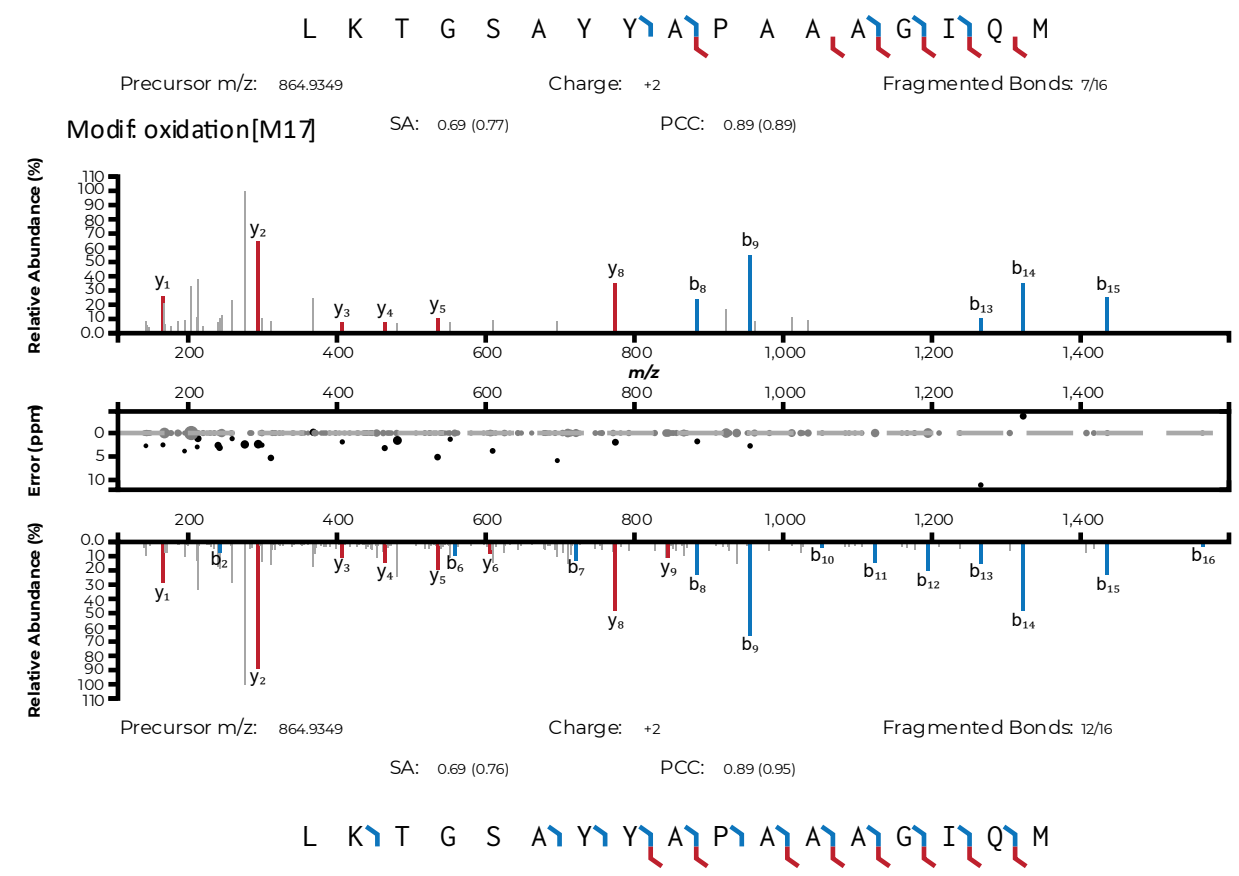

L K T G S A Y Y A P A A A G I Q M
Precursor m/z: 864.9349
Charge: +2
Fragmented Bonds: 7/16
Modif: oxidation[M17]
SA: 0.69 (0.77)
PCC: 0.89 (0.89)
Relative Abundance (%)
m/z
Error (ppm)
Relative Abundance (%)
Precursor m/z: 864.9349
Charge: +2
Fragmented Bonds: 12/16
SA: 0.69 (0.76)
PCC: 0.89 (0.95)
L K T G S A Y Y A P A A A G I Q M

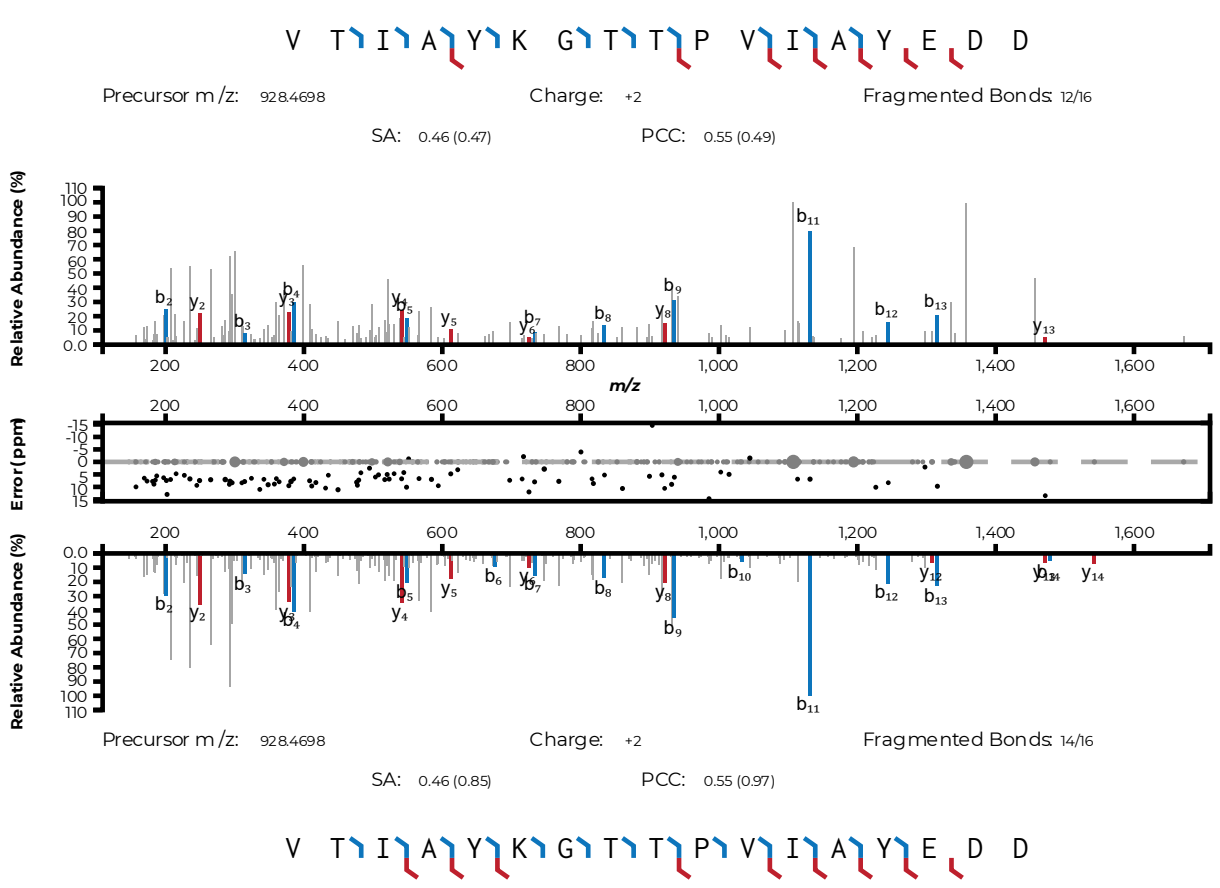

V T I A Y K G T T P V I A Y E D D
Precursor m/z: 928.4698
Charge: +2
Fragmented Bonds: 12/16
SA: 0.46 (0.47)
PCC: 0.55 (0.49)
Relative Abundance (%)
m/z
Error (ppm)
Relative Abundance (%)
Precursor m/z: 928.4698
Charge: +2
Fragmented Bonds: 14/16
SA: 0.46 (0.85)
PCC: 0.55 (0.97)
V T I A Y K G T T P V I A Y E D D

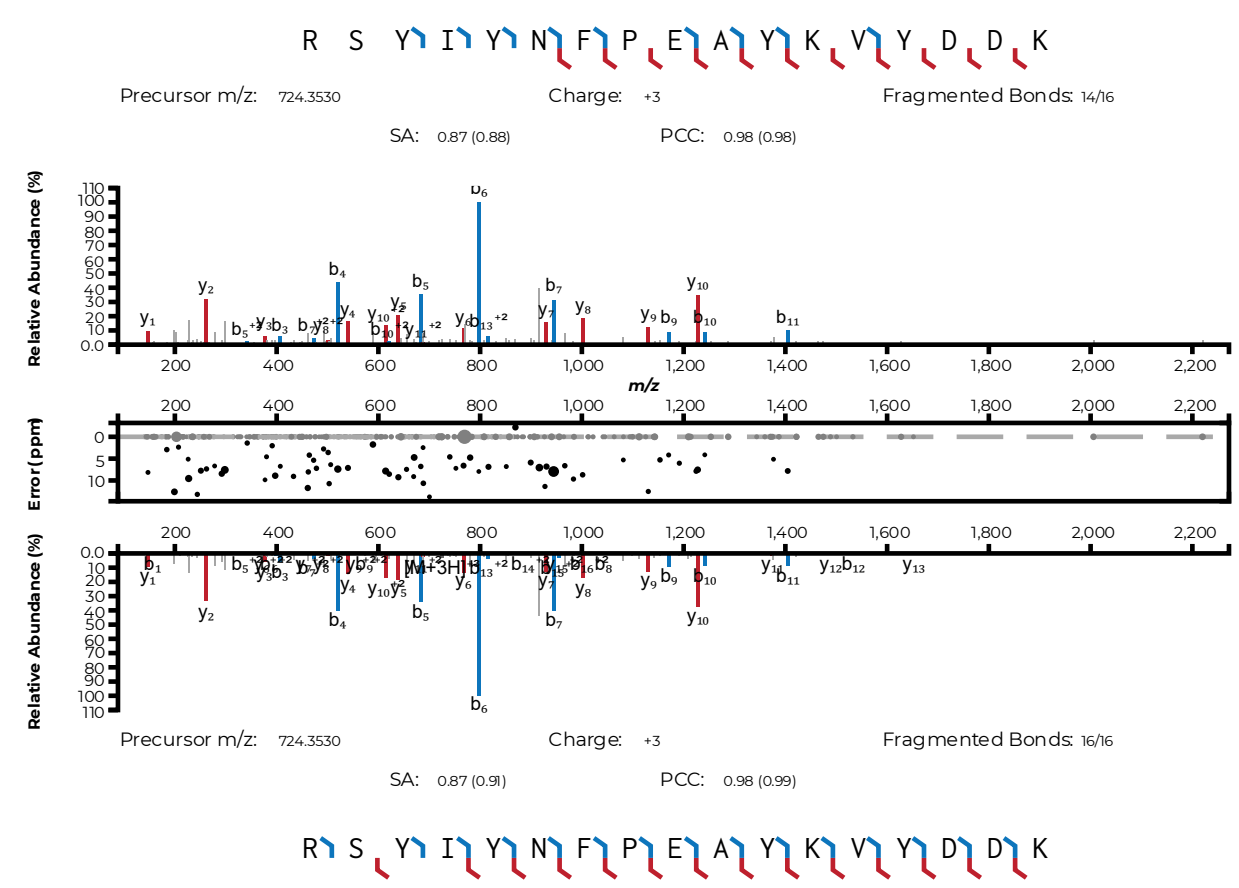

R S Y I Y N F P E A Y K V Y D D K
Precursor m/z: 724.3530
Charge: +3
Fragmented Bonds: 14/16
SA: 0.87 (0.88)
PCC: 0.98 (0.98)
Relative Abundance (%)
m/z
Error (ppm)
Relative Abundance (%)
Precursor m/z: 724.3530
Charge: +3
Fragmented Bonds: 16/16
SA: 0.87 (0.91)
PCC: 0.98 (0.99)
R S Y I Y N F P E A Y K V Y D D K

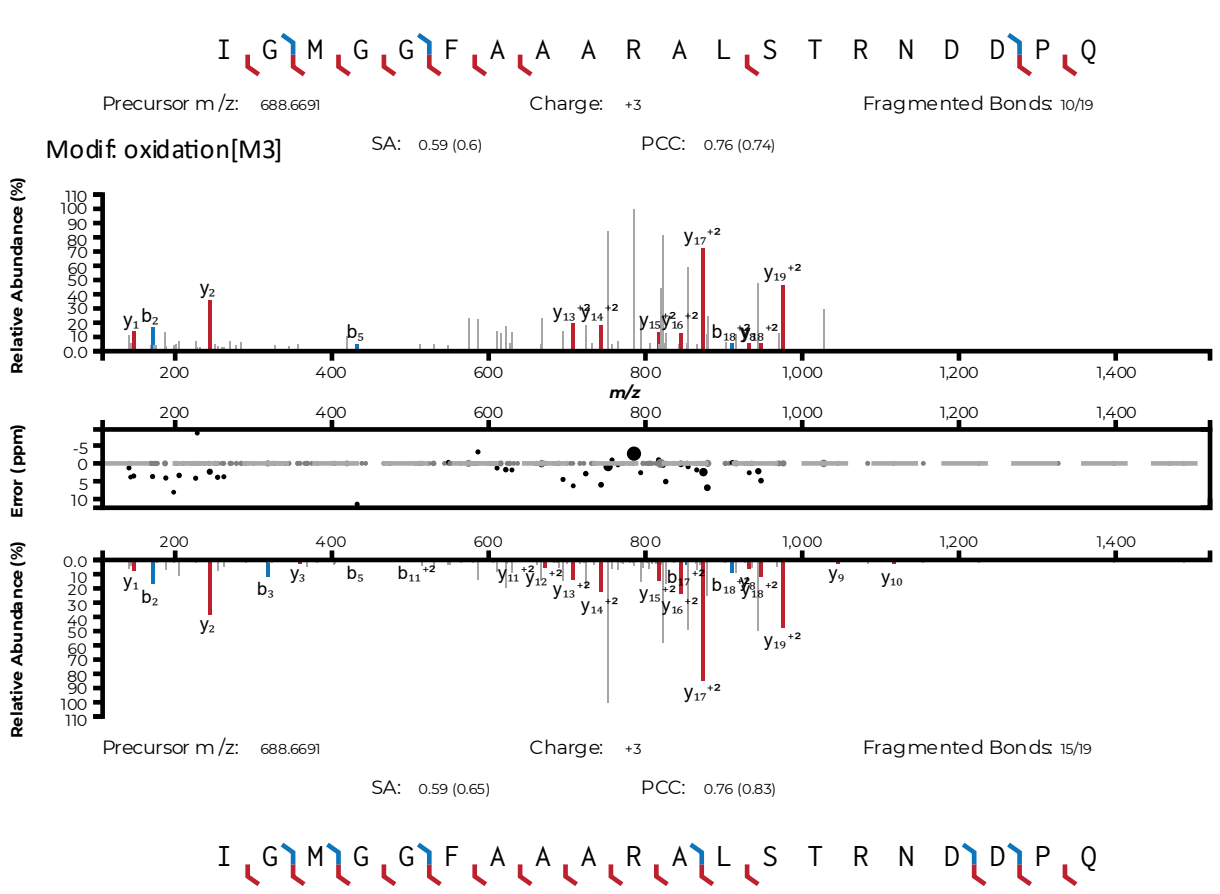

I G M G G F A A A R A L S T R N D D P Q
Precursor m/z: 688.6691
Charge: +3
Fragmented Bonds: 10/19
Modif: oxidation[M3]
SA: 0.59 (0.6)
PCC: 0.76 (0.74)
Relative Abundance (%)
m/z
Error (ppm)
Relative Abundance (%)
Precursor m/z: 688.6691
Charge: +3
Fragmented Bonds: 15/19
SA: 0.59 (0.65)
PCC: 0.76 (0.83)
I G M G G F A A A R A L S T R N D D P Q

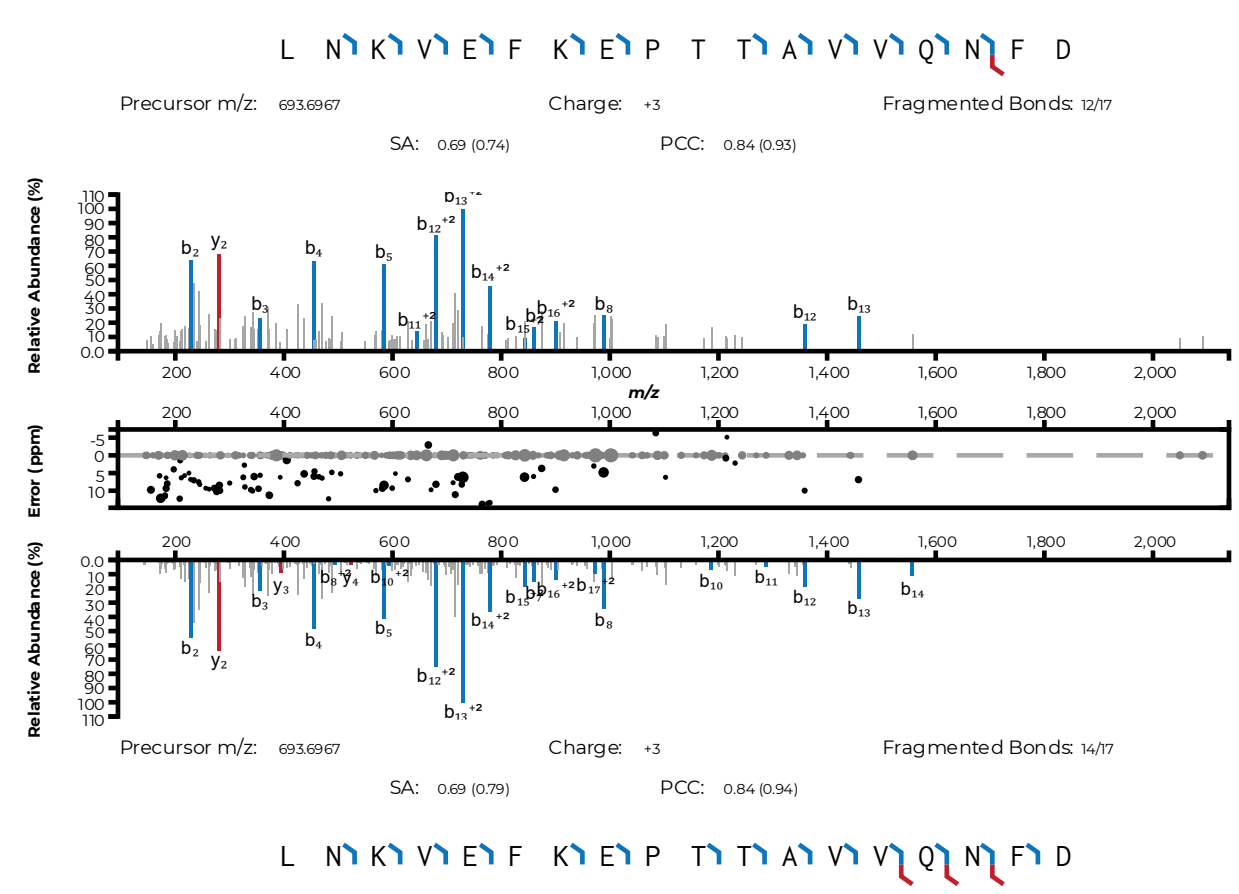

L N K V E F K E P T T A V V Q N F D
Precursor m/z: 693.6967
Charge: +3
Fragmented Bonds: 12/17
SA: 0.69 (0.74)
PCC: 0.84 (0.93)
Relative Abundance (%)
m/z
Error (ppm)
Relative Abundance (%)
Precursor m/z: 693.6967
Charge: +3
Fragmented Bonds: 14/17
SA: 0.69 (0.79)
PCC: 0.84 (0.94)
L N K V E F K E P T T A V V Q N F D

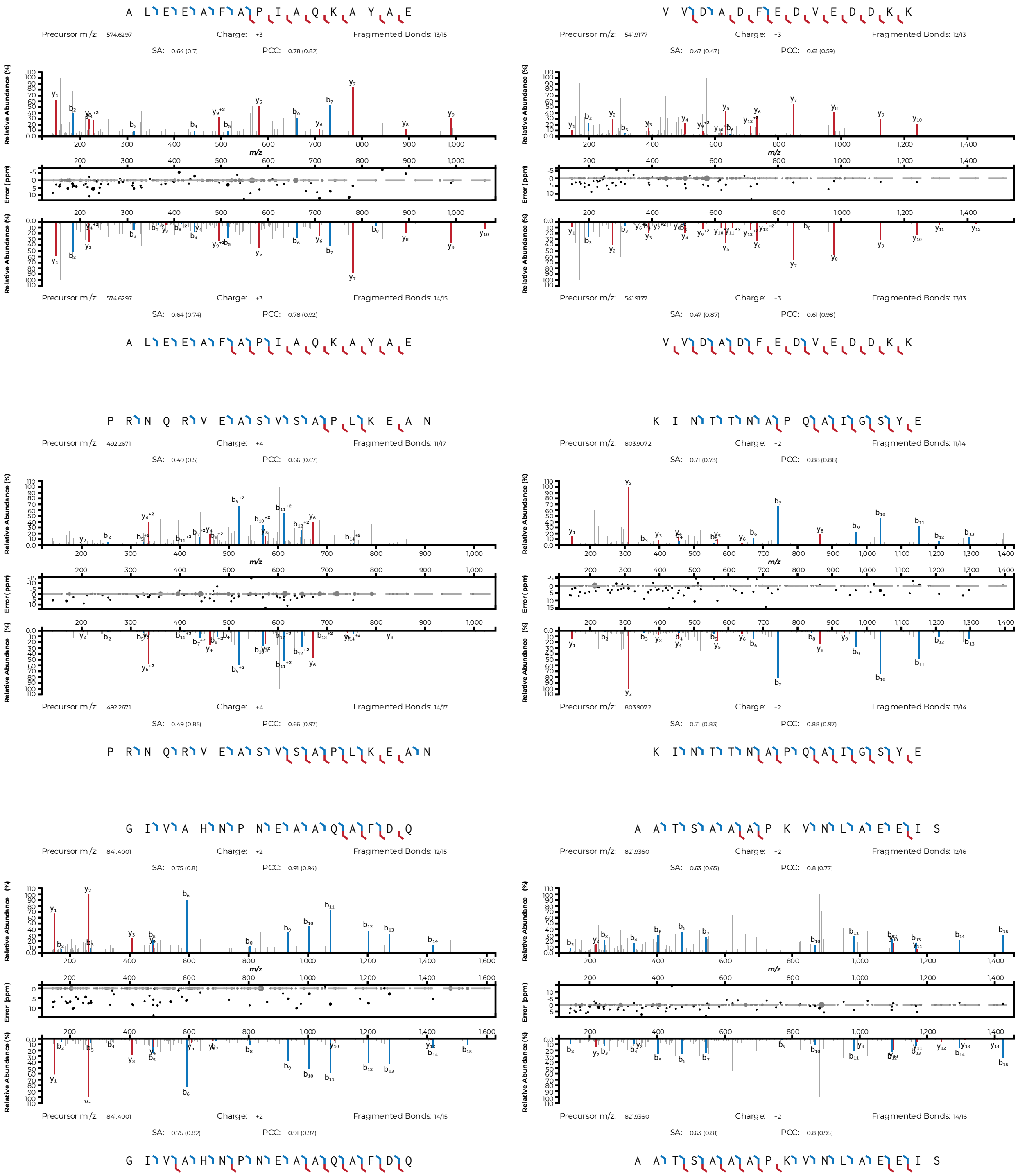

A L E E A F A P I A Q K A Y A E
Precursor m/z: 574.6297 Charge: +3 Fragmented Bonds: 13/15
SA: 0.64 (0.7) PCC: 0.78 (0.82)
Precursor m/z: 574.6297 Charge: +3 Fragmented Bonds: 14/15
SA: 0.64 (0.74) PCC: 0.78 (0.92)
A L E E A F A P I A Q K A Y A E
V V D A D F E D V E D D K K
Precursor m/z: 541.9177 Charge: +3 Fragmented Bonds: 12/13
SA: 0.47 (0.47) PCC: 0.61 (0.59)
Precursor m/z: 541.9177 Charge: +3 Fragmented Bonds: 13/13
SA: 0.47 (0.87) PCC: 0.61 (0.98)
V V D A D F E D V E D D K K
P R N Q R V E A S V S A P L K E A N
Precursor m/z: 492.2671 Charge: +4 Fragmented Bonds: 11/17
SA: 0.49 (0.5) PCC: 0.66 (0.67)
Precursor m/z: 492.2671 Charge: +4 Fragmented Bonds: 14/17
SA: 0.49 (0.85) PCC: 0.66 (0.97)
P R N Q R V E A S V S A P L K E A N
K I N T T N A P Q A I G S Y E
Precursor m/z: 803.9072 Charge: +2 Fragmented Bonds: 11/14
SA: 0.71 (0.73) PCC: 0.88 (0.88)
Precursor m/z: 803.9072 Charge: +2 Fragmented Bonds: 13/14
SA: 0.71 (0.83) PCC: 0.88 (0.97)
K I N T T N A P Q A I G S Y E
G I V A H N P N E A A Q A F D Q
Precursor m/z: 841.4001 Charge: +2 Fragmented Bonds: 12/15
SA: 0.75 (0.8) PCC: 0.91 (0.94)
Precursor m/z: 841.4001 Charge: +2 Fragmented Bonds: 14/15
SA: 0.75 (0.82) PCC: 0.91 (0.97)
G I V A H N P N E A A Q A F D Q
A A T S A A A P K V N L A E E I S
Precursor m/z: 821.9360 Charge: +2 Fragmented Bonds: 12/16
SA: 0.63 (0.65) PCC: 0.8 (0.77)
Precursor m/z: 821.9360 Charge: +2 Fragmented Bonds: 14/16
SA: 0.63 (0.81) PCC: 0.8 (0.95)
A A T S A A A P K V N L A E E I S
Relative Abundance (%)
Error (ppm)
m/z

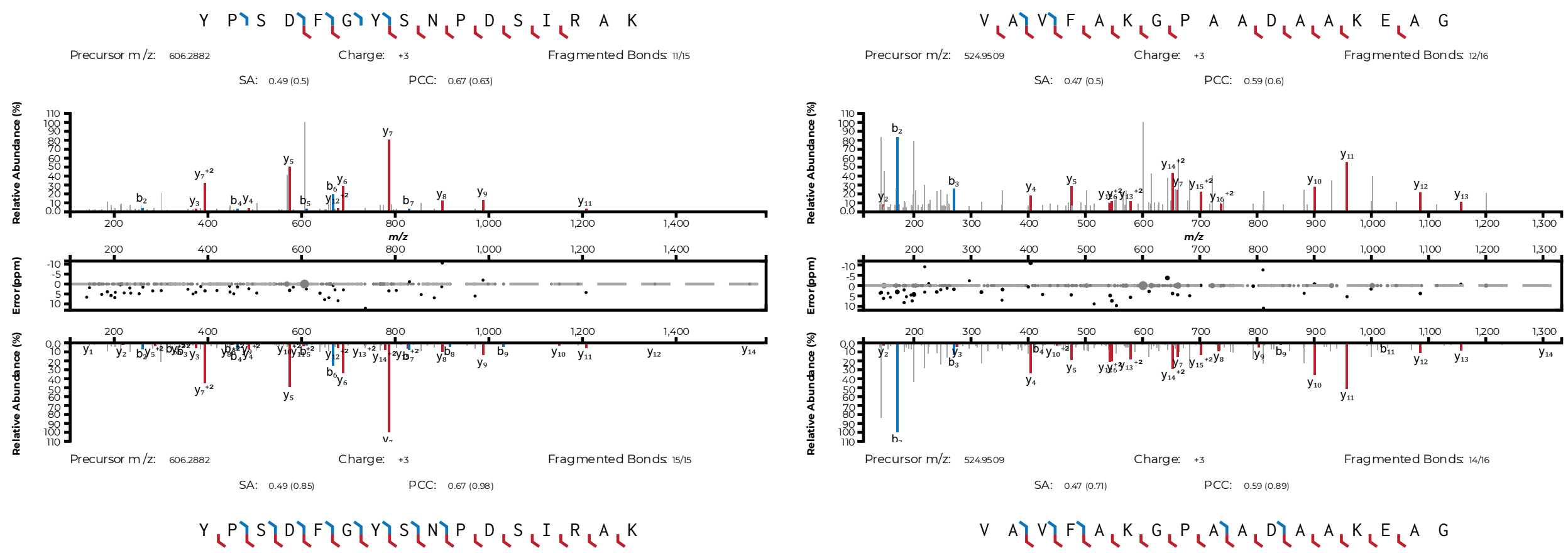

Fig B. Comparison of spectra of identified peptide epitopes with spectra of synthetic peptides. The spectra of 20 different peptide epitopes (top spectra) that were identified in the immunopeptidomes were compared for similarity with the spectra of the corresponding synthetic peptides (bottom spectra) using the Universal Spectrum Explorer tool (https://www.proteomicsdb.org/use/). Annotated b-ions, y-ions, and calculated spectrum similarity (SA, PCC) are shown. Pearson correlation was used as a measure for interpreting spectral similarity (PCC value in parentheses in the bottom spectrum indicating whether the spectrum of the synthetic peptide is contained in the spectrum of the peptide epitope).

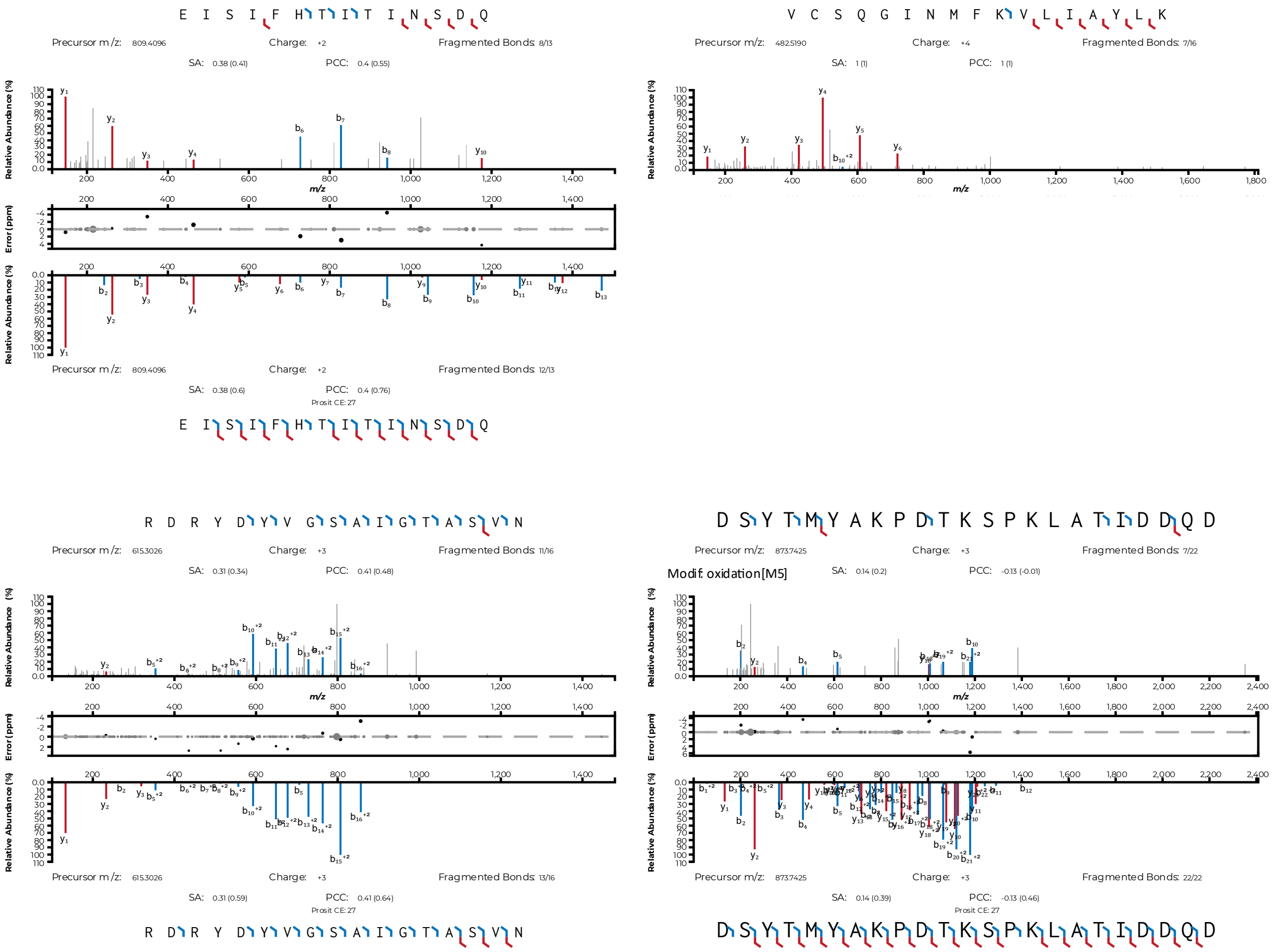

Fig C. Comparison of spectra of identified peptide epitopes with predicted spectra. The spectra of 4 different peptide epitopes (top spectra) that were identified in the immunopeptidomes but for which synthetic peptides were not available were compared for similarity to the predicted spectra of these peptides (bottom spectra) using the Universal Spectrum Explorer tool (https://www.proteomicsdb.org/use/). Annotated b-ions, y-ions, and calculated spectrum similarity (SA, PCC) are shown. Pearson correlation was used as a measure for interpreting spectral similarity (PCC value in parentheses in the bottom spectrum indicating whether the predicted spectrum is contained in the peptide epitope spectrum). No predictions were available for the peptide VCSQGINMFKVLIAYLK containing an unmodified cysteine.

Table A. Prediction of binding of homologous *F. tularensis* peptides to the I-A^b molecule.

Peptide	Core	Score	%Rank	Bind level	*Francisella* strain
IDGGEDVTKADSATAAASVIR	ADSATAAAS	0.021417	20.37		SCHU S4
VCSQGINMFKVLIVYLK	INMFKVLIV	0.000606	64.21		SCHU S4
ADEPEVNYTQPHSNINK	YTQPHSNIN	0.923468	0.1	SB	U112
EPEVNYTQPHSNINK	YTQPHSNIN	0.974966	0.03	SB	U112
TAATSAAAPAAPKVNVAEEIS	PAAPKVNVA	0.787265	0.37	SB	U112
TAATSAAAPAAPKVNVAEEISG	PAAPKVNVA	0.802458	0.32	SB	U112
AATSAAAPAAPKVNVAEEI	PAAPKVNVA	0.921078	0.11	SB	U112
AATSAAAPAAPKVNVAEEIS	PAAPKVNVA	0.85712	0.22	SB	U112
IEGGEDVTKADSATAAASVIR	ADSATAAAS	0.021416	20.37		LVS
VCSQGINMFKVLIAYLK	INMFKVLIA	0.00285	43.84		LVS
ADEPEINYTQPHSNINK	YTQPHSNIN	0.927666	0.1	SB	LVS
EPEINYTQPHSNINK	YTQPHSNIN	0.974137	0.03	SB	LVS
TAATSAAAPKVNLAEEIS	AAAPKVNLA	0.983594	0.02	SB	LVS
TAATSAAAPKVNLAEEISG	AAAPKVNLA	0.960056	0.05	SB	LVS
AATSAAAPKVNLAEEI	AAAPKVNLA	0.996507	0	SB	LVS
AATSAAAPKVNLAEEIS	AAAPKVNLA	0.992574	0.01	SB	LVS

Variations in the peptide sequence compared to the LVS strain are underlined.

Table B. MHC-II synthetic peptides of *Francisella* LVS tested for immunogenicity in *ex vivo* assays.

ORF	Gene name	Synthetic peptide	Short name	Pool-1	Pool-2	Pool-3	Pool-4
FTL_0113	iglC1	IEGGEDVTKADSATAAASVIR	iglC1-IR-21			PP-3	
FTL_0235	rpsJ	TGAQVKGPIPLPVRKE	rpsJ-TE-16		PP-2		
FTL_0262	rplQ	IELVDRPQVEEAADEE	rplQ-IE-16				
FTL_0519	minD	IIVTNPEVSSVRDSDR	minD-IR-16		PP-2		PP-4
FTL_0703	glyA	NVQPHSGSQANAAVYN	glyA-NN-16	PP-1			
FTL_0703	glyA	VQPHSGSQANAAVYN	glyA-VN-15				
FTL_0766	ggt	DGQYVYGAADPRRASAL	ggt-DL-17				
FTL_0879		ADEPEINYTQPHSNINK	ftl0879-AK-17		PP-2		
FTL_0987	mdh	LKTGSAYYAPAAAGIQM	mdh-LM-17				
FTL_1042		VTIAYKGTTPVIAYEDD	ftl1042-VD-17	PP-1			
FTL_1060	dacD	RSYIYNFPEAYKVYDDK	dacD-RK-17				PP-4
FTL_1137	fabF	IGMGGFAAARALSTRNDDPQ	fabF-IQ-20		PP-2		
FTL_1140	fabD	LNKVEFKEPTTAVVQNFD	fabD-LD-18	PP-1			
FTL_1191	dnaK	ALEEAFAPIAQKAYAE	dnaK-AE-16			PP-3	PP-4
FTL_1191	dnaK	VVDADFEDVEDDKK	dnaK-VK-14			PP-3	
FTL_1328	fopA1	PRNQRVEASVSAPLKEAN	fopA1-PN-18			PP-3	
FTL_1498		KINTTNAPQAIGSYE	ftl1498-KE-15	PP-1			PP-4
FTL_1553	sucC	GIVAHNPNEAAQAFDQ	sucC-GQ-16		PP-2		
FTL_1592	accB	AATSAAAPKVNLAEEIS	accB-AS-17	PP-1			
FTL_1678		YPSDFGYSNPDSIRAK	ftl1678-YK-16			PP-3	PP-4
FTL_1747	rplA	VAVFAKGPAADAAKEAG	rplA-VG-17				
FTL_0421	lpnA	RLQWQAPEGSKCHD†	Positive (lpnA-RD-14)				
	Gls	YAIAVNDLGTEYVHR‡	Negative (Gls-YR-15)				

Each peptide is labeled with the open-reading frame (ORF) and gene name of its source protein, and a short name composed of the gene name, the first and last amino acids of the peptide, and the sequence length. The composition of the peptide pools (PP) used in the study is shown. †The peptide was not identified in our study, but was used as a positive control. ‡Peptide of mouse Glutaminase (Gls) was used as a negative control.

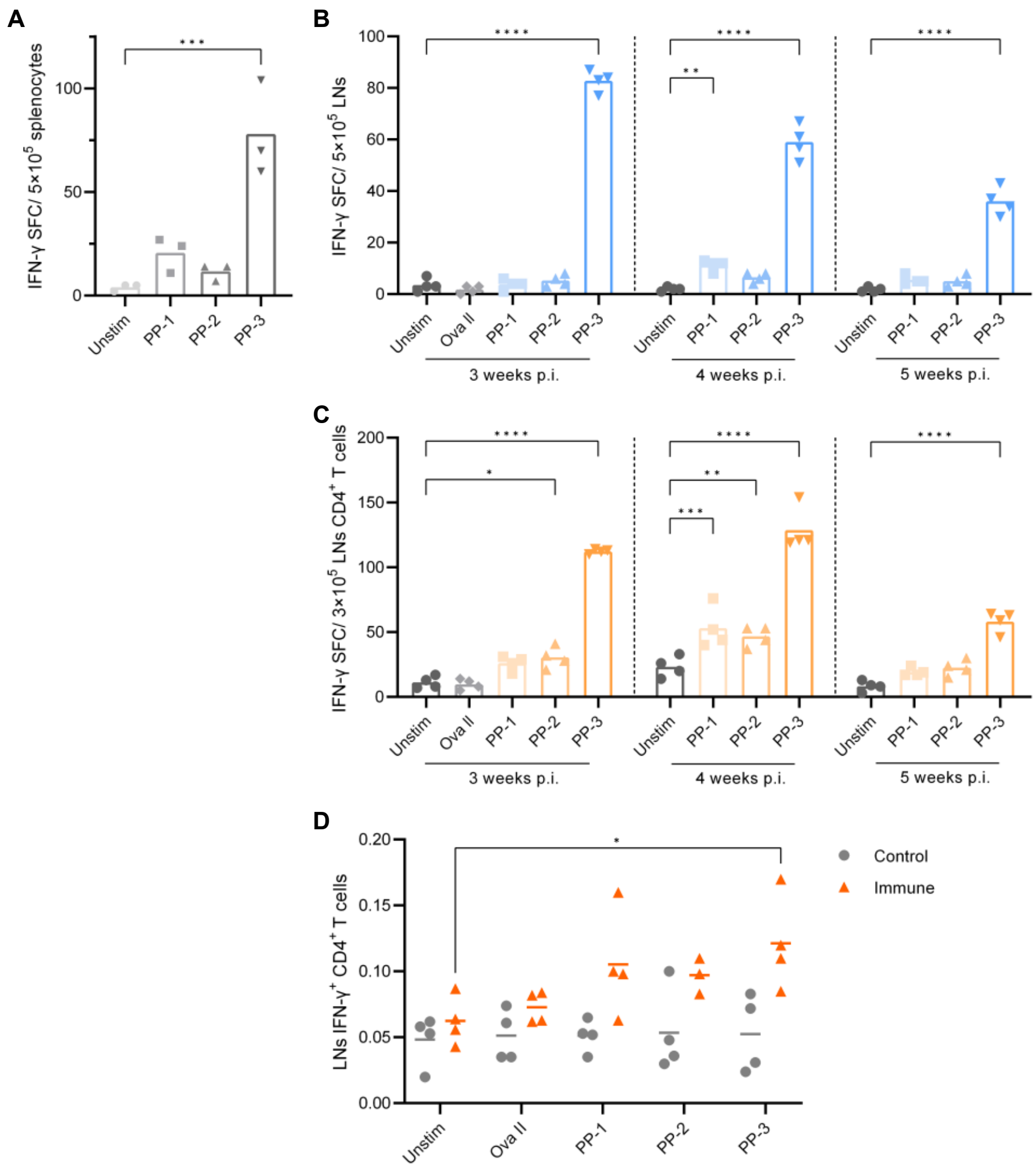

Fig D. Pools of *Francisella* MHC-II peptides elicit time-dependent and CD4⁺ T cell-mediated IFN-γ response in immune lymphocytes isolated from lymph nodes. C57Bl/6 mice were subcutaneously injected with 3×10^4 CFU of *F. tularensis* LVS and immune lymphocytes, pooled from groups of 3-4 mice, were stimulated with bacterial MHC-II peptides assembled in pools for immunogenicity assessment. (A) Initial screening of the potential of peptide pools (PPs) to stimulate IFN-γ producing cells (spot-forming colonies (SFCs)) was performed with 5×10^5 LVS-immune whole splenocytes isolated 4 weeks p.i. Data of individual assayed wells per condition and mean values are shown. PPs with significant response against a control without peptides (unstim) were detected with one-way ANOVA followed by Dunnet's post hoc test. (B, C) Time-dependent profiles of responses elicited by PPs assessed in whole-cells suspensions or $CD4^+$ T cells from LNs of immunized mice (the same mice, as for analysis of spleen lymphocytes shown in Fig 3). The numbers of IFN-γ producing cells (SFCs) were determined at 3, 4 and 5 weeks p.i. following stimulation of (B) 5×10^5 whole-cells LNs suspensions or (C) cocultures of 3×10^5 LNs $CD4^+$ T cells and 3×10^4 BMDCs with peptide pools (PP). Data of individual assayed wells per condition and mean values are shown. PPs with significant response against control without peptides (unstim) were detected with one-way ANOVA followed by Šídák's post hoc test. (D) Cocultures of 3×10^5 LNs $CD4^+$ T cells and 3×10^4

BMDCs with PPs for 24 h. Intracellular IFN-γ production was determined by FACS analysis after incubation of cells with Brefeldin A for the last 4 h. T cells were isolated from mice 4 weeks after application of *F. tularensis* LVS (immune) or sterile PBS (control). Each dot represents one technical replicate and mean values are shown. PPs with significant response against control without peptides (unstim) were detected with two-way ANOVA followed by Tukey's post hoc test. PPs representing a subset of 15 peptides (Table 1 in S1 File) were used for all assays with a 5 µM final concentration of each peptide. Ova II peptide (5 µM) was used as a negative control in some experiments. Asterisks indicate p-values (*p< 0.05, **p< 0.01, ***p< 0.001, ****p< 0.0001). Data are representative results of two biological replicates (A, D) or one experiment (B, C).

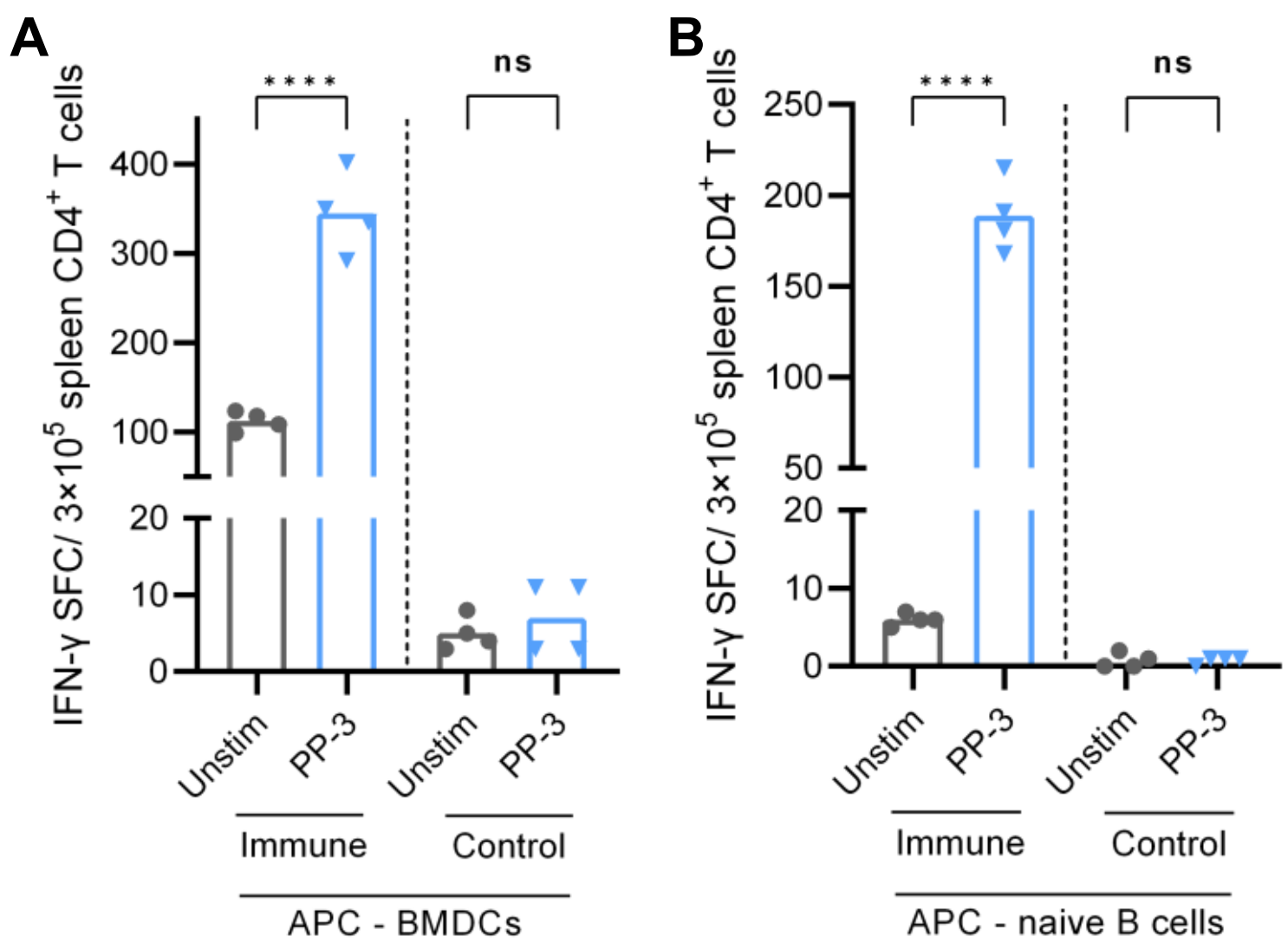

Fig E. The use of B cells instead of BMDCs as antigen presenting cells in cocultures with CD4⁺ T cells results in increased sensitivity of the ELISpot assay. C57Bl/6 mice were subcutaneously injected with either 3×10^4 CFU of *F. tularensis* LVS (immune) or sterile PBS (control) and lymphocytes were isolated 4 weeks later. B cells were purified from spleen of age-matched naïve mice. The effect of various antigen presenting cells on the levels of IFN-γ producing cells (SFCs) was determined following stimulation of (A) 3×10^5 $CD4^+$ T cells and 2×10^4 BMDCs or (B) 3×10^5 $CD4^+$ T cells and 4×10^5 naïve B cells with the most reactive PP-3. Data of individual assayed wells per condition and mean values are shown. PP-3 induced significant responses (two-tailed t-test) only in immune lymphocytes with a signal-to-noise ratio of 3.1 and 31.5, and a net reactivity of 232 and 183 for BMDCs or B cells antigen presenting cells, respectively. Concentration of each peptide in the PP-3 (Table 1 in S1 File) was 5 µM. Asterisks indicate p-values (****$p < 0.0001$, ns for $p \geq 0.05$). The data is the result of one experiment.

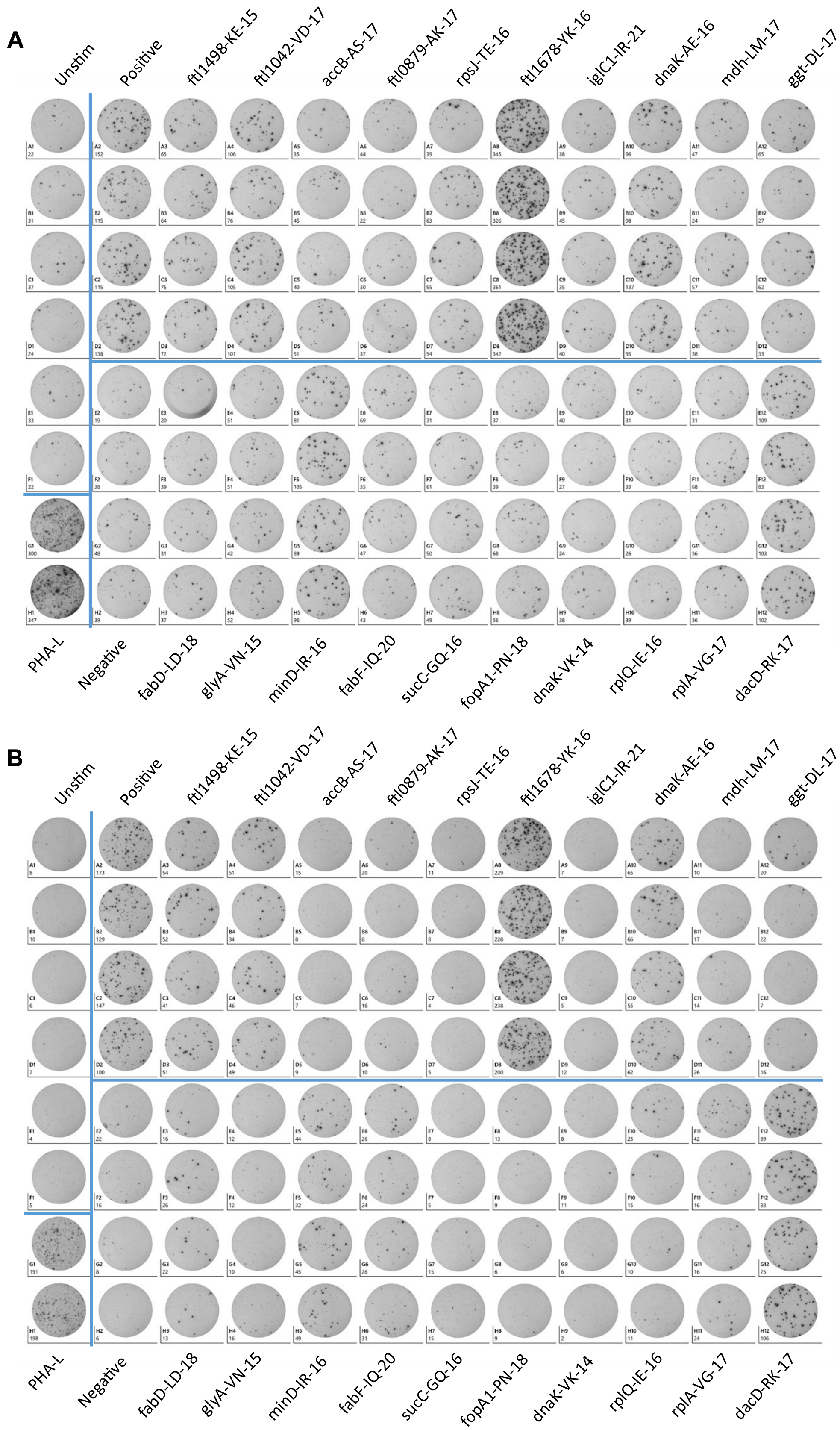
A
Unstim
Positive
ftl1498-KE-15
ftl1042-VD-17
accB-AS-17
ftl0879-AK-17
rpsJ-TE-16
ftl1678-YK-16
iglC1-IR-21
dnaK-AE-16
mdh-LM-17
ggt-DL-17
PHA-L
Negative
fabD-LD-18
glyA-VN-15
minD-IR-16
fabF-IQ-20
sucC-GQ-16
fopA1-PN-18
dnaK-VK-14
rplQ-IE-16
rplA-VG-17
dacD-RK-17
B
Unstim
Positive
ftl1498-KE-15
ftl1042-VD-17
accB-AS-17
ftl0879-AK-17
rpsJ-TE-16
ftl1678-YK-16
iglC1-IR-21
dnaK-AE-16
mdh-LM-17
ggt-DL-17
PHA-L
Negative
fabD-LD-18
glyA-VN-15
minD-IR-16
fabF-IQ-20
sucC-GQ-16
fopA1-PN-18
dnaK-VK-14
rplQ-IE-16
rplA-VG-17
dacD-RK-17

Fig F. Representative ELISpot scans showing IFN-γ-producing cells following stimulation of lymphocytes with *Francisella* peptides. C57Bl/6 mice were subcutaneously injected with 3×10^4 CFU of *F. tularensis* LVS and immune lymphocytes were stimulated with *Francisella* MHC-II peptides to assess immunogenicity. Lymphocytes were pooled from groups of 3-4 mice 3 weeks p.i. Images show the results (ASTOR 2, Mabtech) obtained following stimulation of (A) 5×10^5 whole splenocytes or (B) cocultures of 3×10^5 spleen $CD4^+$ T cells and 2×10^5 naïve B cells with individual peptides. Wells containing lymphocytes and medium only (Unstim, 6 replicates), 4 µg/ml PHA-L (2 replicates) or 10 µM individual peptides (4 replicates) are labeled. Peptide RLQWQAPEGSKCHD (*Francisella* LpnA) and YAIAVNDLGTEYVHR (mouse Gls) were used as positive and negative peptide, respectively. The number of spot-forming colonies is shown at the bottom of each well. Data are representative results of 4 biological replicates included in Fig 4A, B in the main text.

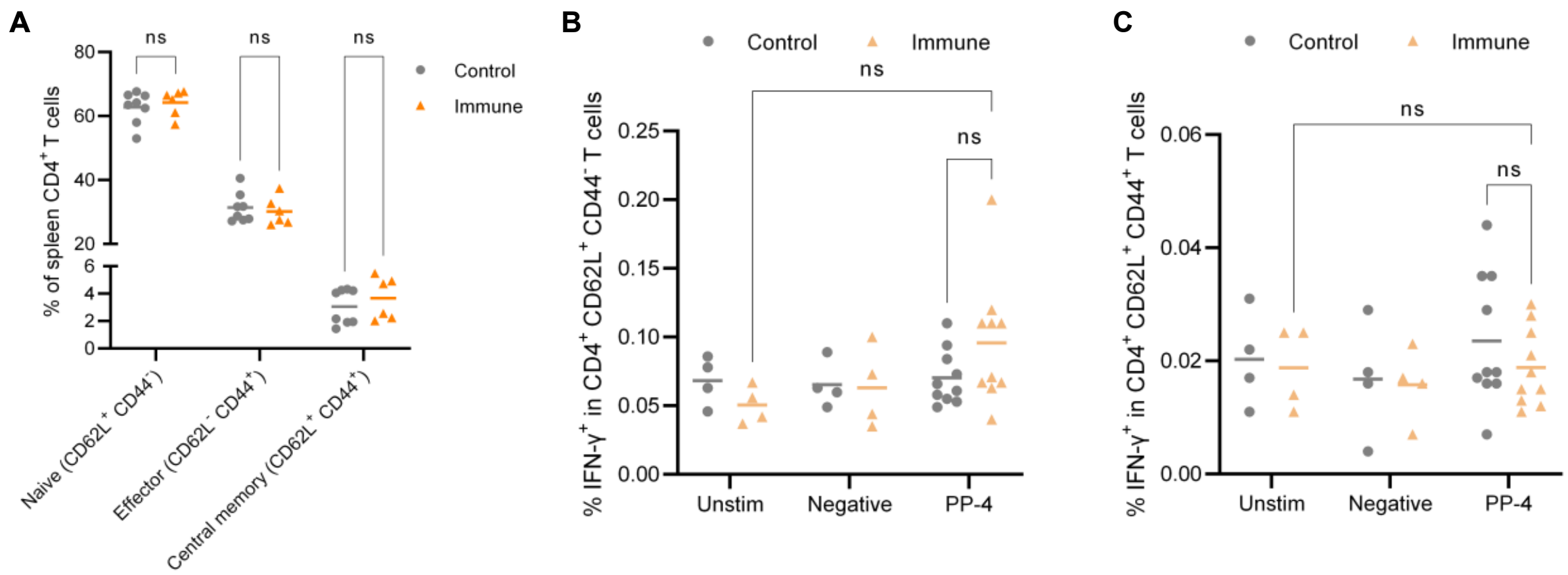

Fig G. Phenotype of CD4⁺ T cells. C57Bl/6 mice were subcutaneously injected with either 3 × 10^4 CFU of *F. tularensis* LVS (immune) or sterile PBS (control), and lymphocytes were isolated 3 weeks later and subjected to FACS analysis to evaluate $CD4^+$ T cell subpopulations. (A) Results from analysis of all live splenic $CD4^+$ T cells. Each dot represents one analyzed mouse spleen and mean values are shown. Differences between control and immune groups were determined with two-way ANOVA followed by Šídák's post hoc test. (B, C) Cocultures of 3 × 10^5 splenic $CD4^+$ T cells and 2 × 10^5 splenic B cells with PP-4 or without (unstim) for 24 h. Cells were incubated with Brefeldin A for the last 4 h and intracellular IFN-γ production was determined in defined T cell subpopulations. Each dot represents one technical replicate and mean values are shown. Differences between groups were determined with two-way ANOVA followed by Tukey's post hoc test. The analyses are related to Fig 5 in the main text. PP-4 contained 5 selected immunogenic peptides (Table 1 in S1 File) and a 5 μM final concentration of each peptide was used. Peptide YAIAVNDLGTEYVHR (mouse Gls) was used as a negative peptide (negative). NS indicates p-values ≥ 0.05. Data are combined results of two biological replicates.

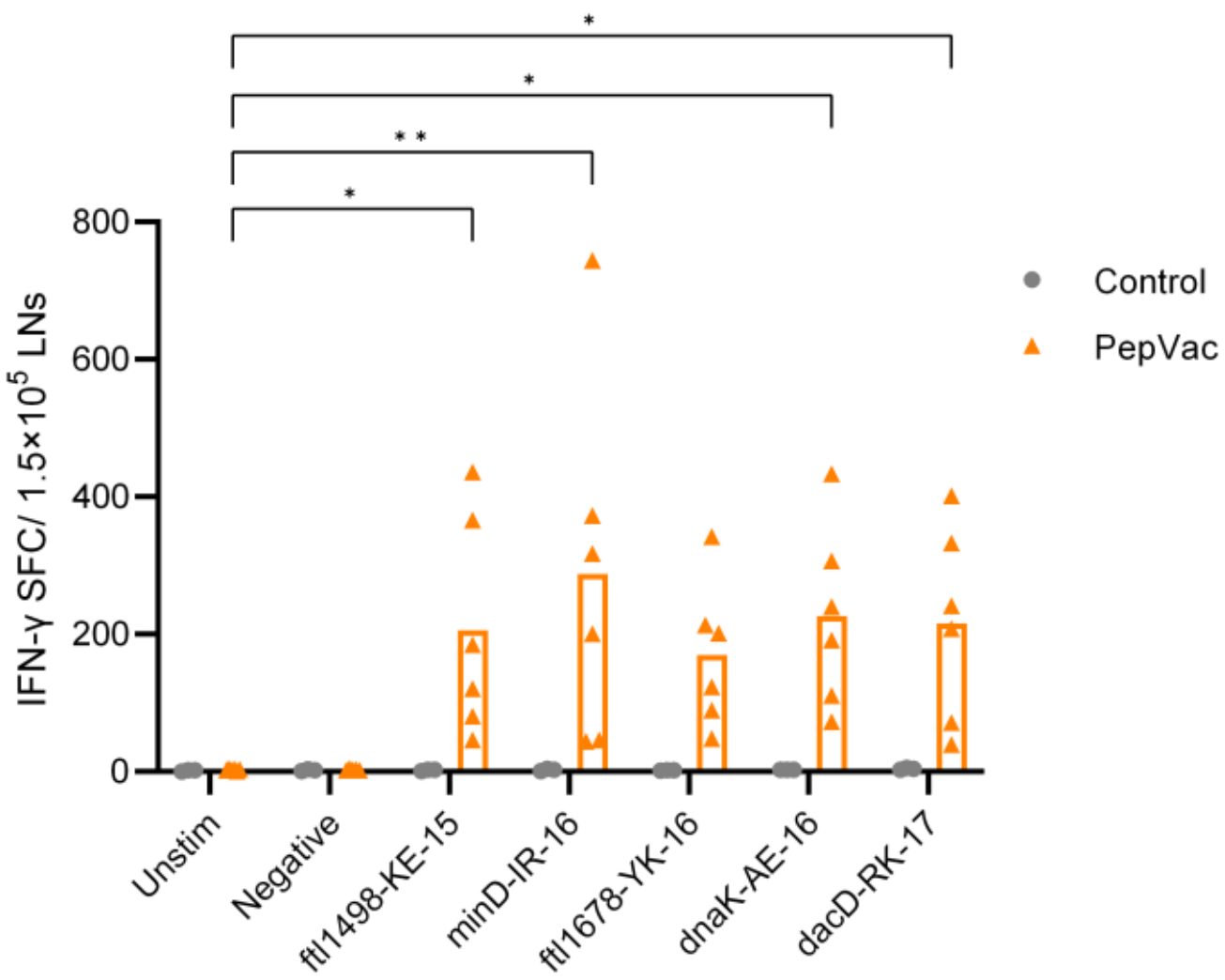

Fig H. Cellular response induced by a peptide vaccine in lymph nodes. C57Bl/6 mice were immunized with a peptide vaccine containing five peptide epitopes of *Francisella* (ftl1498-KE-15, minD-IR-16, ftl1678-YK-16, dnaK-AE-16, dacD-RK-17), and the number of IFN-γ-producing cells in lymphocytes from the subiliac lymph nodes was determined using the ELISpot assay. Responses were determined in individual mice from the peptide vaccine group (PepVac, n= 6) or the control group (control, n= 3), which received the same adjuvant formulations but without peptides. Measurements were performed on days 13, 17 and 19 after the booster dose (due to capacity reasons) and the data are combined in this graph. Values for individual mice (points) and their averages are shown. Peptides were used at a final concentration of 5 µM, and control wells without peptide (unstim) or with a negative peptide (YAIAVNDLGTEYVHR) were included. Significant differences compared to the control (unstim) were identified using two-way ANOVA followed by Dunnett's post hoc test. Asterisks indicate p-values ($*p < 0.05$, $**p < 0.01$). This experiment was performed once.

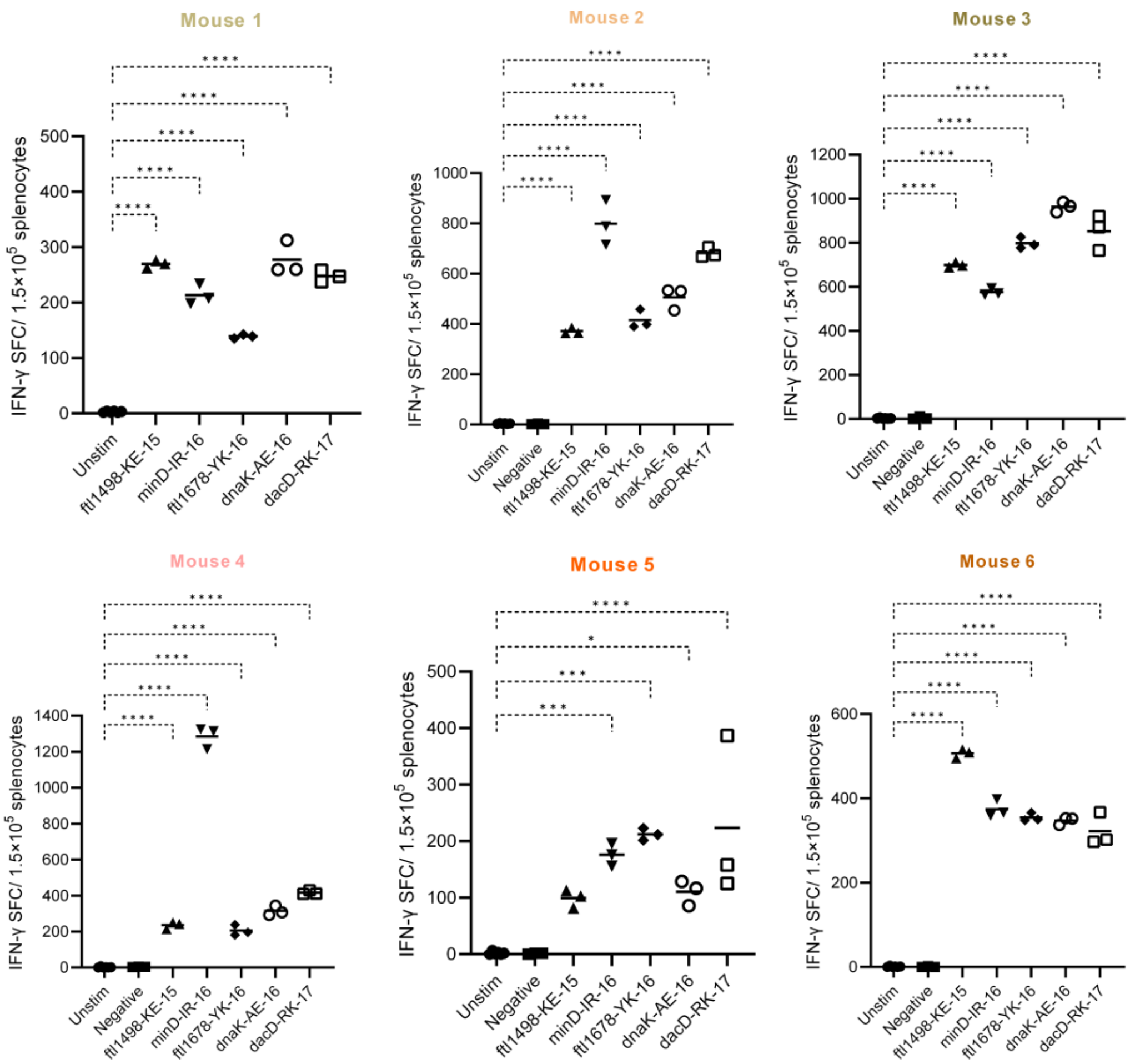

Fig I. Variability of responses in vaccinated mice. Mice were immunized with a peptide vaccine containing five peptide epitopes of *Francisella*, and the number of IFN-γ-producing cells in splenocytes was determined following stimulation with the peptides contained in the vaccine. Responses in individual mice (1-6) are shown using data representing individual wells for each condition and corresponding mean values. Peptides were used at a final concentration of 5 µM, and control wells without peptide (unstim) or with a negative peptide (YAIAVNDLGTEYVHR) were included. Significant differences compared to the control (unstim) were identified using one-way ANOVA followed by Dunnett's post hoc test. Asterisks indicate p-values (*p< 0.05, ***p< 0.001, ****p< 0.0001). This experiment was performed once.
