# Supplementary material for: Immunopeptidomics reveals conserved and abundant Francisella tularensis proteins as immunogenic CD4 T cell antigens in mice
Source: PLoS Pathog. 2026 Sep 29;22(9):e1014597. doi: 10.1371/journal.ppat.1014597 (PMC13649117; doi:10.1371/journal.ppat.1014597)
Supplement: Supplementary file 2 [file ppat.1014597.s007.pdf]

Dear Dr. Carlyon,

Thank you for the opportunity to revise and resubmit our manuscript, titled “Immunopeptidomics reveals conserved and abundant Francisella tularensis proteins as immunogenic CD4 T cell antigens in mice” (PPATHOGENS-D-25-02289), for consideration in PLOS Pathogens.

We appreciate the thoughtful and constructive comments provided by the reviewers. We have carefully addressed all the feedback and made significant revisions to the manuscript accordingly.

We have included a detailed response to the reviewers' comments.

We believe these revisions have strengthened the manuscript, and we hope it will now meet the journal’s standards for publication. We sincerely thank you and the reviewers for your time and consideration.

Please do not hesitate to contact us if any further information is needed.

Yours sincerely,

Marek Link

Department of Molecular Pathology and Biology

Military Faculty of Medicine, University of Defence

Trebesska 1575, 500 01 Hradec Kralove

Czech Republic

e-mail: marek.link@unob.cz

Reviewer #1

Summary

Overall, the paper is a valuable discovery and validation work for Francisella tularensis-specific murine epitopes of two vaccine strains, LVS strain versus the LVSΔdsbA. This work will clearly inform work performed in this mouse model, however, the connection to the human system, and as quoted as motivation, remains a little unclear.

We thank the Reviewer for the time and effort devoted to evaluating our manuscript. We appreciate the positive comments regarding our experiments and discovery. We have considered all comments and concerns raised, and revised the manuscript accordingly to improve its clarity, rigor, and overall presentation.

Major Issues:

As a reader unfamiliar with this pathogen, I found it necessary to look up the host range and human virulence of this pathogen online. The manuscript would benefit from a clearer description of the biological context, motivation, and overall scope of the study. Please expand on how the mouse model relates to human clinical data and specify the species you are working with throughout the manuscript, please. Also, the LVSΔdsbA mutant is not introduced, and it is hard to understand why the authors seek the comparison between wt and mutant strain in the first instance.

We have improved the introduction of the manuscript and supplemented the missing information to the appropriate extent with references to specialized reviews for more detailed insight. New reference on the ecology of *Franciella tularensis* was included. We thank the reviewer for pointing out the introduction of the LVSΔdsbA mutant. The characterization of the LVSΔdsbA phenotype and the idea of its use in BMDCs infection and immunopeptidome analysis are now included in the introduction.

In Figure 1, The statement "The presence of specific peptide only (or predominantly) in the infected samples helped to identify the bacterial peptides" is currently unclear and would benefit from further clarification. Please indicate the exact number of bacterial peptide sequences identified in both infected and uninfected samples. It would be helpful to explain why identifications are obtained from uninfected cells; this observation needs to be clearly communicated to the reader and carefully explained.

We apologies for the confusing statement in Figure 1 and uncomplete description of peptide identification results. We changed description in Fig 1C to "(C) Extracted ion chromatograms for the peptide identified in (B) from analyses of uninfected, LVS-infected, and LVSΔdsbA-infected BMDCs." In the "Bacterial MHC-II peptides represent …" section of Results, we have paid attention to carefully describe the peptide identification results and manual curation of the identified bacterial peptides.

We reported the numbers of identified bacterial peptides and excluded bacterial peptides along with the justification.

You state that most peptides were identified using the match-between-runs feature. This approach is generally discouraged in immunopeptidomics discovery studies. Please provide additional evidence supporting that these peptides are genuinely present in the samples or clarify how this analytical choice affects confidence in the identifications.

We apologies for the confusing statement. We used peptide features matching and peptide extracted ion chromatograms for two purposes. First, to assess the specificity of bacterial peptide identification (comparison of infected and control samples). Second, as “evidence” that a peptide sequenced and identified by PSM in one sample is also present in another sample in which the peptide was not directly sequenced (comparison of LVS and LVSΔdsbA samples). We use this “evidence” in the same way as in standard label-free comparative studies. We are aware of the potential limitations of peptide features mapping and have manually verified the signal traces of all bacterial peptides.

We supported the identification of bacterial peptide sequences by spectral similarity analysis, which showed high correlation coefficients between experimental and reference spectra for most peptides. Synthetic peptide spectra (20 peptides) or predicted spectra (3 peptides) were used as references for similarity analysis. Results of this analysis were included in the “Bacterial MHC-II peptides represent …” section of Results, and the Fig 2, 3 in S1 File. Description of the analysis was included in “Bioinformatics” section of Materials and Methods with appropriate reference.

The authors should include a brief discussion of the observed differences in length distribution between self-derived and bacterial peptides - I did not see this mentioned in the text, or discussed.

We thank the Reviewer for bringing this to our attention. We included a brief discussion (Discussion, second paragraph) of this observation, that is based on different activation of infected and uninfected cells. We have found one publication (ref 52, Marino F. et al., 2020) showing shift of MHC-II peptides toward longer sequences following stimulation of dendritic cells or B cells. In our experiments, the endogenous MHC-II peptides likely originate from differently activated BMDCs (uninfected, infected, bystander cells) which could also impact the resulting distribution of peptide lengths.

Conservation results section. Page 11, line 201. The statement “…were unique to the bacterium…” could be clarified. Does this refer to peptides unique to the bacterial strain? Presumably, peptides with sequences identical to those in the mouse proteome would be excluded from bacterial assignments. Please revise this section to make this point explicit. A short motivation in why you are carrying out this analysis would be helpful to the reader.

We thank the Reviewer for this comment. We have edited the section accordingly, with an emphasis on its clarity.

My understanding is that the term "epitope region" refers to overlapping MHC-II–bound peptide sequences, which are characteristic of class II presentation. Please confirm if this interpretation is correct and consider clarifying this definition in the text more clearly. Including a schematic or example figure would help prevent potential confusion.

We thank the Reviewer for this comment and constructive suggestion. The term "epitope region" really referred to the part of the protein from which overlapping MHC-II peptides were derived. We have clarified this definition in the text and added a schematic image for visualization (Fig 1I, which replaces the original bar graph).

In the section "Francisella MHC-II antigens originate from a highly abundant fraction of the bacterial proteome" and in the corresponding conclusion, the claim that "Francisella antigens are sampled into the MHC class II pathway from a highly abundant fraction of its proteome" may be overstated. The data likely reflect the sensitivity limits of the immunopeptidomics experiment, which may favor detection of peptides from more abundant proteins. Please discuss this limitation and temper the conclusion accordingly.

We thank the Reviewer for pointing this out and agree that our conclusion was overstated. We have tempered the conclusion in the "Francisella MHC-II antigens originate from …" section of Results and elsewhere in the manuscript. We briefly mention the bias of immunopeptidomics to detect more abundant peptides in discussion.

Minor Issues:

On page 8, line 155, the relative proportion of predicted bacterial peptide binders compared to self-peptides is not clearly described or visualized. Please quantify this comparison and, if possible, illustrate it explicitly in a figure.

We thank the Reviewer for this comment. We have improved the description of the peptide binding results in the "Bacterial MHC-II peptides represent …" section of Results and data visualization (Fig 1F, which replaces the original x-y plot).

Reviewer #2

Summary

In their paper they discuss that vaccination against Francisella using the LVS strain involves the action of CD+ T cell cells, but antigens targeted from these infections are now know. To uncover potential antigens they purified MHC-II and identified displayed bacterial peptides by mass spec. From this they identified peptides from 23 different proteins. They went on to validate many of these peptides as MHC-II displayed, and showed they were capable if stimulating splenocytes and CD4+ T cells. Their results identified new highly immunogenic peptides not previously identified and found some even more immunogenic than previously known targets. Overall, their data seems rigorous with appropriate replicates and well presented. The main thing that hold the paper back is significance due to the lack of functional immunity experiments. Experiments vaccinating with their synthesized peptides and showing protections against Francisella infection or maybe reduced infection burden vs the LVS strain could really boost significance. Short of that, perhaps showing sera from that peptide immunized animals can recognize LVS proteins. The paper would be an accept from me at a more vaccine specific journals or Plos One where similar studies they referenced were published, but without functional protection data their findings are less impactful for a larger Plos Pathogenesis audience.

We thank the Reviewer for the time and effort devoted to evaluating our manuscript. We appreciate the positive comments regarding the quality of our experiments and the interpretation of our findings. We have addressed the suggestion for functional immunity experiments in the revised manuscript.

Major Issues:

Impact of study limited by lack of functional immunity experiments to wrap it up. While their results find the peptides are immunogenic, they don't show protection. Experiments vaccinating mice with the peptides could boost the studies impact.

We thank the Reviewer for this suggestion. To be honest, these experiments were a challenge for us, but they helped us improve our skills and broaden our horizons. To address this issue, we immunized mice with a peptide vaccine containing five major peptide epitopes of *F. tularensis* LVS. We demonstrated that all peptides were immunogenic and elicited a strong Th1-type cellular immune response. In the next step, we tested the vaccines' protective potential against infection with the *F. tularensis* subsp. *holarctica* FSC200 strain, which is highly virulent in mice. We demonstrated that the induced immune response contributed to early control of the bacterial load in organs and reduced disease severity. However, the vaccine's potential was insufficient to provide full protection against infection with this virulent strain. Nevertheless, we believe that the demonstrated immunogenic potential of these epitopes and their recognition during infection may be valuable for future protective studies.

The description of these findings was included into the Results ("A vaccine containing *Francisella* peptide epitopes is immunogenic in mice", and "Protective efficacy of peptide vaccine against *F.*

tularensis FSC200 infection", and Fig 6, and Fig 7), and S1 file (Fig 8, Fig 9), together with the respective sections of Materials and Methods ("Immunization of mice", and "Protective study"). The findings are also part of discussion.

Minor Issues:

(No Response)

Other changes

Ales Neuwirth was added as a co-author – he contributed to the experimental work during the revision process
