# Supplementary material for: Immunopeptidomics reveals conserved and abundant Francisella tularensis proteins as immunogenic CD4 T cell antigens in mice
Source: PLoS Pathog. 2026 Sep 29;22(9):e1014597. doi: 10.1371/journal.ppat.1014597 (PMC13649117; doi:10.1371/journal.ppat.1014597)
Supplement: Supplementary file 3 [file ppat.1014597.s008.pdf]

Dear Dr. Carlyon,

Thank you for the opportunity to revise and resubmit our manuscript, titled “Immunopeptidomics reveals conserved and abundant Francisella tularensis proteins as immunogenic CD4 T cell antigens in mice” (PPATHOGENS-D-25-02289R1), for consideration in PLOS Pathogens.

We appreciate the insightful comments from the reviewers. We have carefully addressed all the feedback and revised the manuscript accordingly.

We have included a detailed response to the reviewers' comments.

We believe that these revisions have improved the manuscript, and we hope that it now meets the journal’s standards for publication. We sincerely thank you and the reviewers for your time and consideration.

Please do not hesitate to contact us if any further information is needed.

Yours sincerely,

Marek Link

Department of Molecular Pathology and Biology

Military Faculty of Medicine, University of Defence

Trebesska 1575, 500 01 Hradec Kralove

Czech Republic

e-mail: marek.link@unob.cz

Reviewer #2

Part I – Summary

The new vaccination data didn't provide strong protection, but the reduced burden in the organs is encouraging. Overall presents valuable information for the field. I have some minor concerns/questions about number or animals and significance, but feel the paper is improved with the new findings and edits.

We would like to thank the Reviewer for the time and effort devoted to evaluating our manuscript. We appreciate the positive feedback regarding the contribution of our experiments. We have addressed the questions raised with the aim of improving the presentation and interpretation of the data.

Part II – Major Issues

In line 488-489 They state that the vaccinated group weight loss was delayed. Given the small number of animals and high variability I'm concerned about the significance there. I suggest either rewording or determining significance.

We thank the Reviewer for this comment and suggestion. We performed a new analysis of the mouse weight loss data using a mixed-effects model with repeated measures followed by multiple comparisons between groups at individual time points. Only data from 1 to 5 days postinfection were included in this analysis, as no mice succumbed to the infection during these time intervals. This analysis revealed a statistically significant difference in the course of weight loss between vaccinated and control mice, although the differences at individual time points were not significant. The results of this analysis were included in the "Results" section of the manuscript (the paragraph "Protective efficacy of peptide vaccine against F. tularensis FSC200 infection", the caption for Fig 7, and Fig 7B).

In figures 6 and 7 the number of mice between control and treatment groups are different. Why the mismatch

In Figure 6, the uneven group sizes were due to the limited availability of animals during these experiments. We prioritized assigning animals to the vaccinated group in order to best evaluate the immunogenicity of the peptides, while maintaining an adequate, albeit minimal, control group for assessing its reactivity and for statistical comparisons.

In Figure 7, the uneven group sizes were due to the fact that one mouse was lost after receiving a booster dose as part of the immunization. The mouse died after the intraperitoneal injection.

We mentioned these reasons in the captions for respective figures.

Part III – Minor Issues

(No Response)

Reviewer #3

Part I – Summary

The submission is a revised manuscript that identifies epitopes from F. tularensis and shown to be immunogenic in mouse models. However, when vaccinating mice with the best performing peptides and then challenging subcutaneously with the FSC200 strain, no differences in protection were observed as compared to naive mice. However, when looking at CFU recovery rates from organs of the challenged mice, a statistical difference in reduced recovery was observed with the vaccinated mice. As not an original reviewer, the authors appeared to have responded to the previous reviewers request. However, this reviewer would suggest some additional revisions though not laboratory work based.

We would like to thank the Reviewer for the time and effort devoted to evaluating our manuscript. We have considered all comments raised and revised the manuscript accordingly.

Part II – Major Issues

Much effort has also been placed in the T cell responses to the Schu S4 clpB mutant. It would be helpful to incorporate the results found with this vaccine strain and how it relates to the work performed here.

We thank the Reviewer for this comment and suggestion. Studies using the live attenuated SCHU S4-ΔclpB vaccine have shown that its higher protective efficacy compared to the LVS vaccine can be attributed to the induction of more effective T cell-mediated immune responses. The results of these studies were incorporated into the "Introduction" section (lines 96-99) and the "Discussion" section (lines 529-531) of the manuscript.

Protection studies with immunized mice and FSC200 challenge:

A) What is the rationale of performing challenges by subQ versus intranasal (pneumonic model). Obviously, this relates to the public health threat versus biodefense. This should be included. Do the authors believe the results would have been any different with an intranasal challenge?

We thank the Reviewer for this comment. We have added an explanation for the choice of the subcutaneous route of infection for the challenge experiments to the "Results" section of the manuscript (paragraph "Protective efficacy of peptide vaccine against F. tularensis FSC200 infection", lines 487-490).

Although the peptide vaccine induced a systemic antigen-specific cellular immune response, we cannot predict the degree of protection that would be observed following intranasal challenge. Previous studies of pneumonic tularemia have shown that protection depends on contributions of lung-resident and circulating T cell populations (Roberts et al. 2018, Roberts et al. 2022). These T cell compartments were not specifically evaluated in the present study; therefore, the effect of our vaccination strategy against intranasal challenge remains to be determined experimentally. Ref.:

Roberts LM, et al. Temporal Requirement for Pulmonary Resident and Circulating T Cells during Virulent *Francisella tularensis* Infection. J Immunol. 2018. doi: 10.4049/jimmunol.1800052. Roberts LM, et al. Circulating T Cells Are Not Sufficient for Protective Immunity against Virulent *Francisella tularensis*. J Immunol. 2022. doi: 10.4049/jimmunol.2100915).

B) The one area that should be addressed in this manuscript is the issue of using mice for tularemia vaccine studies. It is well established that mice are not ideal for vaccines versus establishing virulence differences. Obviously despite these limitations, mice are still a starting point for vaccine studies due to numerous reasons (mostly financial). Perhaps the authors should consider performing these protection studies in rats, a better model for tularemia vaccines versus mice. This could be a next step, so the authors should expand on this point (lines 622-626).

We thank the Reviewer for this comment and suggestion. We have included the issue of using mice versus rats in the evaluation of the tularemia vaccine in the "Discussion" section of the manuscript (lines 637-642).

Part III – Minor Issues

Line 74. Mice would be more susceptible to lethal infections with virulent F. tularensis down to 1 CFU (intranasal and subQ/ID). Not sure where <50 CFU (granted 1 CFU would also be <50). A reference or two should be provided here to provide justify this LD50.

We apologize for this inaccuracy. In mice, the lethal dose of virulent strains of F. tularensis consists of only a few bacteria; we have corrected this in the manuscript (lines 78-80) and included references to the relevant studies.
