# Supplementary material for: Immunopeptidomics reveals conserved and abundant Francisella tularensis proteins as immunogenic CD4 T cell antigens in mice
Source: PLoS Pathog. 2026 Sep 29;22(9):e1014597. doi: 10.1371/journal.ppat.1014597 (PMC13649117; doi:10.1371/journal.ppat.1014597)
Supplement: Peer review history

# Peer review: Decision Letter 0

**DOI:** 10.1371/journal.ppat.1014597.r001

**Authors:** Jason Carlyon, David Skurnik

10 Dec 2025

Immunopeptidomics reveals conserved and abundant Francisella tularensis proteins as immunogenic CD4 T cell antigens in mice

PLOS Pathogens

Dear Dr. Link,

Thank you for submitting your manuscript to PLOS Pathogens. After careful consideration, we feel that it has merit but does not fully meet PLOS Pathogens's publication criteria as it currently stands. Therefore, we invite you to submit a revised version of the manuscript that addresses the points raised during the review process.

Please submit your revised manuscript by Feb 08 2026 11:59PM. If you will need more time than this to complete your revisions, please reply to this message or contact the journal office at plospathogens@plos.org. When you're ready to submit your revision, log on to https://www.editorialmanager.com/ppathogens/ and select the 'Submissions Needing Revision' folder to locate your manuscript file.

Please include the following items when submitting your revised manuscript:

* A rebuttal letter that responds to each point raised by the editor and reviewer(s). You should upload this letter as a separate file labeled 'Response to Reviewers'. This file does not need to include responses to any formatting updates and technical items listed in the 'Journal Requirements' section below.

* A marked-up copy of your manuscript that highlights changes made to the original version. You should upload this as a separate file labeled 'Revised Manuscript with Track Changes'.

* An unmarked version of your revised paper without tracked changes. You should upload this as a separate file labeled 'Manuscript'.

If you would like to make changes to your financial disclosure, competing interests statement, or data availability statement, please make these updates within the submission form at the time of resubmission. Guidelines for resubmitting your figure files are available below the reviewer comments at the end of this letter.

We look forward to receiving your revised manuscript.

Kind regards,

Jason A Carlyon, PhD

Academic Editor

PLOS Pathogens

David Skurnik

Section Editor

Editor-in-Chief

PLOS Pathogens

PLOS Pathogens

orcid.org/0000-0002-7699-2064

**Additional Editor Comments:**

Dear Dr. Link,

The reviewers find merit with your study especially that it has identified new highly immunogenic MHC-II-displayed Francisella peptides that can stimulate CD4+ T cells. However, the significance of the current study is impacted by a lack of functional immunity experiments that demonstrate protection. In particular, Reviewer 1's recommendation to test whether immunizing mice with some of these peptides protect/reduce the bacterial burden following Francisella challenge is needed.

**Journal Requirements:**

If the reviewer comments include a recommendation to cite specific previously published works, please review and evaluate these publications to determine whether they are relevant and should be cited. There is no requirement to cite these works unless the editor has indicated otherwise.

1) Please upload all main figures as separate Figure files in .tif or .eps format. For more information about how to convert and format your figure files please see our guidelines:

https://journals.plos.org/plospathogens/s/figures

**Reviewers' Comments:**

Reviewer's Responses to Questions

**Part I - Summary**

Please use this section to discuss strengths/weaknesses of study, novelty/significance, general execution and scholarship.

Reviewer #1: Overall, the paper is a valuable discovery and validation work for Francisella tularensis-specific murine epitopes of two vaccine strains, LVS strain versus the LVSΔdsbA. This work will clearly inform work performed in this mouse model, however, the connection to the human system, and as quoted as motivation, remains a little unclear.

Reviewer #2: In their paper they discuss that vaccination against Francisella using the LVS strain involves the action of CD+ T cell cells, but antigens targeted from these infections are now know. To uncover potential antigens they purified MHC-II and identified displayed bacterial peptides by mass spec. From this they identified peptides from 23 different proteins. They went on to validate many of these peptides as MHC-II displayed, and showed they were capable if stimulating splenocytes and CD4+ T cells. Their results identified new highly immunogenic peptides not previously identified and found some even more immunogenic than previously known targets. Overall, their data seems rigorous with appropriate replicates and well presented. The main thing that hold the paper back is significance due to the lack of functional immunity experiments. Experiments vaccinating with their synthesized peptides and showing protections against Francisella infection or maybe reduced infection burden vs the LVS strain could really boost significance. Short of that, perhaps showing sera from that peptide immunized animals can recognize LVS proteins. The paper would be an accept from me at a more vaccine specific journals or Plos One where similar studies they referenced were published, but without functional protection data their findings are less impactful for a larger Plos Pathogenesis audience.

**********

**Part II – Major Issues: Key Experiments Required for Acceptance**

Please use this section to detail the key new experiments or modifications of existing experiments that should be absolutely required to validate study conclusions.

Generally, there should be no more than 3 such required experiments or major modifications for a "Major Revision" recommendation. If more than 3 experiments are necessary to validate the study conclusions, then you are encouraged to recommend "Reject".

Reviewer #1: As a reader unfamiliar with this pathogen, I found it necessary to look up the host range and human virulence of this pathogen online. The manuscript would benefit from a clearer description of the biological context, motivation, and overall scope of the study. Please expand on how the mouse model relates to human clinical data and specify the species you are working with throughout the manuscript, please. Also, the LVSΔdsbA mutant is not introduced, and it is hard to understand why the authors seek the comparison between wt and mutant strain in the first instance.

In Figure 1, The statement “The presence of specific peptide only (or predominantly) in the infected samples helped to identify the bacterial peptides” is currently unclear and would benefit from further clarification.

Please indicate the exact number of bacterial peptide sequences identified in both infected and uninfected samples. It would be helpful to explain why identifications are obtained from uninfected cells; this observation needs to be clearly communicated to the reader and carefully explained.

You state that most peptides were identified using the match-between-runs feature. This approach is generally discouraged in immunopeptidomics discovery studies. Please provide additional evidence supporting that these peptides are genuinely present in the samples or clarify how this analytical choice affects confidence in the identifications.

The authors should include a brief discussion of the observed differences in length distribution between self-derived and bacterial peptides - I did not see this mentioned in the text, or discussed.

Conservation results section. Page 11, line 201. The statement “…were unique to the bacterium…” could be clarified. Does this refer to peptides unique to the bacterial strain? Presumably, peptides with sequences identical to those in the mouse proteome would be excluded from bacterial assignments. Please revise this section to make this point explicit. A short motivation in why you are carrying out this analysis would be helpful to the reader.

My understanding is that the term “epitope region” refers to overlapping MHC-II–bound peptide sequences, which are characteristic of class II presentation. Please confirm if this interpretation is correct and consider clarifying this definition in the text more clearly. Including a schematic or example figure would help prevent potential confusion.

In the section “Francisella MHC-II antigens originate from a highly abundant fraction of the bacterial proteome” and in the corresponding conclusion, the claim that “Francisella antigens are sampled into the MHC class II pathway from a highly abundant fraction of its proteome” may be overstated. The data likely reflect the sensitivity limits of the immunopeptidomics experiment, which may favor detection of peptides from more abundant proteins. Please discuss this limitation and temper the conclusion accordingly.

Reviewer #2: Impact of study limited by lack of functional immunity experiments to wrap it up. While their results find the peptides are immunogenic, they don’t show protection. Experiments vaccinating mice with the peptides could boost the studies impact.

**********

**Part III – Minor Issues: Editorial and Data Presentation Modifications**

Please use this section for editorial suggestions as well as relatively minor modifications of existing data that would enhance clarity.

Reviewer #1: On page 8, line 155, the relative proportion of predicted bacterial peptide binders compared to self-peptides is not clearly described or visualized. Please quantify this comparison and, if possible, illustrate it explicitly in a figure.

Reviewer #2: (No Response)

**********

PLOS authors have the option to publish the peer review history of their article (what does this mean?). If published, this will include your full peer review and any attached files.

If you choose “no”, your identity will remain anonymous but your review may still be made public.

**Do you want your identity to be public for this peer review?**  For information about this choice, including consent withdrawal, please see our Privacy Policy.

Reviewer #1: No

Reviewer #2: No

**Figure resubmission:**

**Reproducibility:**

?>

# Author comment: Author response to Decision Letter 1

**DOI:** 10.1371/journal.ppat.1014597.r002

17 Jun 2026

Attachment Submitted filename: Response to Reviewers.pdf

# Peer review: Decision Letter 1

**DOI:** 10.1371/journal.ppat.1014597.r003

**Authors:** Jason Carlyon, David Skurnik

24 Jul 2026

PPATHOGENS-D-25-02289R1

Immunopeptidomics reveals conserved and abundant Francisella tularensis proteins as immunogenic CD4 T cell antigens in mice

PLOS Pathogens

Dear Dr. Link,

Thank you for submitting your manuscript to PLOS Pathogens. After careful consideration, we feel that it has merit but does not fully meet PLOS Pathogens's publication criteria as it currently stands. Therefore, we invite you to submit a revised version of the manuscript that addresses the points raised during the review process.

Please submit your revised manuscript by Sep 22 2026 11:59PM. If you will need more time than this to complete your revisions, please reply to this message or contact the journal office at plospathogens@plos.org. When you're ready to submit your revision, log on to https://www.editorialmanager.com/ppathogens/ and select the 'Submissions Needing Revision' folder to locate your manuscript file.

Please include the following items when submitting your revised manuscript:

* A letter that responds to each point raised by the editor and reviewer(s). You should upload this letter as a separate file labeled 'Response to Reviewers'. This file does not need to include responses to any formatting updates and technical items listed in the 'Journal Requirements' section below.

* A marked-up copy of your manuscript that highlights changes made to the original version. You should upload this as a separate file labeled 'Revised Manuscript with Track Changes'.

* An unmarked version of your revised paper without tracked changes. You should upload this as a separate file labeled 'Manuscript'.

If you would like to make changes to your financial disclosure, competing interests statement, or data availability statement, please make these updates within the submission form at the time of resubmission. Guidelines for resubmitting your figure files are available below the reviewer comments at the end of this letter.

As the corresponding author, your ORCID iD is verified in the submission system and will appear in the published article. PLOS supports the use of ORCID, and we encourage all coauthors to register for an ORCID iD and use it as well. Please encourage your coauthors to verify their ORCID iD within the submission system before final acceptance, as unverified ORCID iDs will not appear in the published article. Only the individual author can complete the verification step; PLOS staff cannot verify ORCID iDs on behalf of authors.

We look forward to receiving your revised manuscript.

Kind regards,

Jason A Carlyon, PhD

Academic Editor

PLOS Pathogens

David Skurnik

Section Editor

PLOS Pathogens

Sumita Bhaduri-McIntosh

Editor-in-Chief

PLOS Pathogens

orcid.org/0000-0003-2946-9497

Michael Malim

Editor-in-Chief

PLOS Pathogens

orcid.org/0000-0002-7699-2064

**Additional Editor Comments (if provided):**

**Reviewers' Comments:**

Reviewer's Responses to Questions

**Part I - Summary**

Please use this section to discuss strengths/weaknesses of study, novelty/significance, general execution and scholarship.

Reviewer #2: The new vaccination data didn't provide strong protection, but the reduced burden in the organs is encouraging. Overall presents valuable information for the field. I have some minor concerns/questions about number or animals and significance, but feel the paper is improved with the new findings and edits.

Reviewer #3: The submission is a revised manuscript that identifies epitopes from F. tularensis and shown to be immunogenic in mouse models. However, when vaccinating mice with the best performing peptides and then challenging subcutaneously with the FSC200 strain, no differences in protection were observed as compared to naive mice. However, when looking at CFU recovery rates from organs of the challenged mice, a statistical difference in reduced recovery was observed with the vaccinated mice. As not an original reviewer, the authors appeared to have responded to the previous reviewers request. However, this reviewer would suggest some additional revisions though not laboratory work based.

**********

**Part II – Major Issues: Key Experiments Required for Acceptance**

Please use this section to detail the key new experiments or modifications of existing experiments that should be absolutely required to validate study conclusions.

Generally, there should be no more than 3 such required experiments or major modifications for a "Major Revision" recommendation. If more than 3 experiments are necessary to validate the study conclusions, then you are encouraged to recommend "Reject".

Reviewer #2: In line 488-489 They state that the vaccinated group weight loss was delayed. Given the small number of animals and high variability I'm concerned about the significance there. I suggest either rewording or determining significance.

In figures 6 and 7 the number of mice between control and treatment groups are different. Why the mismatch

Reviewer #3: Much effort has also been placed in the T cell responses to the Schu S4 clpB mutant. It would be helpful to incorporate the results found with this vaccine strain and how it relates to the work performed here.

Protection studies with immunized mice and FSC200 challenge:

A) What is the rationale of performing challenges by subQ versus intranasal (pneumonic model). Obviously, this relates to the public health threat versus biodefense. This should be included. Do the authors believe the results would have been any different with an intranasal challenge?

B) The one area that should be addressed in this manuscript is the issue of using mice for tularemia vaccine studies. It is well established that mice are not ideal for vaccines versus establishing virulence differences. Obviously despite these limitations, mice are still a starting point for vaccine studies due to numerous reasons (mostly financial). Perhaps the authors should consider performing these protection studies in rats, a better model for tularemia vaccines versus mice. This could be a next step, so the authors should expand on this point (lines 622-626)

**********

**Part III – Minor Issues: Editorial and Data Presentation Modifications**

Please use this section for editorial suggestions as well as relatively minor modifications of existing data that would enhance clarity.

Reviewer #2: (No Response)

Reviewer #3: Line 74. Mice would be more susceptible to lethal infections with virulent F. tularensis down to 1 CFU (intranasal and subQ/ID). Not sure where <50 CFU (granted 1 CFU would also be <50). A reference or two should be provided here to provide justify this LD50.

**********

PLOS authors have the option to publish the peer review history of their article (what does this mean?). If published, this will include your full peer review and any attached files.

If you choose “no”, your identity will remain anonymous but your review may still be made public.

**Do you want your identity to be public for this peer review?**  For information about this choice, including consent withdrawal, please see our Privacy Policy.

Reviewer #2: No

Reviewer #3: No

[NOTE: If reviewer comments were submitted as an attachment file, they will be attached to this email and accessible via the submission site. Please log into your account, locate the manuscript record, and check for the action link "View Attachments". If this link does not appear, there are no attachment files.]

**Figure resubmission:**

**Reproducibility:**

To enhance the reproducibility of your results, we recommend that authors of applicable studies deposit laboratory protocols in protocols.io, where a protocol can be assigned its own identifier (DOI) such that it can be cited independently in the future. Additionally, PLOS ONE offers an option to publish peer-reviewed clinical study protocols. Read more information on sharing protocols at https://plos.org/protocols?utm_medium=editorial-email&utm_source=authorletters&utm_campaign=protocols

# Author comment: Author response to Decision Letter 2

**DOI:** 10.1371/journal.ppat.1014597.r004

28 Aug 2026

Attachment Submitted filename: Response to Reviewers_rev2.pdf

# Editor report: Decision Letter 2

**DOI:** 10.1371/journal.ppat.1014597.r005

**Authors:** Jason Carlyon, David Skurnik

4 Sep 2026

Dear Mr Link,

We are pleased to inform that your manuscript, "Immunopeptidomics reveals conserved and abundant Francisella tularensis proteins as immunogenic CD4 T cell antigens in mice", has been editorially accepted for publication at PLOS Pathogens.

Before your manuscript can be formally accepted and sent to production, you will need to complete our formatting changes, which you will receive by email within a week. Please note that your manuscript will not be scheduled for publication until you have made the required changes.

IMPORTANT NOTES

(1) Copyediting and Proofreading: The corresponding author will receive a typeset proof for review, to ensure errors have not been introduced during production. Please review the PDF proof of your manuscript carefully, as this is the last chance to correct any errors. Please note that major changes, or those which affect the scientific understanding of the work, will likely cause delays to the publication date of your manuscript.

(2) Appropriate Figure Files: Please remove all name and figure # text from your figure files. Please also take this time to check that your figures are of high resolution, which will improve the readbility of your figures and help expedite your manuscript's publication. Please note that figures must have been originally created at 300dpi or higher. Do not manually increase the resolution of your files. For instructions on how to properly obtain high quality images, please review our Figure Guidelines, with examples at: http://journals.plos.org/plospathogens/s/figures.

We encourage you to use PACE (http://pace.apexcovantage.com/), a figure-checking tool which helps authors ensure all figures meet PLOS requirements so that figures can publish in the highest quality. PACE is a digital diagnostic and conversion tool for figure files. It will provide information about any failed check(s) and, if able, will automatically convert the figure into an acceptable file that passes quality checks.

To use PACE, you must first register as a user. Then, login and navigate to the UPLOAD tab, where you will find detailed instructions on how to use the tool.

(3) Striking Image: Please upload a striking still image to accompany your article if one is available (you can include a new image or an existing one from within your manuscript). Should your paper be accepted, this image will be considered for our monthly issue image and may also appear on our website to feature your article. Please upload this as a separate file, selecting "striking image" as the file type upon upload. Please also include a separate "Other" file with a caption, including credits and any potential copyright information. Please do not include the caption in the main article file. If your image is from someone other than yourself, please ensure that the artist has read and agreed to the terms and conditions of the Creative Commons Attribution License at http://journals.plos.org/plospathogens/s/content-license. Please note that PLOS cannot publish copyrighted images.

(4) Press Release or Related Media: If you or your institution will be preparing press materials for this manuscript, please inform our press team in advance at plospathogens@plos.org as soon as possible. We ask that you contact us within one week to plan ahead of our fast Production schedule. If you need to know your paper's publication date for related media purposes, you must coordinate with our press team, and your manuscript will remain under a strict press embargo until the publication date and time.

(5) Update your Profile Information: Now that your manuscript has been provisionally accepted, please log into Editorial Manager and update your profile, if needed. Go to http://www.editorialmanager.com/ppathogens, log in, and click on the "Update My Information" link at the top of the page. Please update your user information to ensure an efficient production and billing process.

(6) LaTeX users only: Our staff will ask you to upload a TEX file in addition to the PDF before the paper can be sent to typesetting, so please carefully review our Latex Guidelines http://journals.plos.org/plospathogens/s/latex in the meantime.

Thank you again for supporting Open Access publishing; we are looking forward to publishing your work in PLOS Pathogens.

Best regards,

Jason A Carlyon, PhD

Academic Editor

PLOS Pathogens

David Skurnik

Section Editor

PLOS Pathogens

Dominique Soldati-Favre

Editor-in-Chief

PLOS Pathogens

***********************************************************

# Editor report: Acceptance letter

**DOI:** 10.1371/journal.ppat.1014597.r006

**Authors:** Jason Carlyon, David Skurnik

Dear Mr Link,

We are delighted to inform you that your manuscript, "Immunopeptidomics reveals conserved and abundant Francisella tularensis proteins as immunogenic CD4 T cell antigens in mice," has been formally accepted for publication in PLOS Pathogens.

We have now passed your article onto the PLOS Production Department who will complete the rest of the pre-publication process. All authors will receive a confirmation email upon publication.

Thank you again for supporting open-access publishing; we are looking forward to publishing your work in PLOS Pathogens.

Best regards,

Dominique Soldati-Favre

Editor-in-Chief

PLOS Pathogens
